# Computational Modeling of Substrate-Dependent Mitochondrial Respiration and Bioenergetics in the Heart and Kidney Cortex and Outer Medulla

**DOI:** 10.1093/function/zqad038

**Published:** 2023-07-25

**Authors:** Shima Sadri, Xiao Zhang, Said H Audi, Allen W Cowley Jr., Ranjan K Dash

**Affiliations:** Department of Biomedical Engineering, Medical College of Wisconsin, Milwaukee, WI 53226, USA; Department of Biomedical Engineering, Medical College of Wisconsin, Milwaukee, WI 53226, USA; Department of Biomedical Engineering, Medical College of Wisconsin, Milwaukee, WI 53226, USA; Department of Biomedical Engineering, Marquette University, Milwaukee, WI 53223, USA; Department of Physiology, Medical College of Wisconsin, Milwaukee, WI 53226, USA; Cardiovascular Research Center, Medical College of Wisconsin, Milwaukee, WI 53226, USA; Department of Biomedical Engineering, Medical College of Wisconsin, Milwaukee, WI 53226, USA; Department of Biomedical Engineering, Marquette University, Milwaukee, WI 53223, USA; Department of Physiology, Medical College of Wisconsin, Milwaukee, WI 53226, USA; Cardiovascular Research Center, Medical College of Wisconsin, Milwaukee, WI 53226, USA

**Keywords:** mitochondrial metabolism, forward and reverse electron transfer, respiration and bioenergetics, oxidative phosphorylation, substrate utilization, computational modeling

## Abstract

Integrated computational modeling provides a mechanistic and quantitative framework to characterize alterations in mitochondrial respiration and bioenergetics in response to different metabolic substrates *in-silico*. These alterations play critical roles in the pathogenesis of diseases affecting metabolically active organs such as heart and kidney. Therefore, the present study aimed to develop and validate thermodynamically constrained integrated computational models of mitochondrial respiration and bioenergetics in the heart and kidney cortex and outer medulla (OM). The models incorporated the kinetics of major biochemical reactions and transport processes as well as regulatory mechanisms in the mitochondria of these tissues. Intrinsic model parameters such as Michaelis–Menten constants were fixed at previously estimated values, while extrinsic model parameters such as maximal reaction and transport velocities were estimated separately for each tissue. This was achieved by fitting the model solutions to our recently published respirometry data measured in isolated rat heart and kidney cortex and OM mitochondria utilizing various NADH- and FADH_2_-linked metabolic substrates. The models were validated by predicting additional respirometry and bioenergetics data, which were not used for estimating the extrinsic model parameters. The models were able to predict tissue-specific and substrate-dependent mitochondrial emergent metabolic system properties such as redox states, enzyme and transporter fluxes, metabolite concentrations, membrane potential, and respiratory control index under diverse physiological and pathological conditions. The models were also able to quantitatively characterize differential regulations of NADH- and FADH_2_-linked metabolic pathways, which contribute differently toward regulations of oxidative phosphorylation and ATP synthesis in the heart and kidney cortex and OM mitochondria.

## Graphical Abstract

**Graphical Abstract:**
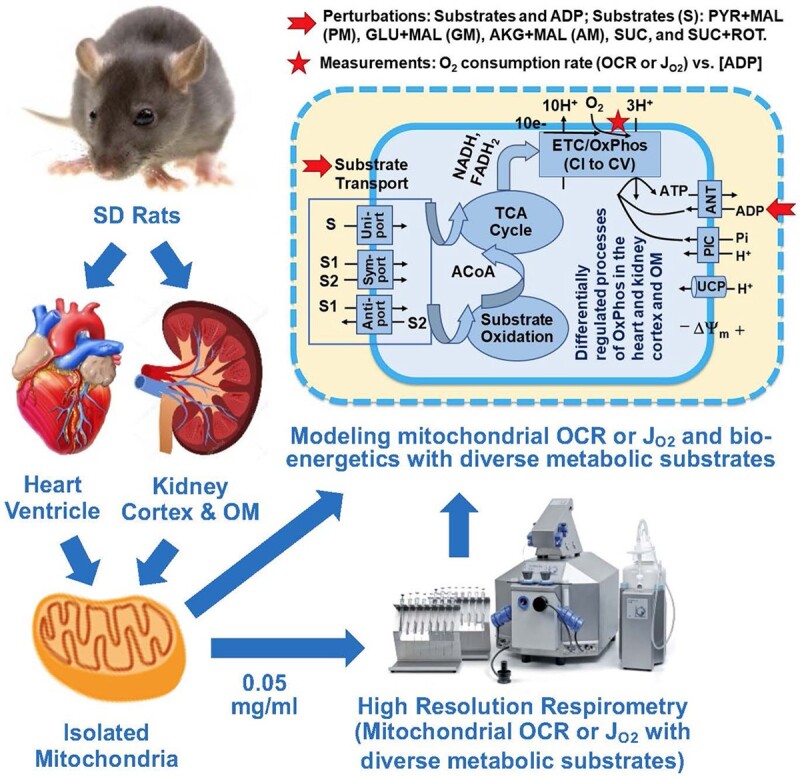
Graphical overview of the combined experimental and computational modeling approach to characterize substrate-dependent mitochondrial respiration and bioenergetics in the heart and kidney cortex and outer medulla.

## Introduction

Heart and kidney are the most metabolically active organs with the highest mitochondrial contents, metabolic rates, and oxygen consumptions in the human body.^1–5^ They carry out distinct metabolic functions essential for the survival of the organism. Consequently, metabolic dysfunctions in these organs can lead to an array of cardiovascular and renal diseases, including salt-sensitive (SS) hypertension.^6–14^ Considering the relatively large energy demand in these organs and their dependency of energy production upon mitochondrial respiration and bioenergetics, it is very important to systematically characterize mitochondrial respiration and bioenergetics in these organs and identify potential differences and underlying mechanisms.^1^ Such information is highly essential for computational modeling, needed for a mechanistic and quantitative understanding of the role of mitochondrial respiration and bioenergetics in the pathogenesis of metabolic diseases.

Both the heart and kidney derive nearly 95% of their energy adenosine triphosphate (ATP) form mitochondrial oxidative phosphorylation (OxPhos).^1,3,4,8^ The primary substrate for energy production in the heart is free fatty acids.^8^,^15–17^ On the other hand, both free fatty acids and ketone bodies are primarily utilized for energy production in the proximal tubules (PT) of the kidney cortex, whereas glucose is primarily utilized for energy production in the medullary thick ascending limbs (mTAL) of the kidney outer medulla (OM).^2,3,6,14,18,19^ For a given tissue, alteration in the primary substrate(s) can result in changes in the kinetics and efficiencies of mitochondrial energy production.^1,20^ Mechanistic and quantitative characterization of how different substrates regulate mitochondrial respiration and bioenergetics in the selected tissues is an important step toward understanding how changes in the kinetics and efficiencies of mitochondrial energy production may contribute to tissue/organ dysfunctions and disease processes.^1^

Significant differences have been found in mitochondrial enzyme activities, substrate utilization, respiration, and bioenergetics between the heart and kidney cortex and OM under physiological and pathological conditions.^1,6,10,14^ Using 3D optical fluorescence cryoimaging technique, Salehpour et al.^21^ found that kidney OM, but not kidney cortex, exhibits a decreased NADH/FAD redox ratio in the Dahl SS hypertensive rat fed a high salt diet. The same study also indicated that kidney cortex and OM must be treated as 2 distinct tissues and that their mitochondrial respiration and bioenergetics be studied separately. In a recent study,^1^ we have found that different metabolic substrates produce significantly different respiratory and bioenergetic responses in isolated mitochondria from the normal Sprague–Dawley (SD) rat heart and kidney cortex and OM, which signify substrate-dependent distinct kinetics and efficiency of OxPhos for ATP production in these tissues. Data from that study have enabled the development and validation of integrated computational models of mitochondrial respiration and bioenergetics to elucidate the distinct emergent metabolic system properties of mitochondria in these tissues, which is the focus of the present study.

Tissue-specific changes in mitochondrial respiration, bioenergetics, redox states, and substrate utilization have been observed in the progression of both cardiac and renal diseases.^5,6,8,10^,^15–17^,^21,22^ Although impaired mitochondrial substrate and energy metabolism has been directly linked to the deterioration of heart and kidney functions, the exact substrate-dependent network of transporters and enzymes responsible for the metabolic dysfunctions remains unclear. Moreover, a systematic tissue-specific and substrate-dependent characterization is lacking, which is required for a mechanistic and quantitative understanding of the relationships between altered substrate oxidation, altered mitochondrial metabolism, and the progression of cardiac and renal diseases.

Integrated computational modeling provides a mechanistic and quantitative framework for characterizing *in-silico* changes within a given metabolic network to better understand complex interactions and regulations resulting from mitochondrial metabolic alterations under physiological and pathological conditions.^23–25^ Computational models of mitochondrial respiration and bioenergetics have been developed for different levels of biological complexity by various groups (eg, see ref. ^24–36^ and the “Discussion” section for an extensive review of existing models and their relevance). Those models have enabled modelers to zoom in and out of a given network of processes and quantitatively understand mitochondrial metabolic responses at levels ranging from single proteins (enzymes and transporters) to a network of interacting proteins. Although several models of mitochondrial electron transport chain (ETC), tricarboxylic acid (TCA) cycle, and metabolite and cation transporters regulating OxPhos and ATP synthesis have been developed, integrated models comparing mitochondrial respiratory and bioenergetic responses to different metabolic substrates in different tissues/organs such as the heart vs. kidney are lacking. As such, none of the existing models can describe tissue-specific and substrate-dependent responses of mitochondrial respiration and bioenergetics observed in our recent experimental study.^1^ In addition, although there are several such models for the heart and skeletal muscle mitochondria,^24–27^,^29–36^ there is a scarcity of such models for the kidney mitochondria.

Efforts toward this end were made recently by Edwards et al.^37^ by modifying a previously developed cardiac mitochondrial respiration and bioenergetics model^35^ by adjusting several model parameters relevant to kidney anatomy and physiology. Although their modified model can simulate kidney mitochondrial oxygen consumption (respiration) and ATP generation in the rat PT cells, model simulations were not fitted to any experimental data nor the mitochondrial respiratory and bioenergetic responses to different metabolic substrates were studied. Previously, we have developed a thermodynamically constrained integrated computational model of rat lung tissue mitochondrial respiration and bioenergetics, which was parameterized and validated using in-house and published data using different metabolic substrates,^28^ and hence providing the foundation for the present computational modeling work.

**Figure 1.**
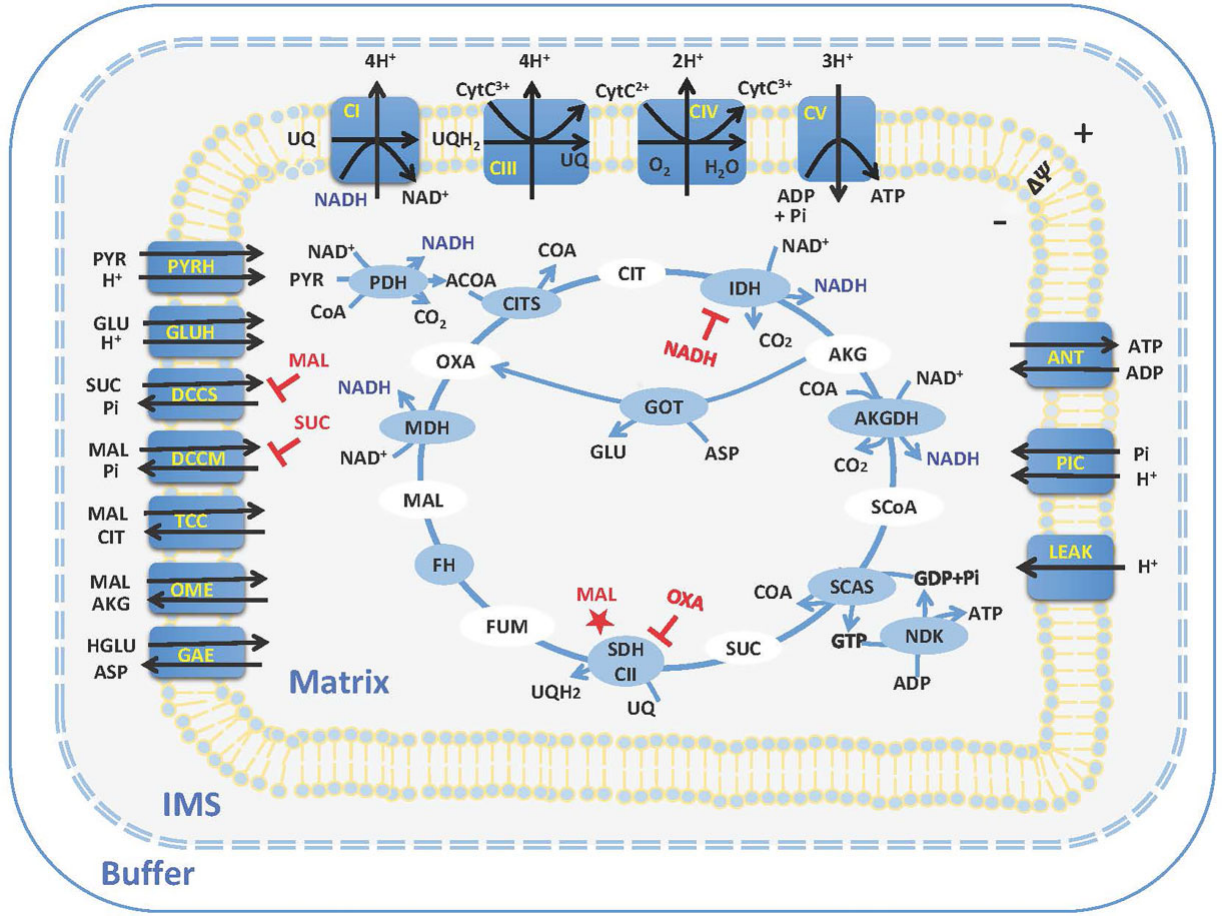
Schematics of the proposed isolated mitochondria model. The model consists of 3 regions (buffer, inter-membrane space (IMS), and matrix). The model includes metabolite and phosphate transporters, H^+^ leak, TCA cycle enzymes, ETC complexes, and ATP synthase. Mitochondrial complex II is lumped as part of the TCA cycle shown as SDH-CII. The transporters and enzymes are shown in blue circles, and the metabolite in the TCA cycle are shown in white circles. All the metabolites except CytC are assumed to be freely permeable across mitochondria outer membrane. The metabolites are transported between the mitochondrial matrix and IMS regions by different transports, including uniporters, co-transporters, and antitransporters. The model regulations including dicarboxylate carrier for SUC_e_ influx (DCCS) inhibition by MAL_m_, dicarboxylate carrier for MAL_m_ efflux (DCCM) inhibition by SUC_m_, IDH inhibition by NADH, SDH-CII inhibition by OXA_m_, and SDH-CII stimulation by MAL_m_.

Starting with our recent rat lung tissue mitochondrial model,^28^ in the present study, we have developed and validated 3 parallel computational models to study *in-silico* respiration and bioenergetics of mitochondria isolated from normal SD rat heart and kidney cortex and OM. These models account for the kinetics of mitochondrial metabolites and phosphates transporters, adenine nucleotide translocase (ANT) for ATP/ADP exchange, proton (H^+^) leak, TCA cycle reactions, ETC reactions and OxPhos, and tissue-specific regulations of NADH- and FADH_2_-linked metabolic pathways (Figure 1). The models were parameterized based on a systematic and well-controlled dataset obtained recently in our laboratory,^1^ enabling us to study *in-silico* mitochondrial respiration and bioenergetics in the presence of NADH-linked metabolic substrates including pyruvate + malate, glutamate + malate, and alpha-ketoglutarate + malate, and FADH_2_-linked metabolic substrate succinate in the absence or presence of rotenone (complex I inhibitor) in these tissues. To the best of our knowledge, the present models represent the first data-driven attempts to characterize *in-silico* the respiratory and bioenergetic responses of the heart and kidney cortex and OM mitochondria to both NADH- and FADH_2_-linked metabolic substrates. These models serve as valuable tools for identifying new therapeutic targets and modifying mitochondrial substrate and energy metabolism that affects the development of metabolic disorders in metabolically active organs such as the heart and kidney.

## Materials and Methods

### Experimental Data

The mitochondrial models were parametrized by fitting the model solutions to mitochondrial oxygen consumption rate (OCR or *J*_O2_ flux; respiration) data acquired using the experimental protocol of Figure S1A and validated by predicting the mitochondrial OCR and membrane potential (${\rm{\Delta \Psi }}$_m_) data acquired using the experimental protocol of Figure S1B. Mitochondria were isolated from normal SD rat heart and kidney cortex and OM tissues by differential centrifugation. The mitochondrial OCR were measured via an Oxygraph-2k (O2k) respirometer (Oroboros Instrument, Innsbruck, Austria) at 37°C, and ${\rm{\Delta \Psi }}$_m_ were measured via a Photon Technology International (PTI) spectrofluorometer (Horiba Scientific Inc.) using the cationic fluorescent dye rhodamine-123 (R123) at 37°C and calibrated, as detailed in Tomar et al.^1^ In Figure S1A experimental protocol (used for fitting), 0.05 mg/mL of heart mitochondria or 0.2 mg/mL of kidney cortex or OM mitochondria were added to the O2k chamber. Then, one of the following substrate combinations: pyruvate + malate (PYR + MAL or PM; 5:2.5 m m), glutamate + malate (GLU + MAL or GM; 5:2.5 m m), alpha-ketoglutarate + malate (AKG + MAL or AM; 5:2.5 m m), and succinate in the absence or presence of rotenone (SUC ± ROT; 10 m m ± 0.5 µm) was added followed by the sequential addition of increasing ADP concentrations of 25, 50, 75, 100, 150, and 250 µm. In Figure S1B experimental protocol (used for model validation), 0.1 mg/mL of heart mitochondria or 0.2 mg/mL of kidney cortex or OM mitochondria were added to the O2k chamber. Then, one of the following substrate combinations: PM (5:2.5 m m), GM (5:2.5 m m), AM (5:2.5 m m), and SUC ± ROT (10 m m ± 0.5 µm) was added followed by the addition of 200 µm ADP to the heart mitochondria and 100 µm ADP to the kidney cortex and OM mitochondria.

### Computational Model

The present computational models of mitochondrial respiration and bioenergetics for the heart and kidney cortex and OM were developed starting with our previously developed thermodynamically constrained mechanistic modeling framework and computational model of isolated lung tissue mitochondrial respiration and bioenergetics.^28^ The lung tissue mitochondrial model was modified by adding multiple regulation mechanisms specific to the heart and kidney mitochondrial respiration and bioenergetics (eg, see the “Modeling SUC-pathway mediated regulations” section) based on our recently published experimental data.^1^ As shown in Figure 1, each of the 3 proposed models consists of 3 different regions: extra-mitochondrial buffer region, mitochondria matrix region, and intermembrane space (IMS) region. In addition, each model includes the kinetics of 24 reaction and transport fluxes and the dynamics of 37 state variables (36 metabolite concentrations and mitochondrial membrane potential). The outer mitochondrial membrane (OMM) has porins, and hence, it is assumed to be freely permeable to most of the metabolites, except cytochrome c, which has a relatively high molecular weight. In addition, cytochrome c is assumed to exist in the IMS along the inner mitochondrial membrane (IMM). The model does not account for the dynamics of cations (eg, H^+^, K^+^, Na^+^, Mg^2+^, and Ca^2+^). Rather, cations are assumed to be fixed at their stipulated values in the different regions of the model, based on the well-controlled experimental conditions of Tomar et al.^1^ The relative volumes of different regions of the model are determined based on the amount of mitochondrial protein used in an experiment, which are provided in Table 1, along with the levels of conserved metabolite pools within the mitochondrial matrix. The details of the model state variables, enzymatic reactions and transporters, flux expressions and the associated kinetic parameters, and mass balance-based ordinary differential equations (ODEs) and the associated initial conditions for the state variables are provided in Supplementary Material S1.

**Table 1.** General Model Parameter Values

Parameter	Value	Source
Buffer volume (*V*_e_)	1 or 2 mL	Experimental condition
Mitochondria matrix volume (*V*_m_)	1 μL/mg mitochondria protein	28, 31–35
Mitochondria IMS volume (*V*_ims_)	Assume to be 10% of *V*_m_	28, 31–35
Total cytochrome c concentration (CytCred + CytCox)	1 m m	28, 31–35
Total pyridine nucleotide concentration (NAD + NADH)	3 m m	28, 31–35, 80, 81
Total ubiquinone concentration (UQ + UQH_2_)	1.5 m m	28, 31–35
Total adenine nucleotide content (ATP and ADP)	10 m m	28, 31–35
Total coenzyme A content (SCoA + ACoA + CoA)	1 m m	28, 31, 35, 80, 81

#### Mitochondrial TCA Cycle and ETC Reactions

The model accounts for 9 TCA cycle reaction fluxes responsible for metabolite oxidation and NADH and/or FADH_2_ generation, which subsequently provide electrons for the ETC reactions responsible for OxPhos and ultimately ATP production. A general form of multisubstrate multiproduct enzymatic reaction is described as:


(1) \begin{eqnarray*} {\alpha }_1{S}_1 + {\alpha }_2{S}_2 + ... + {\alpha }_{Ns}{S}_{Ns} \rightleftharpoons {\beta }_1{P}_1 + {\beta }_2{P}_2 + ... + {\beta }_{Np}{P}_{Np}, \end{eqnarray*}

where *S_i_* is the *i*^th^ substrate; *P_j_* is the *j*^th^ product; *Ns* and *Np* are the number of substrates and products, respectively; *α_i_* and *β_j_* are the stoichiometry coefficients corresponding to the substrate *S*_i_ and product *P*_j_, respectively. The corresponding reaction flux (*J*_RXN_) is described by the following general Michaelis–Menten equation, which is based on a generalized random-ordered rapid-equilibrium kinetic mechanism^28^:


(2) \begin{eqnarray*} {J}_{RXN} = \frac{{\frac{{{V}_{\max f}}}{{\mathop \prod \nolimits_{i = 1}^{{N}_s} {K}_{{S}_i^{}}^{{\alpha }_i}}}\left( {\mathop \prod \nolimits_{i = 1}^{{N}_s} C{{_{{S}_i}^{}}}^{{\alpha }_i} - \frac{{\mathop \prod \nolimits_{j = 1}^{{N}_p} C{{_{{P}_j}^{}}}^{{\beta }_j}}}{{{{K^{\prime}}}_{eq}}}} \right)}}{{\mathop \prod \nolimits_{i = 1}^{{N}_s} \left( {1 + \frac{{C{{_{{S}_i}^{}}}^{{\alpha }_i}}}{{{K}_{{S}_i^{}}^{{\alpha }_i}}}} \right) \times \mathop \prod \nolimits_{j = 1}^{{N}_p} \left( {1 + \frac{{C{{_{{P}_j}^{}}}^{{\beta }_j}}}{{{K}_{{P}_j^{}}^{{\beta }_j}}}} \right)}}, \end{eqnarray*}

where *V_maxf_* is the maximum forward reaction rate; *C_Si_* and *C_Pi_* are the concentrations of the substrate *S_i_* and product *P_j_*, respectively; and *K_Si_* and *K_Pj_* are the binding constants corresponding to the substrate *S*_i_ and product *P*_j_, respectively; ${K^{\prime}}_{eq}$ is the apparent equilibrium constant of the reaction at specified thermodynamic conditions (ie, temperature, ionic strength, and pH), which is the ratio of the forward and reverse rate constants of the reaction and is equal to the mass action ratio at equilibrium. The corresponding equilibrium constant at pH = 7, ${K^{\prime}}^0_{eq}$, is related to the transformed Gibb’s free energy ${\Delta }_rG^{{\prime}^0}$ of the reaction at pH = 7, and is defined as:


(3) \begin{eqnarray*} K^{\prime^0}_{eq} = \frac{\prod\nolimits_{j=1}^{N_P}C_{{P_j},eq}^{\,\,\,\,\,\,\,\,\,\,\,\,\beta_j}}{\prod\nolimits_{i=1}^{N_S}C_{{S_i},eq}^{\,\,\,\,\,\,\,\,\,\,\,\,\alpha_i}} = \exp \left(\frac{-{\Delta_rG^{\prime^0}}}{RT} \right ). \end{eqnarray*}

${K^{\prime}}_{eq}$ for a proton producing and consuming reaction at a specified pH can be calculated using eqns (4) and (5), respectively:


(4) \begin{eqnarray*} {K^{\prime}_{eq}} = K_{eq}^{{\prime 0}} \times {10}^{nH(p{H}_m - 7)} = {e}^{- {{\Delta}_rG^{\prime^0}/RT}} \times {10}^{nH(p{H}_m - 7)}, \end{eqnarray*}

(5) \begin{eqnarray*} {K^{\prime}_{eq}} = K_{eq}^{{\prime 0}} \times {10}^{nH(7 - p{H}_m)} = {e}^{- {{\Delta}_rG^{\prime^0}/RT}} \times {10}^{nH(7 - p{H}_m)}, \end{eqnarray*}

where *R* is the ideal gas constant (8.314 J/mole/Kelvin), *T* is the absolute temperature (310.15 Kelvin), *nH* is the number of protons produced or consumed in the reaction, and pH_m_ is the mitochondrial matrix pH = 7.6. The flux expressions and the associated kinetic parameter values for individual enzymatic reactions in the integrated mitochondrial model are defined in the Supplementary Material S2.

#### Mitochondrial Metabolite Transporters

The model accounts for 10 metabolites and phosphates transporters responsible for the transport of TCA cycle metabolites, adenine nucleotides, and inorganic phosphate between the IMS [assumed to be equilibrated with the extra-mitochondrial (buffer) region] and mitochondrial matrix. We used the following general equations to describe the fluxes of different types of transporters including uniporters (eqn 7), symporters or co-transporters (eqn 9), and antiporters or exchangers (eqn 11) involving neutral compounds. For transport of charged compounds (eg, ATP/ADP exchange) and pumps (eg, ETC complexes), the effect of mitochondrial membrane potential is appropriately incorporated into the equations (see Supplementary Material S3).


(6) \begin{eqnarray*} {\rm{Uniporter:}}\,\,{A}_e \rightleftharpoons {A}_m, \end{eqnarray*}

(7) \begin{eqnarray*} {J}_{e \leftrightarrow m,UP} = {T}_{\max f}{{\left( {\frac{{{C}_{Ae} - {C}_{Am}}}{{{K}_{Ae}}}} \right)} \mathord{\left/ {\vphantom {{\left( {\frac{{{C}_{Ae} - {C}_{Am}}}{{{K}_{Ae}}}} \right)} {\left( {1 + \frac{{{C}_{Ae}}}{{{K}_{Ae}}} + \frac{{{C}_{Am}}}{{{K}_{Am}}}} \right)}}} \right. } {\left( {1 + \frac{{{C}_{Ae}}}{{{K}_{Ae}}} + \frac{{{C}_{Am}}}{{{K}_{Am}}}} \right)}}, \end{eqnarray*}

(8) \begin{eqnarray*} {\rm{Symporter:}}\,\,\,{A}_e + {B}_e \rightleftharpoons {A}_m + {B}_m, \end{eqnarray*}

(9) \begin{eqnarray*} && {J}_{e \leftrightarrow m,SP} = {T}_{\max f}{{\left( {\frac{{{C}_{Ae}{C}_{Be} - {C}_{Am}{C}_{Bm}}}{{{K}_{Ae}{K}_{Be}}}} \right)}}\\ &&\qquad \big/{\left(1 + \frac{{C}_{Ae}}{{K}_{Ae}} + \frac{{C}_{Be}}{{K}_{Be}} + \frac{{C}_{Am}}{{K}_{Am}} + \frac{{C}_{Bm}}{{K}_{Bm}} + \frac{{C}_{Ae}{C}_{Be}}{{K}_{Ae}{K}_{Be}} + \frac{{C}_{Am}{C}_{Bm}}{{K}_{Am}{K}_{Bm}} \right )},\\ \end{eqnarray*}

(10) \begin{eqnarray*} {\rm{Antiporter:}}\,\,\,{A}_e + {B}_m \rightleftharpoons {A}_m + {B}_e, \end{eqnarray*}

(11) \begin{eqnarray*} && {J}_{e \leftrightarrow m,AP} = {T}_{\max f}{{\left( {\frac{{{C}_{Ae}{C}_{Bm} - {C}_{Am}{C}_{Be}}}{{{K}_{Ae}{K}_{Bm}}}} \right)}}\\ &&\qquad \big/{\left(1 + \frac{{C}_{Ae}}{{K}_{Ae}} + \frac{{C}_{Be}}{{K}_{Be}} + \frac{{C}_{Am}}{{K}_{Am}} + \frac{{C}_{Bm}}{{K}_{Bm}} + \frac{{C}_{Ae}{C}_{Bm}}{{K}_{Ae}{K}_{Bm}} + \frac{{C}_{Am}{C}_{Be}}{{K}_{Am}{K}_{Be}} \right )},\\ \end{eqnarray*}

where *e* and *m* subscripts are for the extra-mitochondrial region and mitochondrial matrix, respectively; A and B are the different species being transported between the extra-mitochondrial region and mitochondrial matrix; *K*’s are the Michaelis–Menten or binding constants of the species for the transporters at the external (e) or internal (m) side; *T_maxf_*’s are the maximal forward transport rates. Species are assumed to be rapidly equilibrating between the extra-mitochondrial and IMS regions, and therefore their concentrations are assumed to be equal in these 2 regions.

#### Model Governing ODEs

The model includes 37 state variables in different regions representing TCA cycle metabolites, adenine nucleotides, inorganic phosphate, redox variables, oxygen, and membrane potential. The dynamics of the state variables in a region is governed by ODEs-based mass balance of the state variables in that region:


(12) \begin{eqnarray*} \ \ {V}_m\frac{{d{C}_{m,j}}}{{dt}} = \sum\limits_k {{\beta }_{m,k,j}{J}_{m,k,j}} + \sum\limits_k {{J}_{e \leftrightarrow m,k,j}}, \end{eqnarray*}

(13) \begin{eqnarray*} {V}_i\frac{{d{C}_{i,j}}}{{dt}} = \sum\limits_k {{\beta }_{i,k,j}{J}_{i,k,j}}, \end{eqnarray*}

(14) \begin{eqnarray*} {V}_e\frac{{d{C}_{e,j}}}{{dt}} = - \sum\limits_k {{J}_{e \leftrightarrow m,k,j}}, \end{eqnarray*}

where subscripts *m, i*, and *e* denote the mitochondrial matrix, IMS, and extra-mitochondrial (buffer) regions; ${C}_{x,j}$ is the concentration of *j*^th^ species in the region *x; V_x_* is the volume of the region *x; β_x_,_k_,_j_* is the stoichiometric coefficient of *j*^th^ species in *k*^th^ reaction in the region *x* (positive or negative depending on the species is a reactant or a product); ${J}_{x,,k,j}$ is the *k*^th^ reaction flux involving *j^th^* species in the region *x*; and ${J}_{e \leftrightarrow m,k,j}$ is the *k*^th^ transporter flux involving *j*^th^ species between the regions *e* and *m*. Detailed mass balance equations and the associated initial conditions for metabolites are included in the Supplementary Material S4.

### Modeling SUC-Pathway Mediated Regulations

To describe potential differences between mitochondrial metabolism in the heart and kidney cortex and OM, we added specific regulations to several metabolic enzyme and metabolite transporter models. These regulations helped us to uncover the hidden differences in underlying mechanisms of substrate-dependent mitochondrial respiration and bioenergetics between the heart and kidney cortex and OM mitochondria, as observed by Tomar et al.^1^

The dicarboxylate carrier (DCC; gene name SLC25A10) transports both malate (MAL) and succinate (SUC) in exchange for inorganic phosphate (Pi) across the IMM, as studied extensively by Palmieri and coworkers.^38–43^ Accordingly, both MAL and SUC compete for the binding and transport by the DCC, and hence, one can inhibit the transport of the other. Different kinetic models of this transporter have been developed by Bazil et al.^31^ and Zhang et al.^28^ accounting for the competitive binding and inhibition by each other during their transport via the DCC. We have distinguished the transport of MAL and SUC by the DCC as DCCM and DCCS, respectively, having similar kinetic mechanisms (ie, inhibition of the transport of cytosolic MAL by mitochondrial SUC and inhibition of the transport of cytosolic SUC by mitochondrial MAL). However, the regulation of these 2 transport processes (DCCM and DCCS) and the associated kinetic parameters have not been evaluated and compared in different tissues, for example, heart and kidney. Given the different respiratory responses of isolated mitochondria from the heart and kidney cortex and OM to SUC substrate,^1^ we hypothesized that SUC and MAL transport and their oxidations must be differentially regulated in the heart and kidney cortex and OM.

Similarly, it is well-known that oxaloacetate (OXA) is a potent inhibitor and malate (MAL) is a potent activator of the succinate dehydrogenase (SDH) reaction.^44^ In addition, in several recent studies including that of Fink and coworkers,^45,46^ the inhibitory role of OXA on SUC-driven mitochondrial respiration/OxPhos has been firmly established. However, the differential SUC-driven respiration/OxPhos inhibition by OXA and activation by MAL in the mitochondria from different tissues such as the heart and kidney cortex and OM have not been well established or compared. Hence, given different respiratory responses of isolated mitochondrial from the heart and kidney cortex and OM to SUC substrate,^1^ we hypothesized that SUC oxidation by SDH/complex II must be differentially regulated in the heart and kidney cortex and OM by OXA and MAL.

Therefore, to simulate the observed differences in mitochondrial metabolic responses between SUC vs. SUC + ROT in the 3 tissues,^1^ we modified SUC-dependent transporters and enzymes in the mitochondria including DCCS, DCCM, and SDH, as described below.

The SUC-Pi antiporter (DCCS) flux expression in eqn (15) was modified to account for the inhibitory effect of mitochondrial MAL on the DCCS transporter by scaling the intrinsic SUC binding constant for DCCS, *K*_SUC_, as follows:


(15) \begin{eqnarray*} {J}_{DCCS} = \frac{{{T}_{\max f,DCCS}\left( {\frac{{{C}_{SUCe}{C}_{Pim} - {C}_{SUCm}{C}_{Pie}}}{{{{K^{\prime}}_{SUC}}{K}_{Pi}}}} \right)}}{{\left( {1 + \frac{{{C}_{SUCe}}}{{{{K^{\prime}}_{SUC}}}} + \frac{{{C}_{Pie}}}{{{K}_{Pi}}} + \frac{{{C}_{SUCm}}}{{{{K^{\prime}}_{SUC}}}} + \frac{{{C}_{Pim}}}{{{K}_{Pi}}} + \frac{{{C}_{SUCe}{C}_{Pim}}}{{{{K^{\prime}}_{SUC}}{K}_{Pi}}} + \frac{{{C}_{SUCm}{C}_{Pie}}}{{{{K^{\prime}}_{SUC}}{K}_{Pi}}}} \right)}}, \end{eqnarray*}

(16) \begin{eqnarray*} {K^{\prime}_{SUC}} = {K}_{SUC}\left( {1 + \frac{{{C}_{MALm}}}{{{K}_{MAL}}}} \right), \end{eqnarray*}

where *C_MALm_* is the MAL concentration in the mitochondrial matrix; *K*_MAL_ is the regulatory MAL binding constant for DCCS to compete for SUC binding; ${K^{\prime}_{SUC}}$ is the apparent SUC binding constant accounting for the inhibitory effect of MAL accumulation in the mitochondrial matrix on DCCS (MAL is assumed to inhibit the influx of SUC into the mitochondrial matrix). Hence, an increase in MAL concentration in the mitochondrial matrix inhibits SUC binding to DCCS, which in turn inhibits SUC influx into the mitochondrial matrix.

Similarly, we modified the MAL-Pi antiporter (DCCM) flux expression in eqn (17) to account for the inhibitory effect of mitochondrial SUC on the DCCM transporter by modifying the intrinsic MAL binding constant for DCCM, *K*_MAL_, as follows:


(17) \begin{eqnarray*} {J}_{DCCM} = \frac{{{T}_{\max f,DCCM}\left( {\frac{{{C}_{MALe}{C}_{Pim} - {C}_{MALm}{C}_{Pie}}}{{{{K^{\prime}}_{MAL}}{K}_{Pi}}}} \right)}}{{\left( {1 + \frac{{{C}_{MALe}}}{{{{K^{\prime}}_{MAL}}}} + \frac{{{C}_{Pie}}}{{{K}_{Pi}}} + \frac{{{C}_{MALm}}}{{{{K^{\prime}}_{MAL}}}} + \frac{{{C}_{Pim}}}{{{K}_{Pi}}} + \frac{{{C}_{MALe}{C}_{Pim}}}{{{{K^{\prime}}_{MAL}}{K}_{Pi}}} + \frac{{{C}_{MALm}{C}_{Pie}}}{{{{K^{\prime}}_{MAL}}{K}_{Pi}}}} \right)}},\\ \end{eqnarray*}

(18) \begin{eqnarray*} {K^{\prime}_{MAL}} = {K}_{MAL}\left( {1 + \frac{{{C}_{SUCm}}}{{{K}_{SUC}}}} \right), \end{eqnarray*}

where *C_SUCm_* is the SUC concentration in the mitochondrial matrix region; *K*_SUC_ is the regulatory SUC binding constant for DCCM to compete for MAL binding; ${K^{\prime}_{MAL}}$is the apparent MAL binding constant after the inhibitory effect of SUC accumulation in the mitochondrial matrix on DCCM accounted for. Hence, as the SUC concentration in the mitochondrial matrix increases, MAL binding to DCCM is inhibited and therefore MAL outflux to the buffer region is inhibited (reverse mode of DCCM). The postulated mechanism of DCCM inhibition by SUC is via its competition with MAL for binding to DCCM.

To account for the inhibitory effect of OXA and stimulatory effect of MAL on the SDH reaction flux, we modified the intrinsic SUC binding constant for the SDH enzyme, *K*_SUC_, as follows:


(19) \begin{eqnarray*} J_{SDH} = \frac{{\frac{V_{maxf,SDH}}{K^{\prime\prime}_{SUCm}K_{UQm}}\left(C_{SUCm}C_{UQm} - \frac{C_{FUMm}C_{UQH2m}}{K^{\prime}_{eq,SDH}} \right )}}{\left (1 + \frac{C_{SUCm}}{K^{\prime\prime}_{{SUC_m}}} + \frac{C_{FUMm}}{K_{FUMm}} \right ){\left (1 + \frac{C_{UQm}}{K_{{UQm}}} + \frac{C_{UQH2m}}{K_{UQH2m}} \right )}}, \end{eqnarray*}

(20) \begin{eqnarray*} {K^{\prime\prime}_{SUC}} = {{{{K^{\prime}}_{SUC}}} \mathord{\left/ {\vphantom {{{{K^{\prime}}_{SUC}}} {\left( {1 + \frac{{{C}_{MALm}}}{{{K}_{MAL}}}} \right)}}} \right. } {\left( {1 + \frac{{{C}_{MALm}}}{{{K}_{MAL}}}} \right)}}, \end{eqnarray*}

(21) \begin{eqnarray*} {K^{\prime}_{SUC}} = {K}_{SUC}\left( {1 + \frac{{{C}_{OXAm}}}{{{K}_{OXA}}}} \right), \end{eqnarray*}

where *C_OXAm_* is the OXA concentration in the mitochondrial matrix; *K*_OXA_ is the regulatory OXA binding constant for SDH; *C_MALm_* is the MAL concentration in the mitochondrial matrix; *K*_MAL_ is the regulatory MAL binding constant for SDH; ${K^{\prime}_{SUC}}$ is the apparent SUC binding constant after the inhibitory effect of OXA accumulation in the mitochondrial matrix on SDH is accounted for; ${K^{\prime\prime}_{SUC}}$ is the apparent SUC binding constant after the stimulatory effect of MAL accumulation in the mitochondrial matrix on SDH is accounted for. As a result of SUC addition, OXA is produced at a high rate and accumulates in the mitochondrial matrix, which then inhibits SDH by reducing SUC binding affinity (ie, increasing SUC binding constant) for SDH. This results in an increase in SUC concentration in the mitochondrial matrix, which in turn inhibits DCCM stalling outflux of MAL. Inhibition of DCCM leads to MAL accumulation in the mitochondrial matrix, which in turn stimulates SDH by increasing SUC binding affinity (ie, decreasing SUC binding constant). Accumulation of MAL in the mitochondrial matrix also inhibits DCCS stalling SUC influx. As a result of these regulations, SUC concentration in the mitochondrial matrix is reduced, which leads to reversing DCCM inhibition, increasing MAL outflux, and decreasing MAL concentration in the mitochondrial matrix. Reduced MAL concentration in the mitochondrial matrix also reverses DCCS inhibition and enhances SUC influx.

### Parameter Estimation

Intrinsic model parameters such as Michaelis–Menten constants (*K*_m_’s) characterizing the binding of metabolites (reactants and products) to the enzymes and transporters are set to values from our previous studies based on isolated enzymes and transporters kinetics.^28,31,35^ The assumption is that differences in the *K*_m_ values between different tissues are negligible. The tissue-specific unknown extrinsic model parameters such as maximum reaction velocities (*V*_max_’s and *T*_max_’s) of different enzymatic and transporter reactions were estimated as described below.

The extrinsic model parameters *V*_max_’s and *T*_max_’s are tissue-specific, because of differential expressions of enzymes and transporters and their catalytic activities and regulations in different tissues to perform distinct metabolic functions. Therefore, these parameters were estimated separately for each tissue based on tissue-specific experimental data, including mitochondrial O_2_ consumption rate (peak OCR or *J_O2_* flux) with different metabolic substrates at different respiratory states by ADP. This was achieved by minimizing the objective function defined below using the optimization functions “*ga*” (genetic algorithm) and/or “*fmincon*” (constrained minimization algorithm) in MATLAB (Mathworks Inc.). The objective function *E*(*P*) is defined as the normalized sum of squared errors (SSE) between model simulations and experimental data:


(22) \begin{eqnarray*} E(P) = {\sum\limits_{j = 1}^M {\frac{1}{{{N}_j}}\mathop \sum \limits_{i = 1}^{{N}_j} \left( {\frac{{{x}_{i,j} - {X}_{i,j}(P)}}{{\max ({x}_{i,j})}}} \right)} }^2, \end{eqnarray*}

where ${X}_{i,j}$ and ${x}_{i,j}$ are the model solutions (peak OCR or *J_O_*_2_ flux as functions of added ADP concentration, which depend on the parameter values *P*) and the corresponding experimental data at the *i*^th^ data point in the *j*^th^ data set, respectively; *N_j_* is the number of data points in a given data set (ADP variation); and *M* is the number of data sets (different substrates) used for the parameter estimation. The estimated *V*_max_ and *T*_max_ values for the heart and kidney cortex and OM mitochondria are presented in Table 2 and in Figure S2 of the Supplementary Material S5. The general model parameters including temperature, buffer volume, mitochondrial matrix volume, and IMS volume were fixed at 37°C, 2 mL (respirometry) or 1 mL (spectrofluorometry), 1 μL/mg protein, and 0.1 μL/mg protein, respectively, based on experimental setup and literature (Table 1).

**Table 2.** Estimated values of tissue-specific *V*_max_ and *T*_max_ parameters (unit: nmol/min/mg mitochondrial protein at 37°C) and computed normalized sensitivity coefficients.

Parameters	Estimated Values (Heart)	Normal Sensitivity Coefficient	Parameter Values (Cortex)	Normal Sensitivity Coefficient	Parameter Values (OM)	Normal Sensitivity Coefficient
*V_maxf__, PDH_*	1.016 × 10^5^	−0.0016	0.575 × 10^5^	−0.0022	0.237 × 10^5^	−0.0004
*V_maxf__, CITS_*	0.447 × 10^5^	−0.0120	0.399 × 10^5^	−0.0813	0.147 × 10^5^	−0.0855
*V_maxf__, ICDH_*	0.240 × 10^5^	−0.0041	0.318 × 10^5^	−0.0081	0.143 × 10^5^	−0.0045
*V_maxf__, AKGDH_*	0.276 × 10^5^	0.0772	0.0006 × 10^5^	−0.0660	0.0004 × 10^5^	0.0844
*V_maxf__, SCAS_*	0.488 × 10^5^	0.0132	0.320 × 10^5^	0.0248	0.173 × 10^5^	−0.0116
*V_maxf__, NDK_*	0.332 × 10^5^	0.0019	0.344 × 10^5^	0.0179	0.183 × 10^5^	−0.0083
*V_maxf__, FH_*	0.361 × 10^5^	0.0006	0.333 × 10^5^	−0.0083	0.163 × 10^5^	0.0004
*V_maxf__, MDH_*	0.454 × 10^5^	0.0052	0.214 × 10^5^	−0.2065	0.110 × 10^5^	−0.1238
*V_maxf__, GOT_*	0.251 × 10^5^	1.0255	0.086 × 10^5^	−0.1324	0.017 × 10^5^	0.2330
*V_maxf__, CI_*	0.766 × 10^5^	0.0127	0.348 × 10^5^	0.0280	0.184 × 10^5^	0.0230
*V_maxf__, CII_*	0.094 × 10^5^	0.3050	0.218 × 10^5^	−0.8870	0.002 × 10^5^	−2.5551
*V_maxf__, CIII_*	0.066 × 10^5^	1.2662	0.003 × 10^5^	−3.6071	0.013 × 10^5^	−1.5650
*V_maxf__, CIV_*	0.021 × 10^5^	−0.0524	0.001 × 10^5^	−0.1835	0.001 × 10^5^	−0.4017
*V_maxf__, CV_*	0.241 × 10^5^	0.0463	0.004 × 10^5^	−0.7256	0.011 × 10^5^	0.0109
*T_maxf__, PYRH_*	0.875 × 10^5^	−0.0039	0.521 × 10^5^	0.0002	0.217 × 10^5^	0.0019
*T_maxf__, GLUH_*	0.377 × 10^5^	−0.0012	0.317 × 10^5^	0.0076	0.169 × 10^5^	−0.0007
*T_maxf__, DCCS_*	0.232 × 10^5^	−0.2309	0.039 × 10^5^	0.2168	0.192 × 10^5^	−0.0072
*T_maxf__, DCCM_*	0.654 × 10^5^	−0.4548	0.031 × 10^5^	−0.0527	0.160 × 10^5^	−0.0089
*T_maxf__, TCC_*	0.826 × 10^5^	−0.0074	0.381 × 10^5^	−0.0022	0.195 × 10^5^	0.0010
*T_maxf__, OME_*	0.407 × 10^5^	−0.0450	0.0003 × 10^5^	−0.2027	0.226 × 10^5^	−0.0005
*T_maxf__, GAE_*	0.160 × 10^5^	0.0030	0.253 × 10^5^	0.0075	0.138 × 10^5^	−0.0001
*T_maxf__, ANT_*	0.028 × 10^5^	4.1144	0.009 × 10^5^	−2.0595	0.002 × 10^5^	−1.5695
*T_maxf__, PIC_*	0.815 × 10^5^	0.3768	0.086 × 10^5^	−0.1678	0.083 × 10^5^	−0.1332
*T_maxf__, HLEAK_*	0.937	−0.1036	0.507	−0.6125	0.266	−0.2911

### Parameter Sensitivity Analysis and Correlation Coefficient Matrix

Model parameter sensitivity analysis was performed in 2 ways after parameter estimation. First, the variation in *E*/*E*_0_ as a function of *P_j_*/*P_j_*,_0_ was characterized for each parameter *P_j_* in the range 0.5*P_j_*,_0_ ≤ *P_j_* ≤ 1.5*P_j_*,_0_, where *P_j_*,_0_ is the estimated optimal value of *P_j_* and *E*_0_ is the corresponding optimal value of *E*. Second, the normalized sensitivity coefficients for the optimal parameter estimates were calculated using the following equation:


(23) \begin{eqnarray*} {S}_{{P}_j} &=& \frac{{{P}_{j,0}}}{{{E}_0}}{\left( {\frac{{\partial E}}{{\partial {P}_j}}} \right)}_{{P}_{j,0}} = \frac{{{P}_{j,0}}}{{{E}_0}}\left( {\frac{{E\left( {{P}_{j,0} + \Delta {P}_{j,0}} \right) - E\left( {{P}_{j,0} - \Delta {P}_{j,0}} \right)}}{{2\Delta {P}_{j,0}}}} \right)\\ &&\quad = \frac{{E\left( {{P}_{j,0} + 0.01{P}_{j,0}} \right) - E\left( {{P}_{j,0} - 0.01{P}_{j,0}} \right)}}{{0.02{E}_0}}. \end{eqnarray*}

A central difference method with 1% change in *P_j_*,_0_ is used to accurately compute the normalized parameter sensitivity coefficients. A relatively high sensitivity value indicates that changing a given parameter value would result in a significant change in the model simulations and the SSE objective function (*E*). The sensitivity analysis results for the heart and kidney cortex and OM mitochondria are presented in Table 2 and Figures S3 and S4 in the Supplementary Material S5.

The correlation coefficients ($C{C}_{ij}$) between the model parameters that best fit the model solutions to the experimental data were obtained from eqn 24:


(24) \begin{eqnarray*} {CC}_{ij} = \frac{{HM}_{ij}}{{\sqrt {{HM}_{ii} \times {HM}_{jj}}}},\,\,\,\,\,\,\,\,\,\,\,\,\,\,{\rm{for}}\,i,{\rm{ }}j = 1, \ldots ,Np, \end{eqnarray*}

where *Np* is the number of model parameters and *HM* is the inverse of the product of the Jacobian matrix (*JM*) of the model solution and its transpose (*JM’*). The model solution for mitochondrial OCR (*J*_O2_ flux) as a function of added ADP concentrations for 5 different substrates was fitted to corresponding experimental data. Thus,


(25) \begin{eqnarray*} HM = inv(JM \times JM^{\prime}), \end{eqnarray*}

where


(26) \begin{eqnarray*} J{M}_{i,j} &=& {\left( {\frac{{\partial {J}_{{O}_2,i}}}{{\partial {P}_j}}} \right)}_{{P}_{j,0}} = \frac{{{J}_{{O}_2,i}\left( {{P}_{j,0} + \Delta {P}_{j,0}} \right) - {J}_{{O}_2,i}\left( {{P}_{j,0} - \Delta {P}_{j,0}} \right)}}{{2\Delta {P}_{j,0}}}\\ &&\quad = \frac{{{J}_{{O}_2,i}\left( {{P}_{j,0} + 0.01{P}_{j,0}} \right) - {J}_{{O}_2,i}\left( {{P}_{j,0} - 0.01{P}_{j,0}} \right)}}{{0.02{P}_{j,0}}}, \end{eqnarray*}

A high correlation coefficient between 2 estimated model parameter values indicates their dependency on each other, suggesting the nonidentifiability and nonestimability of the 2 model parameters. The correlation coefficient matrices for the heart and kidney cortex and OM mitochondria are presented in Figure S5 in the Supplementary Material S5.

## Results

The computational models of the heart and kidney cortex and OM mitochondrial respiration and bioenergetics were developed and parameterized by individually fitting them to the OCR data obtained from isolated mitochondria oxidizing 5 different metabolic substrate combinations, followed by sequential additions of increasing ADP concentrations. These parameterized models were then validated by predicting the OCR and ${\rm{\Delta }}{{\rm{\Psi }}}_m$ data in the presence of the same 5 substrate combinations followed by single dose of ADP addition. Using these validated models, key mitochondrial bioenergetic state variables and emergent metabolic system properties, such as NADH ratio, UQH_2_ ratio, CytCred ratio, ${\rm{\Delta }}{{\rm{\Psi }}}_m$, and respiratory control index (RCI; state 3 OCR/state 2 OCR) were predicted under physiological and pathological conditions. In particular, the mitochondrial proton leak (UCP2) activity was increased to simulate a pathological condition induced by mitochondrial uncoupling of OxPhos in each of the 3 tissues, and to predict alterations of emergent metabolic system properties. The validated models were also used to develop hypotheses that may explain the differences observed in the oxidation of SUC vs. SUC + ROT in the 3 tissues. The heart mitochondria showed relatively large differences in metabolite concentrations and metabolic fluxes in the presence of SUC vs. SUC + ROT while the differences were relatively small in the kidney OM mitochondria and non in the kidney cortex mitochondria.

### Model Fittings and Parametrization Using Mitochondrial Respiration Data With Sequential and Incremental ADP Additions

**Figure 2.**
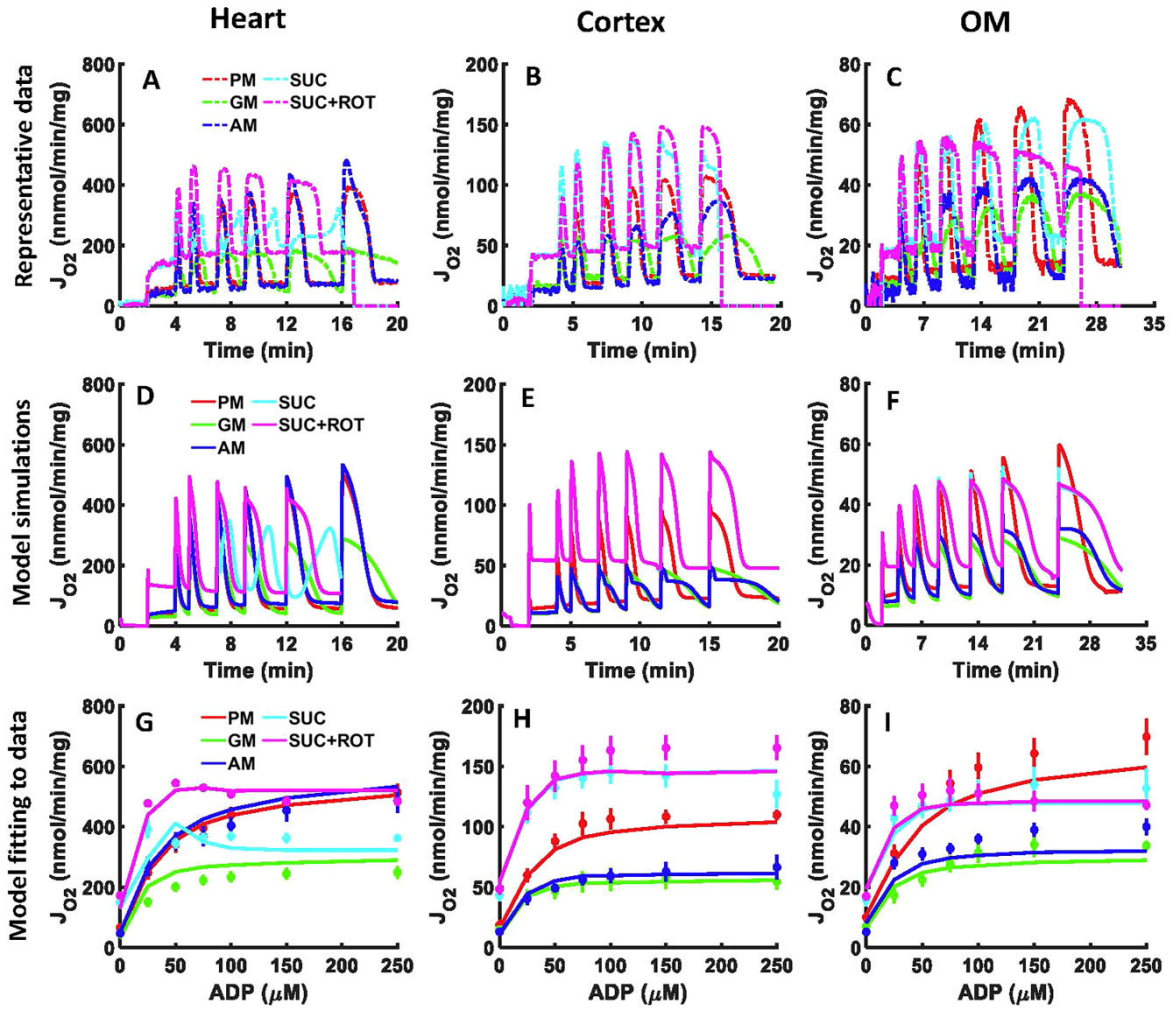
Model simulations and fittings to the OCR experimental data of isolated mitochondria from the heart and kidney cortex and OM mitochondria in response to sequential and incremental ADP additions. Comparison of the time courses of mitochondrial OCR representative data (A–C) with the corresponding model simulations (D–F) in the heart, kidney cortex, and kidney OM in response to sequential ADP additions of 25, 50, 75, 100, 150, and 250 µm. Model fitting to the peaks of the OCR data after each ADP addition (G–I). Five different substrate combinations are used including PM (5:2.5 m m), AM (5:2.5 m m), GM (5:2.5 m m), SUC (10 m m), and SUC + ROT (10 m m + 0.5 µm). The representative data are shown with dashes, the OCR peaks data are shown with symbols, and the model simulations are shown with solid lines. Model simulations for SUC and SUC + ROT overlap in the kidney cortex and OM mitochondria (pink and cyan lines).

Figure 2A–C depict the time courses of measured OCR (*J*_O2_ flux) in isolated mitochondria from the SD rat heart and kidney cortex and OM, as reported from our laboratory by Tomar et al.,^1^ based on the experimental protocol described in Figure S1A. In this protocol, increasing concentrations of ADP were sequentially added to isolated mitochondria in the presence of 5 different substrate combinations. Across substrates, the heart mitochondria had significantly higher OCRs than the kidney cortex and OM mitochondria, and the kidney cortex mitochondria had significantly higher OCRs than the kidney OM mitochondria. Furthermore, there were distinct differences in the OCR profiles for different substrates in each tissue and between tissues. For instance, the dynamics of OCR while utilizing SUC vs. SUC + ROT were distinctly different in the heart mitochondria but were similar in the kidney cortex and OM mitochondria. Additionally, for all 5 substrate combinations and for all 3 tissues studied, the state 3 OCR increased with increasing ADP concentrations, reaching maximal values at saturating ADP concentrations. These maximal OCR values also differed significantly between different substrates and different tissues. All descriptions of significance are based on the statistical analyses in the studies of Tomar et al.^1^

The mitochondrial models were tailored to capture the distinctive features of the OCR data for various substrates. To achieve this, for each tissue, the solution of the corresponding model equation (ie, *J*_O2_ or *J*_CIV_ flux) was concurrently fitted to the average of state 2 OCR data after each substrate addition and the peaks of state 3 OCR data after each ADP addition for each substrate (Figure 2G–I). By using the values of the model parameters estimated from the OCR data in Figure 2G–I, the models were then able to simulate the time courses of OCR data in Figure 2A–C with corresponding state 3 durations in the heart and kidney cortex and OM mitochondria as well as the substrate-dependent differences in the 3 tissues (Figure 2D–F). In line with the experimental findings, the model simulations of OCR were lowest for GM and SUC in the heart mitochondria and GM and AM in the kidney cortex and OM mitochondria, and highest while oxidizing SUC + ROT in the heart mitochondria, SUC ± ROT in the kidney cortex mitochondria, and SUC ± ROT and PM in the kidney OM mitochondria. Thus, in all 3 tissues, GM had the lowest respiration while SUC + ROT had the highest respiration. Moreover, the model was able to predict the distinct responses to the addition of ADP between PM and SUC(±ROT) in the kidney cortex and OM mitochondria, as shown in Figure 2E, F, H, I.

Based on the optimal estimates of the extrinsic model parameter values (Table 2 and Figure S2), it was observed that the *V*_max_ and *T*_max_ values for the heart mitochondria were consistently higher than those for the kidney cortex (>2 times) and OM mitochondria (>4 times), which is consistent with the differences in the measured OCRs in these tissues. This suggests that differential expressions of the enzymes and transporters and their catalytic activities and regulations in different tissues are required for distinct metabolic functions. For a specific tissue, the estimated *V*_max_ and *T*_max_ values were also widely variable among themselves, indicating differential expressions, activities, and regulations in the tissue to optimally perform their individual functions. Interestingly, except for complex I (CI), for other membrane potential (ΔΨ_m_)-dependent transporters and pumps (eg, CIII–CV, ATP/ADP exchange, and H^+^ leak), the estimates of *V*_max_’s and *T*_max_’s were small, indicating that the high activity of CI was a consequence of its high abundance. Furthermore, the activity of AKGDH was found to be several orders of magnitude lower in the kidney cortex and OM mitochondria than the heart mitochondria, consistent with the observed lower OCR with the AKG substrate in the kidney cortex and OM mitochondria than the heart mitochondria. These results suggest that the activities of various enzymes and transporters are critical determinants of mitochondrial respiration and bioenergetics in different tissues.

The parameter sensitivity analyses (Figures S3–S4) and the computed parameter correlation coefficient matrices (Figure S5A–C) revealed that most of the *V*_max_ and *T*_max_ parameters were robustly estimated for the heart and kidney cortex and OM mitochondrial models. As depicted in Figure S3, most of the normalized sensitivity coefficients for the SSE objective function, *E*(*P*), computed using the optimal parameter estimates *P_0_* (eqn 23), were small and on the same order of magnitude for all 3 tissues. This was further verified in the parameter sensitivity plots in Figure S4 in which *E/E_0_* did not vary appreciably from one in the neighborhood of *P_j_/P_j_*,_0_ = 1 for most parameters (*P_j_*) and for all 3 tissues. Small changes in some parameters (*P_j_/P_j_*,_0_), such as the activities of the ETC complexes and phosphate transporters, resulted in prominent changes in *E/E*_0_ showing high sensitivity of the model solutions for the measured variable (*J*_O2_) to those parameters. Both Figures S3 and S4 show that in the heart model, the SSE objective function, *E*(*P*), was highly sensitive to the activities of DCCS, DCCM, ANT GOT (glutamate-oxaloacetate transaminase), CII, and CIII, while in the kidney cortex and OM models, the SSE objective function was most sensitive to the activities of ANT, H^+^ leak, CII, CIII, and CV. This is conceivable as these transporters and enzymes are directly associated with the measured variables (*J*_O2_) and perturbations (ie, substrate transport and ATP/ADP exchange). There were also several parameters for which the model solutions (*J*_O2_) were not very sensitive to large changes in the parameter values (0.5 *P_j_*,_0_ ≤ *P_j_* ≤ 1.5 *P_j_*,_0_), suggesting that any values for those parameters within the specified range provide as good a fit to the data as the optimal fit.

The correlation coefficient matrices in Figure S5A–C show the direction and amplitude of correlations between every 2 parameters in each of the 3 models. Usually, a small correlation coefficient (eg, |CC| < 0.8) between 2 parameters indicates a weak dependency between those parameters, and a high confidence in the estimability of those parameters. This was noted for many pairs of parameters in all 3 models. On the other hand, relatively high correlation coefficients (ie, |CC| > 0.8) were also obtained between few of the parameters. For example, in the heart mitochondrial model, the highest correlation coefficients (ie, |CC| > 0.9) were obtained between H^+^ leak and CII, CIII, and CIV activities, and between malate dehydrogenase (MDH) and citrate synthase (CITS) activities. Similarly, high correlation coefficients were obtained between few of the estimated parameters in the other 2 models. For example, in the kidney OM mitochondrial model, the activity of tricarboxylic carrier (TCC) was highly correlated with the activities of pyruvate dehydrogenase (PDH) and pyruvate-H^+^ cotransporter (PYRH). In addition, the activity of PYRH was negatively correlated with the activity of isocitrate dehydrogenase (IDH) and positively correlated with the activities of PDH and glutamate-aspartate exchanger (GAE); the activity of GAE was negatively correlated with the activities of IDH and glutamate-H^+^ cotransporter (GLUH); the activity of CII was negatively correlated with the activity of CIII.

### Model Validation and Corroboration of Mitochondrial Respiration and Membrane Potential With the Addition of a Single Dose of ADP

**Figure 3.**
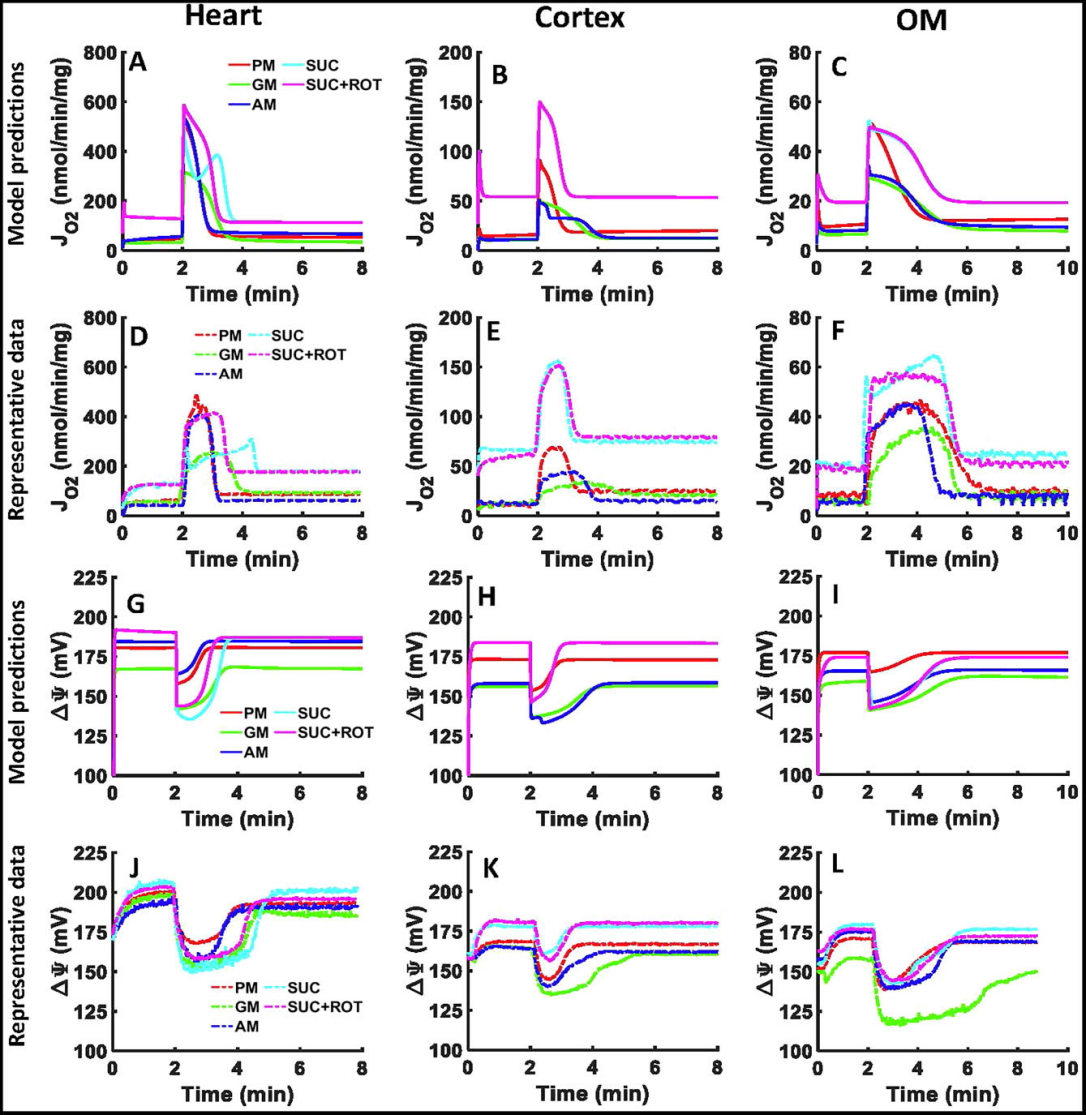
Model validation through comparison of model simulations with mitochondrial OCR and ${{\bf \Delta }}{{{\bf \Psi }}}_{\boldsymbol{m}}$ experimental data from the heart and kidney cortex and OM. Comparison of model simulations of mitochondrial OCR time courses (A–C) in the presence of 5 different substrate combinations with addition of 200 µm ADP to the heart mitochondria and 100 µm ADP to the kidney cortex and OM mitochondria with the corresponding representative OCR experimental data (D–F). Comparison of model simulation of ${\rm{\Delta }}{{\rm{\Psi }}}_m$ time courses (G–I) in the presence of 5 different substrate combinations with addition of 200 µm ADP to the heart mitochondria and 100 µm ADP to the kidney cortex and OM mitochondria with the corresponding representative ${\rm{\Delta }}{{\rm{\Psi }}}_m$ experimental data (J–L). The 5 different substrate combinations include PM (5:2.5 m m), AM (5:2.5 m m), GM (5:2.5 m m), SUC (10 m m), and SUC + ROT (10 mM + 0.5 µm). The representative data are shown with dashes and the model simulations are shown with solid lines. For clarity, the model simulations and experimental data are shown from the time of substrate addition. Model simulations for SUC and SUC + ROT overlap in the kidney cortex and OM mitochondria (pink and cyan lines).

The parameterized models were validated by predicting the OCR and ${\rm{\Delta }}{{\rm{\Psi }}}_{\rm{m}}$ dynamics and comparing them with the corresponding experimental data^1^ collected from isolated heart and kidney cortex and OM mitochondria (Figure 3). Those datasets were not used for the parameterization of the models. As described in Figure S1B, the heart mitochondria were stimulated with 200 µm ADP and the kidney cortex and OM mitochondria were stimulated with 100 µm ADP. All were energized in the presence of 5 different substrate combinations. The representative experimental data (Figure 3D–F) showed that SUC + ROT had the highest and GM had the lowest OCR in all tissues, consistent with results from the sequential ADP addition protocol (Figure 2A–C). In the heart mitochondria, PM and SUC + ROT had the highest OCR, and in the kidney cortex and OM mitochondria, SUC and SUC + ROT had the highest OCR (Figure 3D–F). Our models were able to predict the similar time courses of OCR with matching state 3 durations for different substrates in the 3 tissues (Figure 3A–C). The absolute OCRs were slightly higher after a single ADP addition compared to ADP addition of the same dose in the sequential ADP addition protocol. As per the model simulations of ${\rm{\Delta }}{{\rm{\Psi }}}_{\rm{m}}$, in the heart mitochondria, SUC and GM had the lowest ${\rm{\Delta }}{{\rm{\Psi }}}_{\rm{m}}$, while PM and AM had the highest ${\rm{\Delta }}{{\rm{\Psi }}}_{\rm{m}}$ (Figure 3J–L). In the kidney cortex and OM mitochondria, GM had the lowest ${\rm{\Delta }}{{\rm{\Psi }}}_{\rm{m}}$ and SUC ± ROT had the highest ${\rm{\Delta }}{{\rm{\Psi }}}_{\rm{m}}$ (Figure 3J–L). Our models were able to predict the similar time courses of ${\rm{\Delta }}{{\rm{\Psi }}}_{\rm{m}}$ with matching state 3 durations in addition to closely predicting the substrate-dependent differences in ${\rm{\Delta }}{{\rm{\Psi }}}_{\rm{m}}$ dynamics between the 3 tissues (Figure 3G–I). Despite noticeable differences in the OCR dynamics during state 3 respiration, SUC vs. SUC + ROT had the similar ${\rm{\Delta }}{{\rm{\Psi }}}_{\rm{m}}$ dynamics from all 3 tissues.

### Model Predictions of Mitochondrial Bioenergetics Under Sequential and Incremental ADP Additions (Physiological Perturbations)

The validated models were used to simulate the time courses of different metabolic fluxes (Figure S6A–F) and metabolite concentrations (Figure S7A–F) in isolated heart and kidney cortex and OM mitochondria with sequential and incremental additions of ADP in the presence of 5 different metabolic substrate combinations. These predictions show how different substrate combinations differentially activated NADH and FADH_2_-linked metabolic pathways including metabolite and phosphate transporters, TCA cycle, ETC, and OxPhos in the mitochondria of the 3 tissues leading to different metabolic fluxes and metabolite concentrations regulating redox states, ΔΨ_m_, and O_2_ consumption during proton leak-mediated respiration (state 2) and ADP concentration-dependent respiration (state 3) and ATP synthesis characterizing OxPhos.

**Figure 4.**
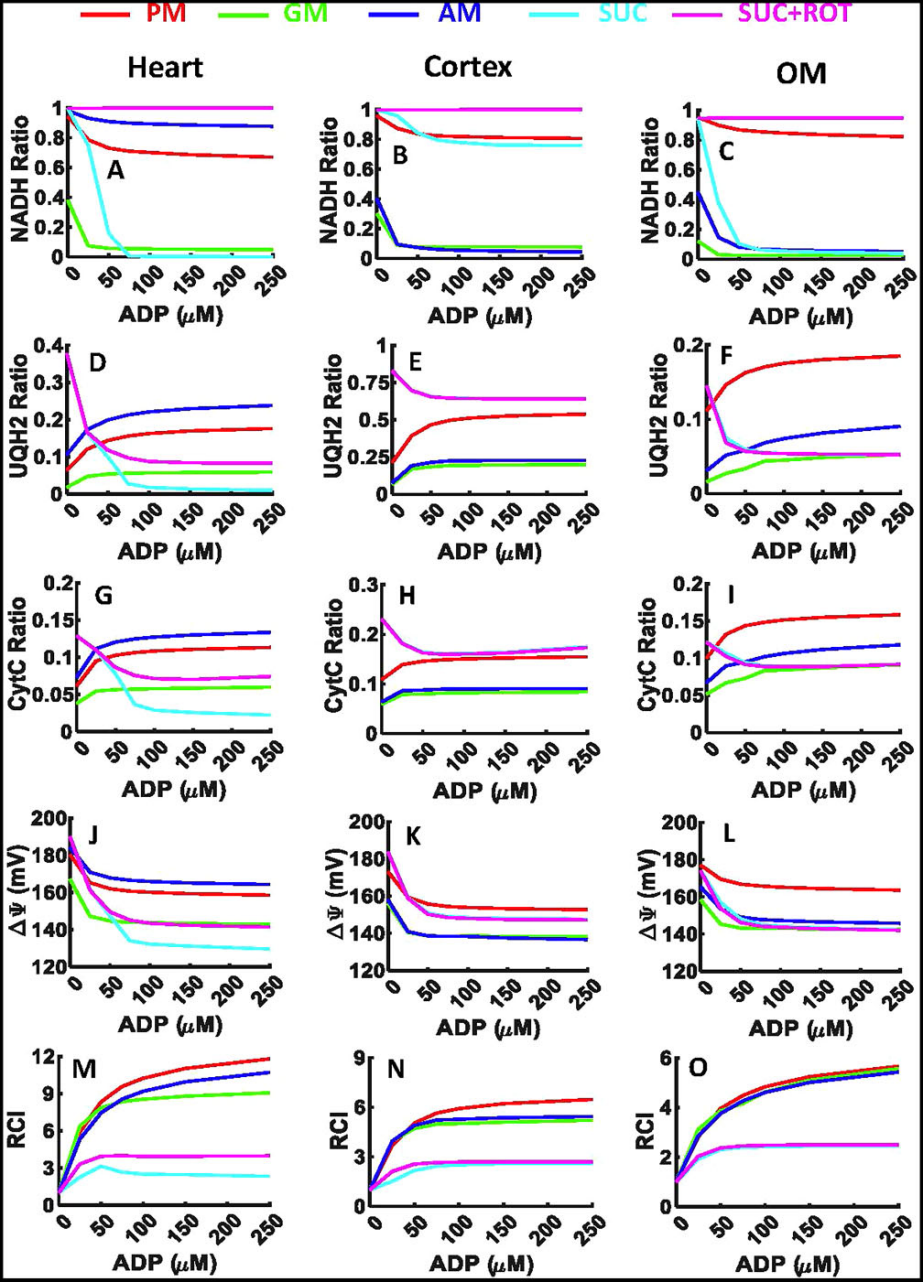
Model predictions of redox ratios, ${{\bf \Delta }}{{{\bf \Psi }}}_{\boldsymbol{m}}$, and RCI in response to sequential and increasing ADP additions to the heart and kidney cortex and OM mitochondria. Model predictions of mitochondrial (A–C) NADH ratio [C_NADH/_(C_NADH_ + C_NAD_)], (D–F) UQH_2_ ratio [C_UQH2/_(C_UQH2_ + C_UQ_)], (G–I) CytCred ratio [C_CytCred/_(C_CytCred_ + C _CytCoxi_)], (J–L) ${\rm{\Delta }}{{\rm{\Psi }}}_m$, and (M–O) RCI in the presence of 5 different substrate combinations including PM (5:2.5 m m), AM (5:2.5 m m), GM (5:2.5 m m), SUC (10 m m), and SUC + ROT (10 m m + 0.5 µm) in response to increasing ADP additions of 25, 50, 75, 100, 150, and 250 µm in the heart, kidney cortex, and kidney OM mitochondria. Some model simulations for SUC and SUC + ROT overlap in the kidney cortex and OM mitochondria (pink and cyan lines).

The dynamic simulation results from Figures S6 and S7 were used to derive key bioenergetic state variables and physiological emergent metabolic system properties including NADH ratio, UQH_2_ ratio, CytCred ratio, ${\rm{\Delta }}{{\rm{\Psi }}}_m$, and RCI (state 3 *J*_O2_/state 2 *J*_O2_) as functions of added ADP concentrations in isolated heart and kidney cortex and OM mitochondria (Figure 4). The redox ratio is defined as the ratio of the concentration of a reduced metabolite to the total (reduced + oxidized) concentration of that metabolite [eg, NADH ratio is defined as *C*_NADH_/(*C*_NAD_ + *C*_NADH_)]. These results provide a quantitative and mechanistic understanding of how mitochondrial redox and bioenergetic states are differentially regulated in response to sequential and incremental ADP additions in the presence of different substrate combinations, leading to differential OCR and ATP synthesis in a particular tissue. These results show that the redox and bioenergetic states vary distinctly in response to ADP in a tissue-specific and substrate-dependent manner.

The simulation results in Figure 4 showed that the NADH ratio decreased in response to ADP for all substrates in all 3 tissues, while the UQH_2_ and CytCred ratios increased with NADH-linked substrates and decreased with FADH_2_-linked substrates. Although the redox responses were similar across all 3 tissues, the absolute values and relative changes in these ratios varied. The most prominent differences in the redox ratios between tissues occurred in the presence of AM or SUC, where the redox changes were similar to those with PM in the heart mitochondria and to those with GM in the kidney cortex and OM mitochondria. Interestingly, the NAD pool became drastically oxidized in response to ADP in the presence of SUC for both the heart and kidney OM mitochondria while it remained reduced in the mitochondria of the kidney cortex. Similarly, the changes in UQH_2_ and CytCred ratios were similar in the kidney cortex and OM mitochondria but different in the heart mitochondria. These distinct redox responses can be attributed to variations in metabolic fluxes in the 3 tissues, as predicted inFigure S6.

The simulation results from Figure 4A to C showed that the NADH ratio varied among the 3 tissues in the presence of different substrates. In the presence of SUC + ROT, the NADH ratio was ∼100% in all 3 tissues due to inhibition of CI. In the presence of PM, it was >70%, and in the presence of GM, it was <10% in response to ADP in all 3 tissues. In the presence of AM, the NADH ratio was ∼90% in the heart mitochondria but only ∼10% in the kidney cortex and OM mitochondria in response to ADP. Furthermore, in the presence of SUC, after addition of only 75 µm ADP, the NAD pool was fully oxidized (ie, NADH ratio was 0%) in the heart mitochondria, ∼10% oxidized in the kidney cortex mitochondria, and ∼90% oxidized in the kidney OM mitochondria. These distinct NADH redox states are attributed to differential activities (maximum velocities) of the NADH producing TCA cycle enzymes in different tissues (Table 2 and Figure S2) and differential activation of the TCA cycle enzymes with different substrate combinations at saturated concentrations.

Model simulations showed that the UQ pool was less reduced in response to ADP compared to the NAD pool in all 3 tissues. In the heart mitochondria, the UQ pool was reduced to ∼5%, ∼18%, and ∼25% in response to ADP in the presence of GM, PM, and AM, respectively (Figure 4D). The increase in UQ pool reduction at saturated ADP was less than 10% for NADH-linked substrates. In the presence of SUC and SUC + ROT, the UQ pool was oxidized by more than 30%, decreasing from ∼40% (at state 2) to ∼10% and ∼1% (at saturated ADP), respectively (Figure 4D). In the kidney cortex mitochondria, the UQ pool was reduced to ∼25% in the presence of GM and AM, ∼50% in the presence of PM, and ∼70% in the presence of SUC ± ROT in response to ADP (Figure 4E). In the kidney OM mitochondria, the UQ pool was reduced to ∼20% for PM, ∼10% for AM, ∼5% for GM, and ∼5% for SUC ± ROT in response to ADP. In the kidney cortex mitochondria, the UQ pool was more reduced compared to the heart and kidney OM mitochondria (Figure 4D–F). The changes in UQH_2_ oxidation in SUC vs. SUC + ROT in response to ADP were the same in the kidney cortex and OM mitochondria despite their differences in the heart mitochondria (Figure 4D–F). The UQ pool in response to ADP in the presence of SUC was oxidized to ∼1%, ∼70%, and ∼5% in the heart, cortex, and OM mitochondria, respectively (Figure 4D–F).

Model simulations predicted that the CytC pool was less reduced compared to the NAD and UQ pools in response to ADP in all 3 tissues. In the heart mitochondria, the CytC pool was reduced to ∼10% for PM and AM, ∼5% for GM and SUC + ROT, and ∼1% for SUC in response to ADP (Figure 4G). In the kidney cortex mitochondria, the CytC pool was reduced to ∼15% for PM and SUC ± ROT and ∼10% for GM and AM in response to ADP (Figure 4H). In the kidney OM mitochondria, the CytC pool was reduced to ∼15% for PM, ∼10% for AM, and ∼8% for GM and SUC ± ROT in response to ADP (Figure 4I). In all 3 tissues, the CytC pool changed between 5% and 20% (Figure 4G–I). These distinct UQ and CytC redox states are attributed to differential activities of the ETC complexes in different tissues (Table 2 and Figure S2) and differential activation of the ETC complexes with different substrate combinations at saturated concentrations.

In all 3 tissues, ${\rm{\Delta }}{{\rm{\Psi }}}_{\rm{m}}$ decreased after addition of ADP due to OxPhos and pumping of H^+^ from IMS to the mitochondrial matrix (Figure 4J–L). In the heart mitochondria, ${\rm{\Delta }}{{\rm{\Psi }}}_{\rm{m}}$ was ∼170 mV for AM, ∼165 mV for PM, ∼145 mV for GM and SUC + ROT, and ∼130 mV for SUC in state 3 (Figure 4J). In the kidney cortex mitochondria, ${\rm{\Delta }}{{\rm{\Psi }}}_{\rm{m}}$ was ∼160 mV for PM and SUC ± ROT and ∼140 mV for AM and GM (Figure 4K). In the kidney OM mitochondria, ${\rm{\Delta }}{{\rm{\Psi }}}_{\rm{m}}$ was ∼170 mV for PM and ∼140 mV for AM, GM, and SUC ± ROT (Figure 4L). These distinct ${\rm{\Delta }}{{\rm{\Psi }}}_{\rm{m}}$ are attributed to differential activities of the ETC complexes in different tissues (Table 2 and Figure S2) in the presence of different substrate combinations at saturated concentrations, leading to differential redox states and proton pumping, as predicted in Figures S6 and S7.

Model simulations predicted that the RCI values (state 3 *J*_O2_/state 2 *J*_O2_) were highest in the heart mitochondria compared to the kidney cortex and OM mitochondria, which had similar RCIs (Figure 4M–O). In the heart mitochondria, the RCI values were ∼11 for PM, ∼9 for AM, ∼7 for GM, ∼3 for SUC + ROT, and ∼2 for SUC at saturated ADP (Figure 4M). In the kidney cortex mitochondria, the corresponding RCI values were ∼6 for PM, ∼5 for AM and GM, and ∼3 for SUC ± ROT (Figure 4N), while in the kidney OM mitochondria, the RCI values were ∼5 for PM, AM, and GM, and ∼2 for SUC ± ROT (Figure 4O). These model-predicted RCI values are consistent with our recent experimental study^1^ and signify how OxPhos has differential efficiency for ATP production for different metabolic substrates in different tissues.

### Model Predictions of Mitochondrial Bioenergetics Under Pathological Conditions of Increased Proton Leak (UCP Activity) and Mitochondrial Uncoupling

**Figure 5.**
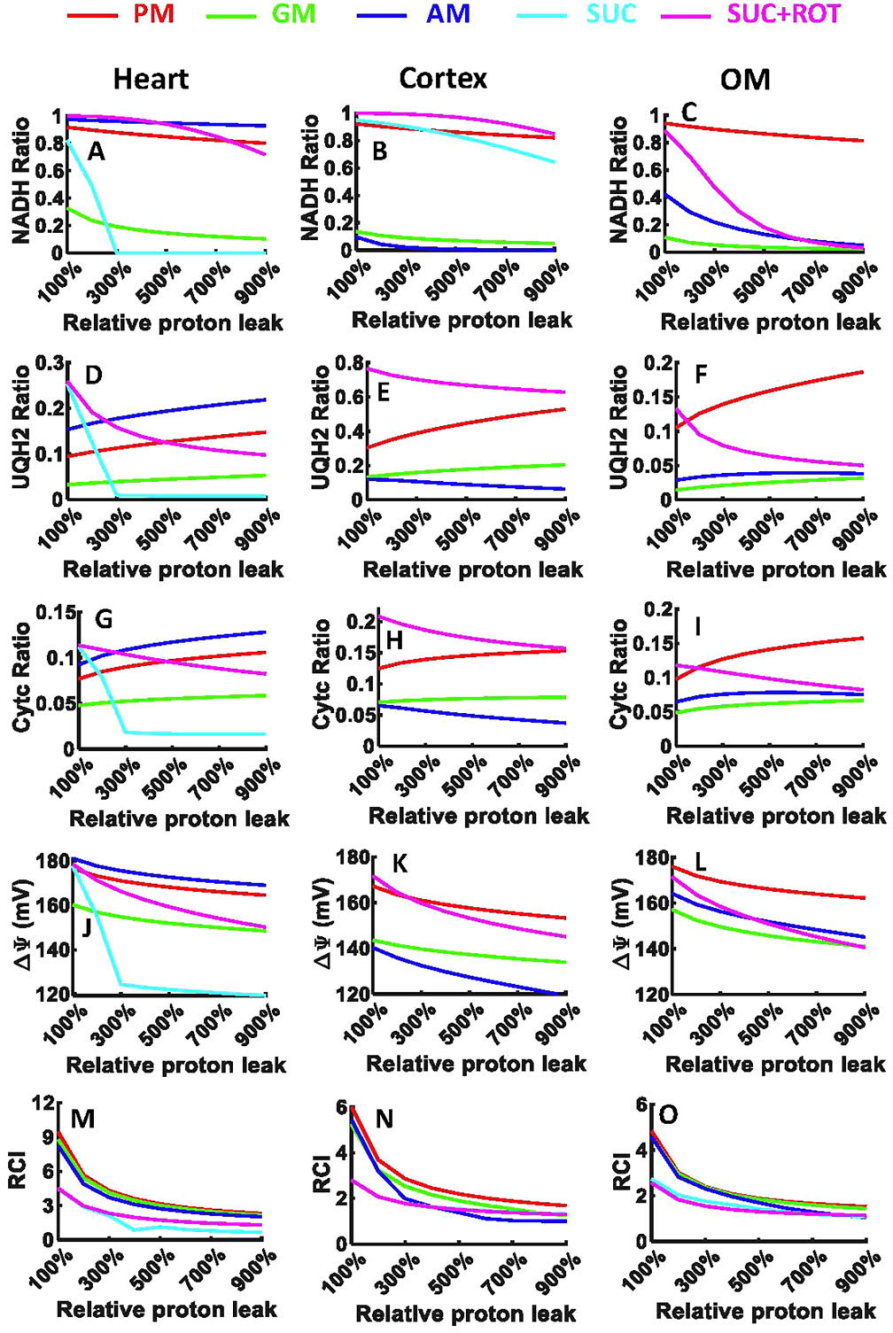
Model predictions of redox ratios, ${{\bf \Delta }}{{{\bf \Psi }}}_{\boldsymbol{m}}$, and RCI in response to increasing H^+^ leak in the heart and kidney cortex and OM mitochondria. Model predictions of mitochondrial (A–C) NADH ratio [C_NADH/_(C_NADH_ + C_NAD_)], (D–F) UQH_2_ ratio [C_UQH2/_(C_UQH2_ + C_UQ_)], (G–I) CytCred ratio [C_CytCred/_(C_CytCred_ + C_CytCoxi_)], (J–L) ${\rm{\Delta }}{{\rm{\Psi }}}_m$, and (M–O) RCI in the presence of 5 different substrate combinations including PM (5:2.5 m m), AM (5:2.5 m m), GM (5:2.5 m m), SUC (10 m m), and SUC + ROT (10 m m + 0.5 µm) in response to increasing H^+^ leak from 100% (control) to 900% at fixed ADP of 200 µm in the heart and ADP of 100 µm in the kidney cortex, and OM mitochondria. Some model simulations for SUC and SUC + ROT overlap in the kidney cortex and OM mitochondria (pink and cyan lines).

The validated models were also used to simulate alterations of key bioenergetic state variables and emergent metabolic system properties in response to increased H^+^ leak (UCP2) activity, which can uncouple OxPhos and lead to a pathological condition in isolated heart and kidney cortex and OM mitochondria (Figure 5). The key model predictions included NADH ratio, UQH_2_ ratio, CytCred ratio, ${\rm{\Delta }}{{\rm{\Psi }}}_m$, and RCI (state 3 *J*_O2_/state 2 *J*_O2_). The pathological condition was simulated by increasing the maximal H^+^ leak activity parameter (*T*_max_) from 100% to 900%, where 100% *T*_max_ represents the normal physiological condition as represented in Figures 2–4, and 200% to 900% *T*_max_ represent the progressive pathological condition of increased H^+^ leak and uncoupling of OxPhos. Mitochondrial respiratory and bioenergetic responses to a single dose of ADP (200 μm for the heart mitochondria and 100 µm for the kidney cortex and OM mitochondria) were simulated in the presence of different substrates as in Figure 3 (experimental protocol of Figure S1B). For all 3 tissues, model simulations predicted that in the presence of NADH-linked substrates, the state 2 NADH ratio, UQH_2_ ratio, and CytCred ratio following substrate addition did not change appreciably despite relatively large changes in the presence of FADH_2_-linked substrates.

In state 2, the NAD pool was majorly oxidized in the presence of SUC in the heart and kidney OM mitochondria despite minor oxidation in the kidney cortex mitochondria (Figure 5A–C). In the presence of SUC + ROT, the state 2 NAD pool was only slightly oxidized in the heart and kidney cortex mitochondria, despite major oxidation in the kidney OM mitochondria (Figure 5A–C). In state 2, the UQ and CytC pools were majorly oxidized in the presence of SUC despite their minor oxidation in the presence of SUC + ROT in the heart mitochondria (Figure 5D and G). In the kidney cortex and OM mitochondria, the state 2 UQ and CytC pools were slightly oxidized in the presence of SUC with or without ROT (Figure 5E–F, H–I).

The state 2 reduction of ${\rm{\Delta }}{{\rm{\Psi }}}_{\rm{m}}$ with increased H^+^ leak activity was minor in the presence of NADH-linked substrates and SUC + ROT in all 3 tissues, consistent with minor changes in the redox ratios (Figure 5J–L). However, the state 2 ${\rm{\Delta }}{{\rm{\Psi }}}_{\rm{m}}$ was appreciably reduced (120 mV) with increased H^+^ leak activity in the presence of SUC in the heart mitochondria despite minor reduction in the kidney cortex and OM mitochondria (Figure 5J–L).

In the control condition (100% *T*_max_ for H^+^ leak), the RCI values were highest in the heart mitochondria followed by the kidney cortex and OM mitochondria. In addition, the RCI values were appreciably higher for NADH-linked substrates compared to FADH_2_-linked substrates. These results are consistent with our recent experimental study.^1^ In all 3 tissues, the RCI values progressively decreased with increased H^+^ leak activity in the presence of both NADH-linked and FADH_2_-linked substrates, albeit appreciable decrease in the heart and kidney cortex mitochondria compared to the kidney OM mitochondria. The reduction in the RCI values in the presence of SUC ± ROT was minor in all 3 tissues (Figure 5M–O).

### Model Predictions of Differential Tissue-Specific Mitochondrial Bioenergetics Responses to SUC vs. SUC + ROT

**Figure 6.**
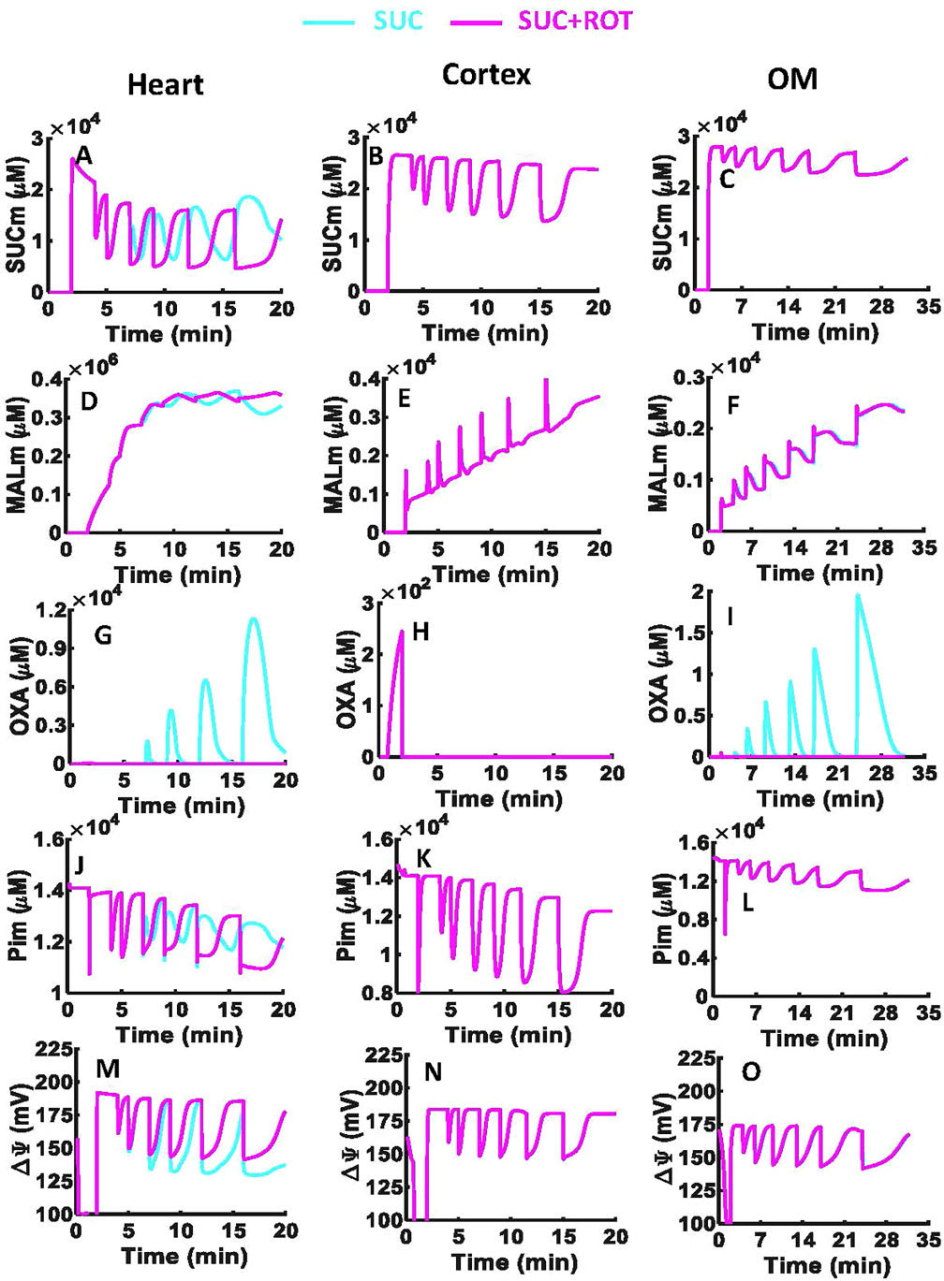
Model predictions of key mitochondrial metabolites and ${{\bf \Delta }}{{{\bf \Psi }}}_{\boldsymbol{m}}$ with considerably different dynamics in the presence of SUC vs. SUC + ROT in the heart and kidney cortex and OM. Model simulations of the time courses of metabolite profiles including SUC (A–C), MAL (D–F), OXA (G–I), and Pi (J–L) in the mitochondrial matrix and ${\rm{\Delta }}{{\rm{\Psi }}}_m$ across mitochondrial inner membrane (M–O) in the presence of SUC ± ROT (10 m m ± 0.5 µm) in response to sequential ADP additions of 25, 50, 75, 100, 150, and 250 µm in the heart and kidney cortex and OM mitochondria. The model predictions are done in the presence of 0.05 mg/mL mitochondrial for the heart and 0.2 mg/mL mitochondria for the kidney cortex and OM. Some model simulations for SUC and SUC + ROT overlap in the kidney cortex and OM mitochondria (pink and cyan lines).

**Figure 7.**
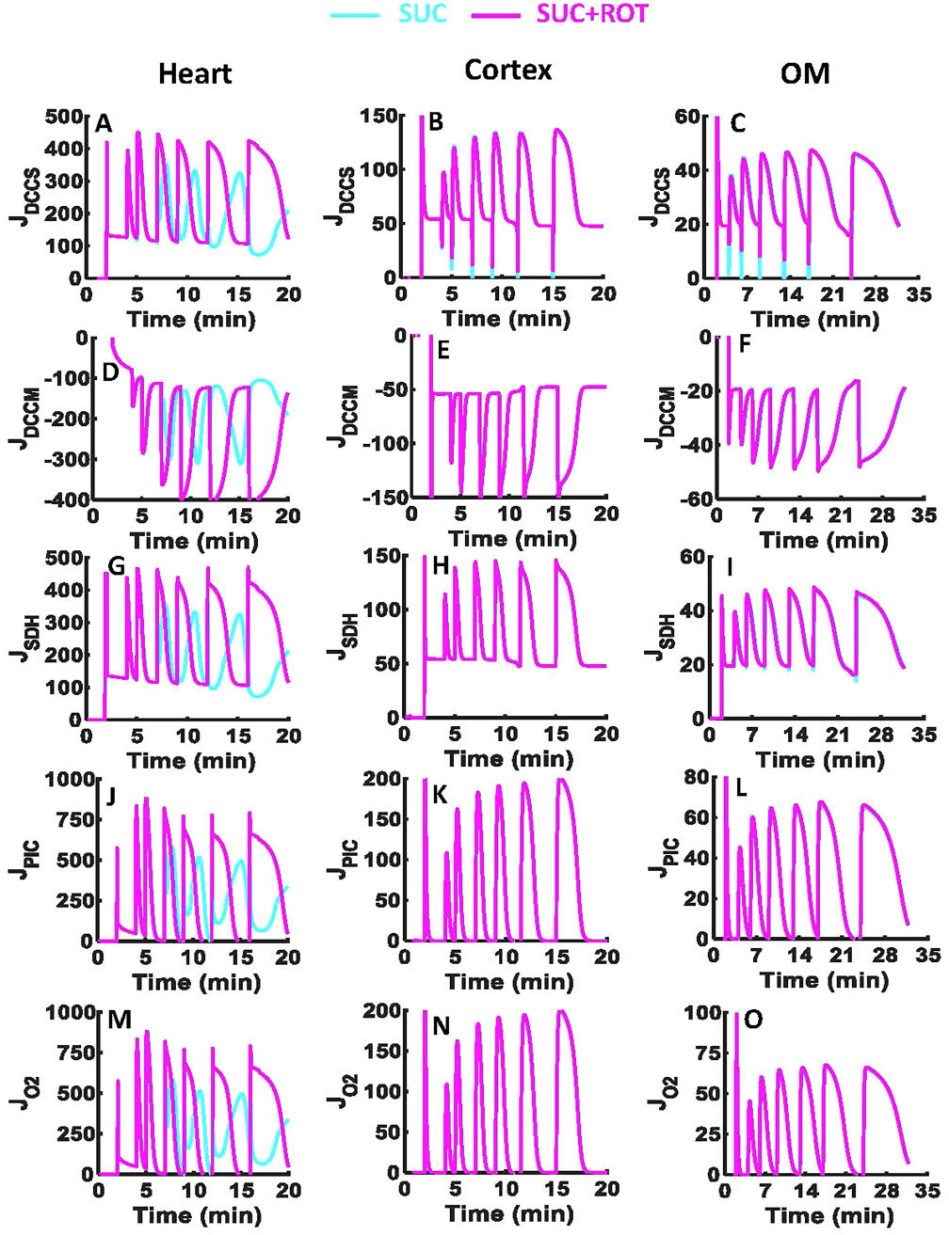
Model predictions of key mitochondrial metabolic fluxes with considerably different dynamics in the presence of SUC vs. SUC + ROT in the heart and kidney cortex and OM. Model simulations of the time courses of metabolic fluxes including *J*_DCCS_ (A–C), *J*_DCCM_ (D–F), *J*_SDH_ (G–I), *J*_PiC_ (J–L), and *J*_O2_ (M–O) in the presence of SUC ± ROT (10 m m ± 0.5 µm) in response to sequential ADP additions of 25, 50, 75, 100, 150, and 250 µm in the heart and kidney cortex and OM mitochondria. All fluxes are in the unit of nmol/min/mg of mitochondria. Some model simulations for SUC and SUC + ROT overlap in the kidney cortex and OM mitochondria (pink and cyan lines).

To explore the mechanisms underlying the differential oxidation of SUC vs. SUC + ROT in the 3 tissues, model simulations were conducted and analyzed as shown in Figures 6 and 7. These simulations reveal major differences in the FADH_2_-linked ETC reactions, TCA cycle enzymes, and metabolite transporters in the heart mitochondria, but not in the kidney cortical or OM mitochondria (Figures 6, 7, S6B, D, F, and S7B, D, F).

In the heart mitochondria, the comparison of SUC vs. SUC + ROT profiles showed appreciable differences in ${\rm{\Delta }}{{\rm{\Psi }}}_{\rm{m}}$ dynamics and in the dynamics of metabolites involved in the FADH_2_-linked pathway including SUC_m_, MAL_m_, OXA_m_, and Pi_m_ (Figure 6). However, in the kidney cortex and OM mitochondria, all metabolites and ${\rm{\Delta }}{{\rm{\Psi }}}_{\rm{m}}$ dynamics were similar except for OXA_m_. The heart and kidney cortex and OM mitochondria did not produce OXA in the presence of SUC + ROT due to fully reduced NAD pool (ie, NADH ratio is ∼100%). In the heart and kidney cortex and OM mitochondria before substrate addition, OXA is produced by GOT reaction of indigenous AKG and ASP in the mitochondrial matrix (Figures S6 and S7). In the heart and kidney OM mitochondria, the concentration of OXA is relatively higher after SUC and ADP additions compared to that before SUC addition due to highly oxidized NAD pool, which suppresses the initial OXA concentration (Figure 6G, I). In contrast, in the kidney cortex mitochondria, after additions of SUC and ADP, NADH is only minimally oxidized to NAD^+^ at CI, compared to that in the heart and kidney OM mitochondria. Therefore, despite of the high MAL availability, OXA production is limited by reduced NAD pool, and hence OXA concentration after SUC addition is smaller than OXA concentration before SUC addition. In the presence of SUC, the OXA concentration was ∼10^4^ times higher in the heart mitochondria than in the kidney OM mitochondria and was negligible in the kidney cortex mitochondria (Figure 6G–I). The MAL produced after each ADP addition is accumulated in the mitochondrial matrix for all tissues, but this MAL accumulation was greater in the heart mitochondria than in the kidney cortex mitochondria, which in turn was higher than that in the kidney OM mitochondria (Figure 6D–F). In the heart mitochondria, ${\rm{\Delta }}{{\rm{\Psi }}}_{\rm{m}}$ was lower in the presence of SUC compared to SUC + ROT. However, ${\rm{\Delta }}{{\rm{\Psi }}}_{\rm{m}}$ in the presence of SUC with or without ROT were similar in the kidney cortex and OM mitochondria (Figure 6N, O).

The model analysis revealed appreciable differences in metabolic fluxes related to the FADH_2_ pathway, including ETC SDH/CII and CIV complexes and DCCM, DCCS, and phosphate carrier (PiC) transporters, in the heart mitochondria between SUC vs. SUC + ROT, but not in the kidney cortex and OM mitochondria (Figure 7). In the heart mitochondria oxidizing SUC, the influx rate of SUC_e_ and efflux rate of MAL_m_ to and from the mitochondrial matrix were reduced after the second dose of ADP (75 µm), indicting inhibition of SUC_e_ influx and MAL_m_ efflux (Figure 7A, D). In addition, the rate of SUC oxidation by SDH was decreased, which was due to inhibition of SDH by OXA_m_ (Figure 7G). Our model also predicts reduction in the rate of Pi influx to the mitochondrial matrix by PiC in response to high concentration of Pi_m_ and decreased O_2_ reduction rate by CIV (Figure 7J, M). These responses were not evident in simulations of the heart mitochondria oxidizing SUC + ROT. In addition, the model-predicted responses of the kidney cortex and OM mitochondria were identical for SUC and SUC + ROT (Figure 7, the middle and right columns).

## Discussion

There is ample evidence that the kinetics and efficiency of mitochondrial O_2_ consumption (respiration) for ATP production depend on the choice of metabolic substrates being utilized.^1,20^ Different substrates differentially generate the reducing equivalents NADH and FADH_2_ via the TCA cycle, which feed electrons to the ETC that drive OxPhos and ATP synthesis. However, the precise contributions of these substrates have not been systematically and quantitatively characterized in mitochondria of the heart or the kidney, the 2 major energy consuming organs in our body.^1–5^ The present modeling study aimed to fill this knowledge gap, given the critical role that mitochondrial respiratory and bioenergetic dysfunctions play in these organs in the context of cardiovascular and renal diseases such as hypertension.^6–14^ The pathogenesis of a cardiac disease contributes to the pathogenesis of a renal disease and vice versa due to the interconnections among processes driving cardiovascular and renal diseases.^47–51^

In our recent experimental study,^1^ we reported appreciable differences in substrate-dependent respiration and bioenergetics between isolated mitochondria from normal SD rat heart and kidney cortex and OM. Specifically, heart mitochondria showed predominantly higher respiratory rates (OCR) and membrane potential (ΔΨ_m_) for both NADH- and FADH_2_-linked substrates compared to the kidney cortex and OM mitochondria. Additionally, OxPhos efficiency in the heart mitochondria was higher for NADH-linked substrates and lower for FADH_2_-linked substrates compared to the kidney cortex and OM mitochondria. We also observed major differences in mitochondrial respiration and ROS production in the presence of SUC with and without ROT (complex I inhibitor) in the heart, whereas only minor differences were observed in the kidney OM and no differences were observed in the kidney cortex.^1^ When SUC is used as substrate, heart mitochondria produce excess ROS under states 2 and 4 via reverse electron transfer (RET) at complex I (CI) and excess oxaloacetate (OXA) in state 3 via TCA cycle, which subsequently inhibits SDH reaction and alters respiration, OxPhos, and ROS production.^1,45,46,52^ Inhibition of CI by ROT stalls the RET-mediated ROS production and improves mitochondrial respiration and OxPhos.^1^ However, why that is not the case for kidney cortex and OM mitochondria is not well known. To investigate these diverse kinetic data and begin to elucidate differences in underlying mechanisms, we developed computational models of mitochondrial respiration and bioenergetics. These models and resulting simulations provided a deeper quantitative understanding of the mechanisms responsible for the differential responses of substrate-dependent mitochondrial respiration and bioenergetics in the heart and kidney cortex and OM.

### Previous Computational Mitochondrial Models and Their Limitations

Other groups have also developed models of mitochondrial respiration and bioenergetics at different levels of complexity, depending on the questions being addressed. As such, models have improved over the years to incorporate our expanding knowledge of mitochondrial function and dysfunction in health and diseases. Korzeniewski and colleagues^53^ developed one of the first mitochondrial OxPhos models which included simple mass action kinetics for complexes I, III, IV, and V, proton leak, inorganic PiC, and ANT for ATP/ADP exchange in skeletal muscle. Subsequently, they conducted a series of theoretical studies to understand various potential regulation mechanisms of OxPhos and ATP production in skeletal muscle during different types of exercises (eg, see^54–56^). Saito et al.^57^ expanded upon the model developed by Korzeniewski and colleagues^53^ by integrating the kinetics of TCA cycle reactions and cation transporters. They then used this model to investigate how Ca^2+^ ions regulate respiration and OxPhos in isolated cardiac mitochondria, specifically in the presence of glutamate and malate substrates. They also used the model to understand the mechanisms that maintain cardiac levels of energy metabolites constant during changes in workload in vivo. Cortassa et al.^26,27^ developed a detailed integrated model of cardiac mitochondrial energy metabolism and Ca^2+^ dynamics that included the mechanistic enzymatic kinetics of TCA cycle, ETC and OxPhos, and Ca^2+^ handling, with a goal of understanding different regulation mechanisms of OxPhos and ATP synthesis in the heart during excitation–contraction coupling.

Beard et al.^36^ developed a thermodynamically constrained mitochondrial OxPhos model including complexes I, III, IV, and V, proton leak, potassium transport, PiC, and ANT using simple mass action kinetics but accounting for Gibb's free energy of reactions. Heiske et al.^58^ then developed a hybrid OxPhos model based on Beard’s^36^ and Korzeniewski’s^59^ models using Michaelis–Menten kinetics instead of mass action kinetics for reactions and transporters. Dash and coworkers^32–34^ further extended Beard’s OxPhos model^36^ and developed integrated models of cardiac mitochondria that accounted for mitochondrial Na^+^ and Ca^2+^ transports and Ca^2+^ sequestration. Bazil et al.^30^ recently modified Beard’s OxPhos model^36^ to develop a hybrid model of isolated rat heart mitochondria that included the kinetics of ETC and OxPhos, PiC, ANT, H^+^ leak, and K^+^ transport to study regulation of mitochondrial OxPhos and ROS production under physiological conditions with imposed workloads. However, these models did not include TCA cycle reactions. In separate studies, Wu et al.^35^ integrated Beard’s OxPhos model^36^ with the kinetics of TCA cycle reactions (including SDH/complex II) using data from rat heart and skeletal muscle mitochondria, and studied the effects of NADH-linked substrates including pyruvate, malate, and glutamate on mitochondrial respiration and bioenergetics. However, their model^35^ did not consider the effects of other NADH-linked substrates such as alpha-ketoglutarate and FADH_2_-linked substrate such as succinate. Wu et al.^60,61^ later extended their model^35^ to study regulations of phosphate and energetic metabolites during ischemia and exercise in the heart and skeletal muscle in vivo. Bazil et al.^31^ later extended Wu et al. model^35^ to include cation handling from Dash and Beard^34^ and study the regulation of mitochondrial bioenergetics and volume dynamics.

Despite numerous computational studies on heart mitochondria, existing models have limited applicability in describing mitochondrial respiration and bioenergetics of other organs such as kidneys. Edwards et al.^37^ recently modified Wu et al. model^35^ to study oxygen consumption and ATP production of kidney proximal tubular (PT) cells. However, their model cannot account for the differential mitochondrial respiration and bioenergetics between the kidney cortex and OM and their distinct responses to different metabolic substrates^1^. It is known that different segments of nephron (eg, PT and mTAL) and different regions of kidney (eg, cortex and OM) have varying mitochondrial contents, energy requirements, metabolic rates, and oxygen consumptions. Our present computational modeling focuses on the PT and mTAL segments and cortex and OM regions of the kidney, which are metabolically active segments and regions involved in reabsorption of filtered sodium and represent an important step forward in the field.

### Important Aspects of the Current Computational Mitochondrial Models: Relevance to Tissue/Organ Bioenergetic Functions

The present models of mitochondrial respiration and bioenergetics in the heart and kidney cortex and OM were developed based on the lung tissue mitochondrial respiration and bioenergetics model that we previously developed.^28^ To account for potential differences in mitochondrial bioenergetics between these organs, we incorporated tissue-specific and substrate-dependent regulations of enzymatic and transporter reactions into the model. The intrinsic model parameters such as Michaelis–Menten constants (*K*_m_’s) characterizing the binding of metabolites to enzymes and transporters were kept constant and set to the same values as in our previous model^28^ for all enzymatic reactions and metabolite transporters unless otherwise mentioned. The extrinsic model parameters such as maximal reaction and transporter velocities (*V*_max_’s and *T*_max_’s) were estimated to account for possible differences in tissue-specific enzyme and transporter activities.

The present models of mitochondrial respiration and bioenergetics were parameterized and validated (Figures 2 and 3) using a diverse set of experimental data recently published by our laboratory.^1^ The models were parameterized separately for each tissue by fitting the model solutions for *J*_O2_ or *J*_CIV_ fluxes to the OCR data obtained using 5 different metabolic substrate combinations with sequential and incremental ADP additions to the isolated heart and kidney cortex and OM mitochondria (Figure 2). The substrate combinations included those which activate both NADH and FADH_2_-linked metabolic pathways. For a given tissue, the ability of the corresponding model simulations to fit well all the substrate-dependent experimental data suggests that the model accounts for the dominant processes that affect mitochondrial respiration and bioenergetics for the different substrate combinations studied. The optimal estimates of the tissue-specific *V*_max_ and *T*_max_ parameters are given in Table 2 and Figure S2. The quality control measures performed on the optimal parameter estimates (ie, parameter sensitivity analyses and correlation coefficient matrices; Figures S3–S5) suggest robustness and high confidence in the estimated values of most of the model parameters.

As postulated, the estimated *V*_max_ and *T*_max_ values were largely different from each other in a specific tissue, indicating differential expressions, activities, and regulations of different enzymes and transporters in that tissue to optimally perform the individual metabolic functions. In addition, the estimated *V*_max_ and *T*_max_ values were several folds higher in heart mitochondria than in kidney cortex mitochondria, and several folds higher in kidney cortex mitochondria than in kidney OM mitochondria, consistent with the measured OCR data.^1^ These findings are also consistent with the general notion that differential expressions of enzymes and transporters and their catalytic activities and regulations in different tissues (*viz*., heart and kidney cortex and OM) are required for those tissues to optimally perform their distinct metabolic functions.

The present models were further validated and strengthened by their abilities to predict isolated heart and kidney cortex and OM mitochondrial OCR and ΔΨ_m_ responses to the same 5 metabolic substrate combinations activating both NADH and FADH_2_-linked pathways followed by stimulation of single dose of ADP addition,^1^ which were not used for the estimation of tissue-specific *V*_max_ and *T*_max_ parameters (Figure 3). Fairly good correspondence between model simulations and experimental data signifies robust model validation and corroboration.

The present models not only adequately captured the tissue-specific and substrate-dependent experimental data on mitochondrial respiration and ΔΨ_m_ (Figures 2 and 3), but also allowed for prediction of emergent metabolic system properties such as mitochondrial redox states, enzyme and transporter fluxes, metabolite concentrations, respiratory control index (RCI = state 3 OCR/state 2 OCR), and ΔΨ_m_ under diverse physiological and pathological perturbations (Figures 4, 5, S6, and S7). This enabled prediction of changes in various metabolic fluxes and metabolite concentrations that are difficult to measure experimentally, which helped in elucidating the underlying mechanisms correlating observed changes of mitochondrial respiration and bioenergetics in the heart and kidney cortex and OM. Overall, the validated models and their predictions of emergent metabolic system properties further our understanding of mitochondrial metabolism, OxPhos, and bioenergetics in the heart and kidney cortex and OM under physiological and pathological conditions.

Interestingly, the model simulations predicted that the heart and kidney cortex and OM mitochondrial redox states and bioenergetics are not very prone to mild increases in proton leak in the presence of NADH-linked substrates (Figure 5). The same was also predicted to be the case in the kidney cortex mitochondria in the presence of FADH_2_-linked substrates (SUC ± ROT). However, the heart and kidney OM mitochondria were predicted to be very sensitive to mild increases in proton leak in the presence of the FADH_2_-linked substrate SUC, resulting in appreciable changes in their emergent metabolic system properties. These results indicate that mitochondrial SUC accumulation combined with increased uncoupling of OxPhos is detrimental to the bioenergetic function of the heart and kidney OM mitochondria due to considerable changes in their emergent metabolic system properties, including redox states.

Additionally, the model simulations revealed that the low respiratory responses of GM in all 3 tissues could be attributed to high concentrations of ASP in the mitochondrial matrix. The simulations demonstrated that these higher concentrations caused the electrogenic GLU/ASP exchanger (GAE) to utilize ASP_m_ in exchange for GLU_e_, and the GLUH cotransporter to work in the opposite direction resulting in extrusion of H^+^_m_ and GLU_m_ from the mitochondrial matrix (Figures S6 and S7). Consequently, GLU_e_ influx was limited only to the GAE antiporter rather than the GLUH cotransporter. Additionally, the model predictions showed that the activity of AKGDH was several orders of magnitude lower in kidney cortex and OM mitochondria than in heart mitochondria (Table 2 and Figure S2). These findings provide valuable insights into the differences in mitochondrial respiration and bioenergetics among different tissues, which could have significant implications in our understanding of various pathological states associated with metabolic dysfunctions of the heart and kidney.

**Figure 8.**
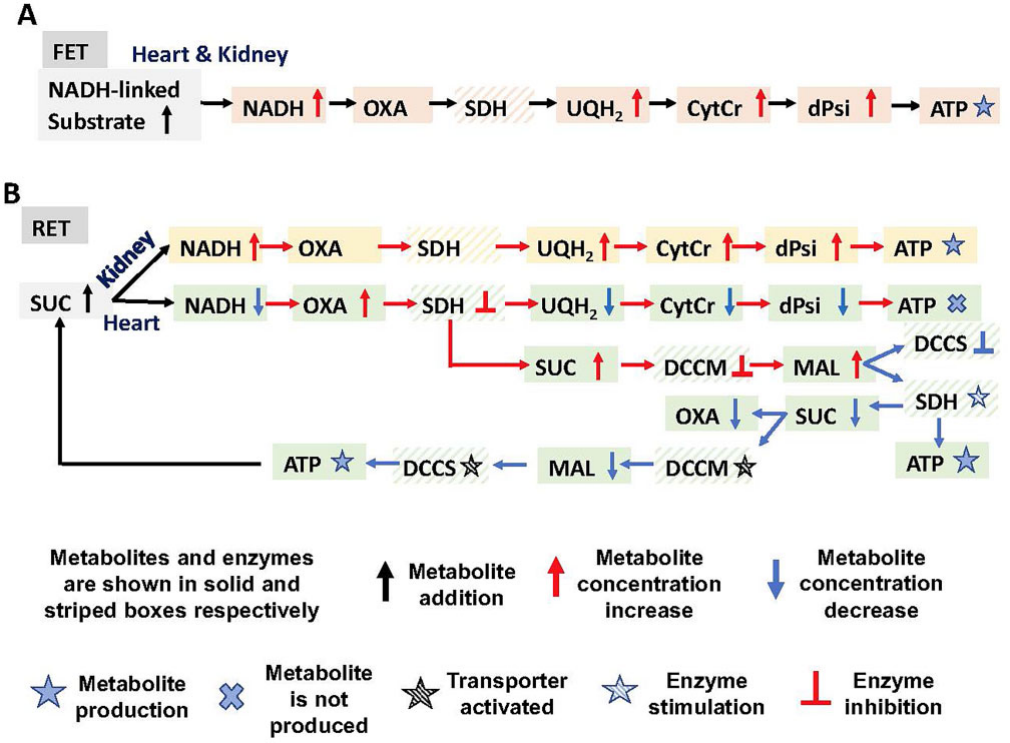
Schematics of the NADH vs. FADH_2_ pathway differences and regulations between the heart and kidney cortex and OM mitochondria. The diagram includes metabolites, transporters, and TCA cycle enzymes involved in (A) forward electron transfer (FET) through NADH pathway for the heart and kidney cortex and OM mitochondria in brown and (B) reverse electron transfer (RET) through FADH_2_ pathway for the kidney cortex and OM mitochondria in yellow and heart mitochondria in green. The black arrows show the control addition or production of metabolites, the red arrows show considerably high increase in concentration of metabolites, and blue arrows show decrease in concentration of metabolites. The solid blue stars show that ATP is produced, and the solid blue multiplication sign show that ATP is not produced. The blue striped stars show that an enzyme or transporter is stimulated, and the black striped stars show that an enzyme or transporter is activated.

The model simulations not only revealed major differences in mitochondrial respiratory and bioenergetics responses among different tissues, but also provided insights into the likely mechanisms underlying differences in metabolic fluxes and metabolite concentrations associated with SUC vs. SUC + ROT oxidation in the heart mitochondria vs. kidney cortex and OM mitochondria (Figures 6 and 7). Specifically, the high concentration of SUC in the heart mitochondria was predicted to inhibit the DCCM resulting in MAL_m_ accumulation, which then lead to more OXA_m_ production and SDH inhibition by OXA_m_ (Figure 8B). Excess MAL_m_ accumulation also inhibited the DCCS reducing the influx of SUC_e_ to the mitochondrial matrix and reversing OXA_m_ and MAL_m_ accumulation (Figure 8B). This mechanism was found highly prominent in the heart mitochondria and not in the kidney cortex or OM mitochondria, due to the lower affinity of MAL_m_ binding to DCCS in the kidney cortex and OM mitochondria.

### Model Predictions of the Differences in NADH-linked Pathway Fluxes and Metabolite Profiles Among the Heart and Kidney Cortex and OM Mitochondria

In the presence of NADH-linked substrates (ie, during FET), the NAD pool is majorly reduced followed by reduced UQ pool and CytC pool leading to OxPhos and ATP production (Figure 8A). For NADH-linked substrates, the mitochondrial OCR, ATP synthesis rate, and other intermediate metabolic fluxes were highest in the presence of PM and lowest in the presence of GM in all 3 tissues studied. This is consistent with the estimated higher *V*_max_ values for PDH and lower *V*_max_ values for GOT shown in Figure S2. Interestingly, metabolic fluxes in the presence of AM were as high as those in the presence of PM in the heart mitochondria but as low as those in the presence of GM in the kidney cortex and OM mitochondria as demonstrated in Figures 2 and S6–S7. This can be explained by the higher value of *V*_max_ for AKGDH in the heart mitochondria compared to the kidney cortex and OM mitochondria (Figure S2).

Moreover, mitochondria utilizing GM yielded the lowest OCR in all 3 tissues (Figure 2), and the model predicts that this can be attributed to the limitations of GLU influx via the GAE antiporter and GLUH cotransporter, as well as limitations of oxidations by GOT and AKGDH (Figures S6 and S7). GLU transport is limited to the electrogenic GAE antiporter rather than the GLUH cotransporter due to high ASP concentrations which are produced when mitochondria are energized with GLU. This phenomenon has been reported in previous studies,^62^ which demonstrated that GAE antiporter plays a vital role in controlling mitochondrial respiration. Other studies have suggested that reduction of the GLUH cotransporter activity for GLU transport could be explained by a decrease in matrix pH.^63^ Our model predictions support this idea, as evidence by the reduced ${\rm{\Delta }}{{\rm{\Psi }}}_m$ during state 3 respiration in the presence of GM compared to other substrates as shown in Figures 3 and 4. In addition, the model predicts a considerably oxidized NAD pool (ie, low NADH) in mitochondria utilizing GM for all 3 tissues.

Considering all things together regarding GM substrate, our model analyses and supporting data indicate that protons are transferred to the mitochondrial matrix via GAE. The addition of ADP and mitochondrial ATP synthesis leads to an imbalance of the proton gradient across the IMM, resulting in the GLUH cotransporter working in the opposite direction and extruding protons, which reduces the proton gradient along with GLU. This negative transport of protons and GLU is negligible in all tissues, as shown in Figure S6.

### Model Predictions of the Differences in FADH_2_-linked Pathway Fluxes and Metabolite Profiles Among the Heart and Kidney Cortex and OM Mitochondria

Alterations in SDH activity associated with pathological conditions can significantly affect mitochondrial OxPhos and ROS production by affecting SUC oxidation, as reported by previous studies.^52,64,65^ In the heart, increased oxidation of SUC (via SDH) and the associated elevation in mitochondrial membrane potential (${\rm{\Delta }}{{\rm{\Psi }}}_{\rm{m}})$ have also been shown to drive mitochondrial ROS production.^52^ In addition, we have recently shown that there are significant differences in mitochondrial respiration and ROS production between the heart and kidney cortex and OM in the presence of SUC ± ROT.^1^ In the present study, computational modeling was used to develop hypotheses that may explain the differences observed in the oxidation of SUC vs. SUC + ROT in these 3 tissues (Figure 8B).

In contrast to the heart, in the kidney cortex and OM, the mitochondrial OCR dynamics during the sequential and incremental ADP additions were similar when utilizing SUC or SUC + ROT (Figure 2). These differences appear to be explained by excess OXA production and accumulation in the heart mitochondria compared to the kidney cortex and OM mitochondria in the presence of SUC which inhibits SDH and electron transfer to the ETC resulting in changes in the OCR and ATP synthesis rate (Figure 8B), consistent with previous differential results in different tissues .^45,46,66^ As shown in Figures 6 and 7, the model also predicts oscillating OXA dynamics in the heart and kidney OM mitochondria with sequential and incremental ADP additions in the presence of SUC, during which ascending and descending of OXA concentrations are synchronized with similar oscillations in the SDH flux and OCR. It was observed that in the presence of SUC, the DCCS flux was positive (SUC entering mitochondria and Pi exiting) while DCCM flux was negative (MAL exiting mitochondria and Pi entering). The model suggests that with SUC influx to the mitochondrial matrix and SDH inhibition by OXA accumulation, there is an accumulation of SUC, which inhibits DCCM slowing the outflow of MAL. This results in accumulation of MAL in the mitochondrial matrix which inhibits DCCS reducing SUC influx and stimulates SDH increasing SUC oxidation. This results in reduction in SUC concentration in the mitochondrial matrix which reverses DCCM inhibition and increases MAL outflux. This leads to a reduction of OXA in the mitochondrial matrix which reverses the SDH inhibition. Thus, reversing SDH inhibition and stimulating SDH facilitates the transfer of electrons to the ETC and increases the OCR. This mechanism results in synchronized oscillating dynamics in OXA concentration, ATP synthesis, SDH flux, and OCR. Our studies found that this phenomenon is considerably less prominent in the kidney cortex and OM mitochondria.

Interestingly, in the heart mitochondria, the concentrations of accumulated FUM and MAL with sequential and incremental ADP additions in the presence of the FADH_2_-linked substrates SUC ± ROT were predicted to be several folds higher than those in the presence of NADH-linked substrates. However, in the kidney cortex and OM mitochondria, the concentrations of accumulated FUM and MAL with the same sequential and incremental ADP additions in the presence of the FADH_2_-linked substrates SUC ± ROT were predicted to be considerably lower than those in the presence of NADH-linked substrates (Figure S7). Moreover, the dynamics of DCCM flux in the heart mitochondria were predicted to be considerably different from that in the kidney cortex and OM mitochondria with sequential and incremental ADP additions (Figures 7 and S6). These findings are consistent with our proposed hypothesis that the inhibitory binding constants of MAL and SUC for the DCCS and DCCM transporters are different between the heart and kidney cortex and OM (Figure 8B). SUC has the highest binding affinity for DCCM in the heart mitochondria, resulting in more potent inhibition of DCCM by SUC and accumulation of MAL. In contrast, the binding affinity of MAL for DCCS in the heart mitochondria is the lowest, resulting in less potent inhibition of DCCS by MAL and more influx of SUC. This in turn leads to more MAL_m_ being required to inhibit DCCS to slow the SUC influx. In the kidney cortex and OM mitochondria, SUC binding affinity for DCCM is lower, inhibiting DCCM to a lesser extent and allowing for more efflux of MAL_m_. Also, the higher binding affinity of MAL for DCCS inhibits DCCS to a greater extent and stalls the influx of SUC.

### Model Predictions of the Differences in Redox Ratios and Bioenergetics Among the Heart and Kidney Cortex and OM Mitochondria in Physiological Conditions

The model predicts that when NADH-linked substrates are added to the mitochondria leading to H^+^ leak state respiration (state 2), the NAD pool is reduced due to NADH production via the dehydrogenase enzymes in the TCA cycle. However, in mitochondria stimulated by ADP (state 3), the NAD pool is oxidized by complex I due to OxPhos. It is worth noting that, among the NADH-linked substrates, GM shows the lowest reduced NAD pool in the heart mitochondria, and GM and AM produce the lowest reduced NAD pool in the kidney cortex and OM mitochondria. Additionally, the UQ pool and CytC pool are reduced in the H^+^ leak state and further reduced by complexes I and III during OxPhos (Figures 4 and S7).

Similarly, the model predicts that when the FADH_2_-linked substrate SUC is added to the mitochondria, the NAD pool is fully reduced by complex I via RET in the H^+^ leak state, while the UQ pool and CytC pool are only partially reduced by complexes II and III, respectively. During OxPhos state (state 3), the NAD pool, UQ pool, and CytC pool are all oxidized. Inhibition of complex I by ROT blocks NADH oxidation and therefore the NADH ratio remains at its initial value. In the OxPhos state, the UQ pool and CytC pool are oxidized in manner similar to SUC (Figures 4 and S7).

Our model analyses suggest that when SUC is present in response to ADP addition (state 3), the NAD pool is 100% oxidized via FET by complex I in the heart mitochondria, only ∼20% in the kidney cortex mitochondria, and >90% in the kidney OM mitochondria. Moreover, we found that RET exists in the heart mitochondria to a greater extent than the kidney OM mitochondria while the kidney cortex mitochondria exhibit intermediate levels of RET (Figure S7). Additionally, during OxPhos (state 3) in the presence of SUC, the UQ and CytC pools are the same in the kidney cortex and OM mitochondria, indicating that RET is not a dominant mechanism in these mitochondria compared to heart mitochondria (Figure S7).

Mitochondrial RCI (state 3 OCR/state 2 OCR) was higher in the presence of NADH-linked substrates compared to FADH_2_-linked substrates (Figure 4M–O). This indicates greater efficiency of mitochondrial OxPhos for ATP production via the NADH pathway. Among NADH-linked substrates, PM had the highest ${\rm{\Delta }}{{\rm{\Psi }}}_m$ and GM had the lowest ${\rm{\Delta }}{{\rm{\Psi }}}_m$ in all respiratory states, similar to the OCR trend (Figure 4J–L). The ${\rm{\Delta }}{{\rm{\Psi }}}_m$ in the presence of SUC + ROT was determined to be ∼150 mV in the 3 tissues during OxPhos, and with SUC alone in the kidney cortex and OM. In contrast, the heart mitochondria in the presence of SUC, ${\rm{\Delta }}{{\rm{\Psi }}}_m$ was found to be as low as ∼130 mV during OxPhos with enhancement of the proton motive force thereby contributing to the RET.

### Model Predictions of the Differences in Mitochondrial Redox Ratios and Bioenergetics in the Heart and Kidney Cortex and OM in Pathological Conditions

Multiple studies have found that the development and progression of heart^67^ and kidney^68^ failure is associated with mitochondrial uncoupling of OxPhos, which diminishes the production of ATP, enhances oxidative stress, and facilitates organ failure. The pathological condition of mitochondrial OxPhos uncoupling was simulated by increasing the proton leak (UCP2 activity) in the model and predicted tissue-specific and substrate-dependent changes in the mitochondrial emergent metabolic system properties (Figure 5). Interestingly, it was found that the redox ratios including NADH ratio, UQH_2_ ratio, and CytCred ratio did not change appreciably in the presence of NADH-linked substrates despite their considerable changes in the presence of FADH_2_-linked substrates in the 3 tissues. This indicates that in the presence of NADH-linked substrates with increased influx of protons into the mitochondrial matrix, the ETC complexes I, III, and IV compensate by pumping protons across the IMM at a higher capacity resulting in minimal changes in ${\rm{\Delta }}{{\rm{\Psi }}}_m$ in these 3 tissues. However, the RCI values were considerably reduced after tripling (3×) the proton leak in the presence of all substrates in all 3 tissues, demonstrating that increasing proton leak considerably affects mitochondrial respiration at state 2 rather than state 3. The redox ratios obtained at state 2, as shown in Figure 5, did not change with increase in the proton leak. Therefore, we concluded that the reductions in the RCI values were due to increased respiration at state 2. These model predictions provide important mechanistic insights explaining such observations as found in diabetic rats, which have increased mitochondrial UCP2 expression in the renal PT cells with mitochondrial uncoupling and increased O_2_ consumption, which appears to lead to progressive kidney damage.^68^

Similarly, mechanistic insights from the model predict that with greater oxidation of SUC by the heart, the resulting reduction of the proton motive force would reduce ATP synthesis and contribute to bioenergetic dysfunctions.^45,46,52^ Specifically, the model predicted that oxidation of SUC in the heart mitochondria with 3× increased proton leak led to considerable decreases in the redox ratios, indicating that the redox pools are oxidized. The excess OXA production by MDH inhibits SDH, which blocks electron flow upstream in the ETC. Consequently, complexes III and IV cannot pump protons across the IMM resulting in reduction of ${\rm{\Delta }}{{\rm{\Psi }}}_m$. However, when complex I is blocked by ROT electrons can move upstream ETC to complexes III and IV, pumping protons across IMM and resulting in a balanced H^+^ concentrations across the IMM with minimal changes in ${\rm{\Delta }}{{\rm{\Psi }}}_m$. Additionally, since NAD pool is fully reduced, NAD^+^ is not available for MDH to produce OXA, which does not inhibit SDH. In the kidney cortex and OM, the redox ratios and ${\rm{\Delta }}{{\rm{\Psi }}}_m$ did not change in the presence of SUC with or without ROT, indicating that SDH is not inhibited by OXA. Therefore, electrons can flow upstream of the ETC and complexes III and IV can pump protons across the IMM keeping H^+^ concentrations balanced across the IMM. Hence, the role of mitochondrial proton leak in metabolism and OxPhos alteration is more prominent in the heart than kidney cortex and OM particularly in the presence of SUC, supported by previous findings.^69^

## Overall Summary, Model Limitations, and Future Directions

In this study, we developed computational models of how the mitochondria of the heart and kidney cortex and outer medullary cells produce energy. We used kinetic data from our previous studies to build these models. The models were calibrated by comparing their results to the measurements of oxygen consumption in isolated mitochondria. We tested the models by predicting how mitochondria would function when exposed to different combinations of energy sources. The validated models were then used to make predictions about how mitochondria behave in normal and stressed (eg, disease) conditions. We looked at various aspects of mitochondrial function, such as biochemical reactions, substrate transporters, and the levels of different molecules. By using these methods, we gained insights into why mitochondrial in the heart and kidney cortex and outer medulla behave differently when they oxidize different energy sources.

Consistent with the experimental data, the models developed in this study allowed us to simulate how the mitochondria in the heart and kidney cortex and outer medullary cells respond to different food substances and different conditions. It was found that the responses of the mitochondria were considerably different depending on the type of tissue and energy source they were using. Interestingly, the heart mitochondria energized with succinate without rotenone (a complex I inhibitor) failed to produce a robust state 3 response after we added a high concentration of ADP. However, the mitochondria of the kidney cortex and outer medulla showed similar responses to succinate with or without rotenone, suggesting that a different regulatory mechanism is at play in these tissues. Our model simulations also revealed that oxaloacetate, an intermediate of the citric acid cycle, which inhibits the oxidation of succinate, accumulated more quickly in the heart mitochondria comparing to the kidney cortex and outer medullary mitochondria. Overall, these models helped to elucidate how different combinations of energy sources affect the way mitochondria produce ATP, the energy currency of cells, in the heart and kidney cortex and outer medulla.

As with all models, the 3 models have limitations. One major limitations of the current models is the exclusion of the regulatory roles of ROS, such as O_2_^•−^ and H_2_O_2_, on mitochondrial bioenergetics. It was assumed in the models that cation concentrations were constant as was the case in all the experimental data^1^ utilized in the present study. The extra-mitochondrial buffer was maintained at a physiological pH of 7.15 and had negligible concentrations of Ca^2+^ due to the presence of 1 m m EGTA (a Ca^2+^ chelator). In addition, the concentrations of K^+^ and Na^+^ in the buffer were kept at physiological levels of 140 and 10 m m, respectively. The experiments with isolated mitochondria were performed in the absence of Mg^2+^. The pH of the mitochondrial matrix was fixed at a physiological level of 7.55 to maintain an appropriate pH gradient and proton motive force across the mitochondrial inner membrane. Similarly, ROS concentrations were assumed to be within the physiological levels, meaning they were negligibly small and had no impact on mitochondrial respiration and bioenergetics.

In future versions of the model, the dynamics of pH, K^+^, Na^+^, Ca^2+^, and Mg^2+^ ions will be included^26,27^,^31–34^,^57^ to understand the differential regulatory roles of cations (eg, Ca^2+^) on mitochondrial respiration and bioenergetics in various metabolically active tissues, as we have shown recently in another experimental study.^70^ The kinetics of ROS production via the ETC^71,72^ and ROS scavenging via the glutathione and thioredoxin systems^73–76^ will also be included to model mitochondrial ROS homeostasis^77–79^ and better understand alternations in mitochondrial respiration and bioenergetics during the progression of cardiac and renal diseases such as SS hypertension.

## Supplementary Material

zqad038_Supplemental_File

## Data Availability

The model is developed in MatLab. The MatLab codes and the associated data files used to generate all the results in this manuscript can be found at https://github.com/MCWComputationalBiologyLab/Sadri_Function_2023. The MatLab codes and the datasets analyzed during the current study can also be obtained from the corresponding authors on request.
