# Supplementary material for: Computational Modeling of Substrate-Dependent Mitochondrial Respiration and Bioenergetics in the Heart and Kidney Cortex and Outer Medulla
Source: Function (Oxf). 2023 Jul 25;4(5):zqad038. doi: 10.1093/function/zqad038 (PMC10413947; doi:10.1093/function/zqad038)
Supplement: zqad038_Supplemental_File [file zqad038_supplemental_file.docx]

**Supplemental Materials: “Computational Modeling of Substrate-Dependent Mitochondrial Respiration and Bioenergetics in the Heart and Kidney Cortex and Outer Medulla”**

Shima Sadri^1^, Xiao Zhang^1^, Said H. Audi^1,4^, Allen W. Cowley Jr.^2,3^, and Ranjan K. Dash^1,2,3,4^

^1^Department of Biomedical Engineering, Medical College of Wisconsin, Milwaukee, WI-53226, USA.

^2^Department of Physiology, Medical College of Wisconsin, Milwaukee, WI-53226, USA.

^3^Cardiovascular Research Center, Medical College of Wisconsin, Milwaukee, WI-53226, USA.

^4^Department of Biomedical Engineering, Marquette University, Milwaukee, WI-53223, USA.

**Running Head:** Tissue-Specific and Substrate-Dependent Mitochondrial Respiration an d Bioenergetics

**Address for correspondence:**

| Ranjan K. Dash, Ph.D.  Department of Biomedical Engineering  Medical College of Wisconsin  8701 Watertown Plank Road  Milwaukee, WI-53226  Email: [rdash@mcw.edu](mailto:rdash@mcw.edu)  Phone: 414-955-4497 |  |
| --- | --- |

## Overview

This supplemental materials document consists of five parts. Part S1 lists all the definitions and general terminologies, biochemical species, and enzymatic and transport processes considered in this manuscript. Part S2 lists the flux expressions and the associated kinetic parameters for the enzymatic reactions. Part S3 lists the flux expressions and the associated kinetic parameters for the transport reactions. Part S4 lists the governing ordinary differential equations (ODEs) based on mass balances of the biochemical species in different regions of the integrated mitochondrial model. Part S4 also includes the initial conditions for the biochemical species for solving the associated ODES. Part S5 includes additional model simulation results not included in the main manuscript.

## Part S1: Definitions

**Table S1.1: Glossary of general terminologies, biochemical species, enzymes, and transporters**

| **Definitions & Subscripts** | **Description** |
| --- | --- |
| Notation *C_X_* | Concentration of a biochemical species, X |
| Notation *J_R_* | Flux of a metabolic reaction, R |
| Notation *T_T_* | Flux of a metabolite transporter, T |
| Subscript m | Mitochondria matrix region |
| Subscript e | Extra-mitochondria (buffer) region |
| Subscript i | Inter-membrane space (IMS) region |
| **General Terminologies** | **Description** |
| AM | Alpha-ketoglutarate + Malate |
| ETC | Electron transport chain |
| FET | Forward electron transport |
| GM | Glutamate + Malate |
| IMM | Inner mitochondrial membrane |
| IMS | Inter-membrane space |
| mTAL | Medullary thick ascending limbs of loop of Henle |
| O2k | Oxygraph-2k |
| OCR | Oxygen consumption rate |
| OM | Outer medulla |
| OMM | Outer mitochondrial membrane |
| OxPhos | Oxidative phosphorylation |
| PCT | Proximal convoluted tubules |
| PT | Proximal tubules |
| PM | Pyruvate + Malate |
| R123 | Rhodamine-123 dye |
| RCI | Respiratory control index (state 3 OCR/state 2 OCR) |
| RET | Reverse electron transport |
| ROS | Reactive oxygen species |
| ROT | Rotenone |
| SD | Sprague-Dawley |
| TCA | Tricarboxylic acid cycle |
| ΔΨ | Membrane potential |
| **Biochemical Species** | **Description** |
| ACoA | Acetyl-coenzyme A |
| ADP | Adenosine diphosphate |
| ATP | Adenosine triphosphate |
| AKG | Alpha-ketoglutarate (2-oxoglutarate) |
| ASP | Aspartate |
| CoA | Coenzyme A |
| CIT | Citrate |
| CO_2_ | Carbon dioxide |
| CytCo | Oxidized form of cytochrome c |
| CytCr | Reduced form of cytochrome c |
| FAD | Oxidized form of flavin adenine dinucleotide |
| FADH_2_ | Reduced form of flavin adenine dinucleotide |
| FUM | Fumarate |
| H^+^ | Hydrogen ions (protons) |
| HCO_3_^-^ | Bicarbonate ions |
| GDP | Guanidine diphosphate |
| GTP | Guanidine triphosphate |
| GLU | Glutamate |
| ICIT | Isocitrate |
| MAL | Malate |
| NAD | Oxidized nicotinamide adenine dinucleotide |
| NADH | Reduced nicotinamide adenine dinucleotide |
| O_2_ | Oxygen |
| OXA | Oxaloacetate |
| Pi | Inorganic phosphate |
| PYR | Pyruvate |
| SCoA | Succinyl-coenzyme A |
| SUC | Succinate |
| UQ | Oxidized form of ubiquinone |
| UQH_2_ | Reduced form of ubiquinone |
| **Enzymes** | **Description** |
| AKGDH | α-ketoglutarate dehydrogenase |
| CITS | Citrate synthase |
| ICDH | Lumped reaction of aconitase and isocitrate dehydrogenase |
| CI | Complex I |
| CII | Complex II |
| CIII | Complex III |
| CIV | Complex IV |
| CV | Complex V |
| FH | Fumarate hydratase |
| GOT | Glutamic-oxaloacetic transaminase |
| MDH | Malate dehydrogenase |
| NDK | Nucleoside diphosphokinase |
| PDH | Pyruvate dehydrogenase |
| SCAS | Succinyl-coenzyme A synthetase |
| SDH | Succinate dehydrogenase |
| **Transporters** | **Description** |
| ANT | Adenine nucleotide translocase |
| DCCM | Dicarboxylate carrier for malate-Pi exchanger |
| DCCS | Dicarboxylate carrier for succinate-Pi exchanger |
| GAE | Glutamate-aspartate exchanger |
| GLUH | Glutamate-hydrogen co-transporter |
| Hleak | Passive proton leak from inter-membrane space to mitochondria matrix |
| OME | α-ketoglutarate (2-oxoglutarate) malate exchanger |
| PIC | Inorganic phosphate carrier |
| PYRH | Pyruvate-hydrogen co-transporter |
| TCC | Tricarboxylate carrier |

**Table S1.2: Enzymatic reactions in the integrated mitochondrial model**

| Reaction Number | Enzyme Name | Enzyme Reaction |
| --- | --- | --- |
| 1 | PDH | *PYR_m_+CoA_m_+NAD_m_ ⇌ ACoA_m_+CO_2_+NADH_m_+H_m_* |
| 2 | CITS | *ACoA_m_+OXA_m_ ⇌ CIT_m_+CoA_m_+2H_m_* |
| 3 | ACON+ICDH | *CIT_m_+NAD_m_ ⇌ AKG_m_+NADH_m_+CO_2_+2H_m_* |
| 4 | AKGDH | *AKG_m_+CoA_m_+NAD_m_ ⇌ SCoA_m_+NADH_m_+CO_2_+H_m_* |
| 5 | GOT | *ASP_m_+AKG_m_ ⇌ GLU_m_+OXA_m_* |
| 6 | SCAS | *SCoA_m_+GDP_m_+Pi_m_ ⇌ SUC_m_+GTP_m_+CoA_m_+H_m_* |
| 7 | NDK | *GTP_m_+ADP_m_ ⇌ GDP_m_+ATP_m_* |
| 8 | SDH+CII | *SUC_m_+UQ_m_⇌ FUM_m_+UQH_2m_* |
| 9 | FH | *FUM_m_ ⇌ MAL_m_* |
| 10 | MDH | *MAL_m_+NAD_m_ ⇌ OXA_m_+NADH_m_+H_m_* |
| 11 | CI | *NADH_m_+UQ_m_+H_m_ ⇌ NAD_m_+UQH_2m_+4∆H* |
| 12 | CIII | *UQH_2m_+2CytCo ⇌ UQ_m_+2CytCr+2$H_{m}$+4∆H* |
| 13 | CIV | *2CytCr+0.5O_2_+2H_m_ ⇌ 2CytCo+H_2_O+2∆H* |
| 14 | CV | *ADP_m_+Pi_m_+H_m_+3∆H ⇌ ATP_m_* |

Table S1.3: Metabolite transporters in the integrated mitochondrial model

| Transporter  Number | Transporter  Name | Transporter Reaction |
| --- | --- | --- |
| 1 | PYRH | *PYR_e_ + H_e_ ⇌ PYR_m_ + H_m_* |
| 2 | GLUH | *GLU_e_+ H_e_ ⇌ GLU_m_+ H_m_* |
| 3 | DCCM (MAL) | *MAL_e_+Pi_m_ ⇌MAL_m_+Pi_e_* |
| 4 | DCCS (SUC) | *SUC_e_+Pi_m_ ⇌ SUC_m_+Pi_e_* |
| 5 | TCC | *MAL_e_+ HCIT_m_ ⇌ MAL_m_+ HCIT_e_* |
| 6 | OME | *MAL_e_+AKG_m_ ⇌ MAL_m_+AKG_e_* |
| 7 | GAE | *HGLU_e_+ ASP_m_ ⇌ HGLU_m_ + ASP_e_* |
| 8 | ANT | *ADP_e_ + ATP_m_ ⇌ ADP_m_ + ATP_e_* |
| 9 | PIC | *Pi_e_ + H_e_ ⇌ Pi_m_ + H_m_* |
| 10 | Hleak | *H_e_ ⇌ H_m_* |

## Part S2: Enzymatic reactions and flux expressions

For the flux expressions of all the TCA cycle reactions described below, CO_2_ is considered as total CO_2_ (free or dissolved CO_2_ plus bicarbonate ions (HCO_3_^-^) in the mitochondrial matrix water space), which is usually considered negligible for an isolated mitochondrial experimental system.

$$C_{CO_{2},tot}=C_{CO_{2}}+C_{HCO_{3}^{-}}=\left(1+\frac{K_{CO2Hyd}K_{H2CO3}}{C_{H^{+}}}\right)C_{CO_{2}}$$

where *K_CO2Hyd_* = 2.7E-3 (unitless) is the equilibrium constant for the CO_2_ hydration reaction (CO_2_ + H_2_O *⇌* H_2_CO_3_) and *K_H2CO3_* = 2.4E-4 Molar is the equilibrium constant for the H_2_CO_3_ dissociation reaction (H_2_CO_3_ *⇌* HCO_3_^-^ + H^+^). H_2_CO_3_ and CO_3_^2-^ are usually negligible under physiological conditions and hence are ignored. The apparent equilibrium constants and transformed Gibb’s free energy of the CO_2_ producing reactions are based on total CO_2_, *C_CO2,tot_*, in the mitochondrial matrix water space.

Unless otherwise indicated, all kinetic parameters are taken from our previous lung tissue mitochondrial bioenergetics models^1,2^. The transformed Gibbs free energy of the reactions, which are taken from our thermodynamic database^3,4^, are derived from the thermodynamics database of Alberty ^5^. The heights of the free energy barriers (β) associated with mitochondrial electron transport chain (ETC) and F1F0-ATPase reactions (Complexes I-V) are modified from our previous models^1,2^ to account for the experimentally-observed mitochondrial membrane potential (ΔΨ) dependencies of corresponding reaction fluxes.

### Reaction 1: Pyruvate Dehydrogenase (PDH)

PYR_m_ + CoA_m_ + NAD_m_ ⇌ ACoA_m_ + CO_2_ + NADH_m_ + H_m_^+^

For this reaction, the reactants and products are denoted as $A=PYR_{m}$, $B=CoA_{m}$, $C=NAD_{m}$, $D=ACoA_{m}$, $E=NADH_{m}$, and $F=CO_{2}$. The participating co-factor pairs are *NADH_m_* & *NAD_m_* and *CoA_m_* & *ACoA_m_*. Hence, the overall reaction flux equation, $J_{PDH}$, is given by:

$$J_{PDH}=\frac{\frac{V_{maxf,PDH}}{K_{A}K_{B}K_{C}}\left(C_{A}C_{B}C_{C}-\frac{C_{D}C_{E}C_{F}}{K'_{eq,PDH}}\right)}{\left(1+\frac{C_{A}}{K_{A}}\right)\left(1+\frac{C_{B}}{K_{B}}+\frac{C_{D}}{K_{D}}\right)\left(1+\frac{C_{C}}{K_{C}}+\frac{C_{E}}{K_{E}}\right)\left(1+\frac{C_{F}}{K_{F}}\right)}$$

The PDH-catalyzed reaction involves the generation of one proton. Thus, the pH-dependent apparent equilibrium constant for this reaction is defined as:

$$K'_{eq,PDH}=\text{exp}\left(\frac{-\Delta_{r} G'^{0}_{PDH}}{RT}\right)\times10^{pH_{m} -7}$$

where $pH_{m}$ is *pH* in the mitochondrial matrix and $\Delta_{r}{G^{'}}_{PDH}^{0}$ is the transformed standard Gibbs free energy of the reaction catalyzed by *PDH* at *pH* = 7.

Table S2.1: PDH model kinetic parameters

| **Parameter** | **Definition** | **Value** |
| --- | --- | --- |
| $V_{maxf,PDH}$ | Maximum forward reaction rate | Tissue-specific |
| $K_{A}$ | PYR binding constant | $5.1\times10^{-3} M$ |
| $K_{B}$ | CoA binding constant | $14.9\times10^{-6} M$ |
| $K_{C}$ | NAD binding constant | $35.0\times10^{-6} M$ |
| $K_{D}$ | ACoA binding constant | $14.9\times10^{-6} M$ |
| $K_{E}$ | NADH binding constant | $35.0\times10^{-6} M$ |
| *K_F_* | CO_2_ binding constant | $10\times10^{-3} M$ |
| $\Delta_{r}{G^{'}}_{PDH}^{0}$ | Gibbs free energy of the reaction (pH=7) | -39.26 kJ/mol |

### Reaction 2: Citrate Synthase (CITS)

ACoA_m_ + OXA_m_ ⇌ CoA_m_ + CIT_m_ +2H_m_^+^

For this reaction, the reactants and products are denoted as $A=ACoA_{m}$, $B=OXA_{m}$, $C=CoA_{m}$, and $D=CIT_{m}$, whereas *CoA_m_* & *ACoA_m_* is a co-factor pair. Thus, the overall reaction flux equation, $J_{CITS}$, is given by:

$$J_{CITS}=\frac{\frac{V_{maxf,CITS}}{K_{A}K_{B}}\left(C_{A}C_{B}-\frac{C_{C}C_{D}}{K'_{eq,CITS}}\right)}{\left(1+\frac{C_{A}}{K_{A}}+\frac{C_{C}}{K_{C}}\right)\left(1+\frac{C_{B}}{K_{B}}\right)\left(1+\frac{C_{D}}{K_{D}}\right)}$$

The reaction catalyzed by *CITS* generates two protons. Thus, the pH-dependent apparent equilibrium constant for this reaction is defined as:

$$K'_{eq,CITS}=\text{exp}\left(\frac{-\Delta_{r}G'_{CITS}^{0}}{RT}\right)\times10^{2(pH_{m} -7)}$$

Table S2.2: CITS model kinetic parameters

| **Parameter** | **Definition** | **Value** |
| --- | --- | --- |
| $V_{maxf,CITS}$ | Maximum forward reaction rate | Tissue-specific |
| *K_A_* | ACoA binding constant | $3.9\times10^{-6} M$ |
| *K_B_* | OXA binding constant | $4.53\times10^{-6} M$ |
| *K_C_* | CoA binding constant | $57.9\times10^{-6} M$ |
| *K_D_* | CIT binding constant | $4.3\times10^{-3} M$ |
| $\Delta_{r}{G^{'}}_{CITS}^{0}$ | Gibbs free energy of the reaction (pH=7) | -36.61 kJ/mol |

### Reaction 3: Aconitase and Isocitrate Dehydrogenase (ICDH)

The reactions catalyzed by aconitase and isocitrate dehydrogenase (ICDH) are:

***Aconitase:*** *CIT_m_ ⇌ ISOCITm + H_2_O*

***Isocitrate Dehydrogenase:*** *ISOCIT_m_ +NAD_m_ + H_2_O ⇌ AKG_m_ + NADH_m_ + CO_2_ + 2H_m_^+^*

Under the assumption that the reaction catalyzed by aconitase is rapidly equilibrating, the reactions catalyzed by aconitase and ICDH can be lumped into the following reaction:

CIT_m_ +NAD_m_ ⇌ AKG_m_ + NADH_m_ +CO_2_ + 2H_m_^+^

For this reaction, the reactants and products are denoted as $A=CIT_{m}$, $B=NAD_{m}$, $C=AKG_{m}$, $D=NADH_{m}$, and $E=CO_{2}$, whereas the co-factor pair is *NAD_m_* & *NADH_m_*. In addition, *NADH_m_* inhibits ICDH reaction. This effect is modeled by adding an inhibitory factor dependent on *NADH* concentration^6^:

$$Inh_{NADH}=10^{3}/\left(1+\frac{C_{D}}{K_{D}}\right)$$

The overall reaction flux equation, $J_{ICDH}$, is given by:

$$J_{ICDH}=\frac{\frac{V_{maxf,ICDH}}{K_{A}K_{B}}Inh_{NADH}\left(C_{A}C_{B}-\frac{C_{C}C_{D}C_{E}}{K'_{eq,ICDH}}\right)}{\left(1+\frac{C_{A}}{K_{A}}\right)\left(1+\frac{C_{C}}{K_{C}}\right)\left(1+\frac{C_{B}}{K_{B}}+\frac{C_{D}}{K_{D}}\right)\left(1+\frac{C_{E}}{K_{E}}\right)}$$

This lumped reaction results in the generation of two protons. Thus, the equilibrium constant for this reaction is defined as:

$$K'_{eq,ICDH}=\text{exp}\left(\frac{-\text{Δ}_{r}G'^{0}_{ICDH}}{RT}\right)\times10^{2(pH_{m} -7)}$$

**Table S2.3: ICDH model kinetic parameters**

| **Parameter** | **Definition** | **Value** |
| --- | --- | --- |
| $V_{maxf,ICDH}$ | Maximum forward reaction rate | Tissue-specific |
| *K_A_* | CIT binding constant | $1.3\times10^{-3} M$ |
| *K_B_* | NAD binding constant | $500\times10^{-6} M$ |
| *K_C_* | AKG binding constant | $3.5\times10^{-6} M$ |
| *K_D_* | NADH binding constant | $4.7\times10^{-6} M$ |
| *K_E_* | CO_2_ binding constant | $10\times10^{-3} M$ |
| $\Delta_{r}{G^{'0}}_{ICDH}$ | Gibbs free energy of the reaction (pH=7) | 3.29 KJ/mol |

### Reaction 4: AKG dehydrogenase (AKGDH)

AKG_m_ + CoA_m_ + NAD_m_ ⇌ SCoA_m_+NADH_m_+CO_2_ + H^+^

For this reaction, the reactants and products are denoted as $A=AKG_{m}$, $B=CoA_{m}$, $C=NAD_{m}$, $D=SCoA_{m}$, $E=NADH_{m}$, and $F=CO_{2}$, whereas the co-factor pairs are *CoA_m_* & *ACoA_m_* and *NADH_m_* & *NAD_m_*. Thus, the overall reaction flux equation, $J_{AKGDH}$, is given by:

$$J_{AKGDH}=\frac{\frac{V_{maxf,AKGDH}}{K_{A}K_{B}K_{C}}\left(C_{A}C_{B}C_{C}-\frac{C_{D}C_{E}C_{F}}{K'_{eq,AKGDH}}\right)}{\left(1+\frac{C_{C}}{K_{C}}+\frac{C_{E}}{K_{E}}\right)\left(1+\frac{C_{B}}{K_{B}}+\frac{C_{D}}{K_{D}}\right)\left(1+\frac{C_{A}}{K_{A}}\right)\left(1+\frac{C_{F}}{K_{F}}\right)}$$

Since this reaction results in the generation of one proton, the equilibrium constant for this reaction is defined as:

$$K'_{eq,AKGDH}=\exp\left(\frac{-\Delta_{r}G'_{AKGDH}^{0}}{RT}\right)\times10^{pH_{m}-7}$$

**Table S2.4: AKGDH model kinetic parameters**

| **Parameter** | **Definition** | **Value** |
| --- | --- | --- |
| $V_{maxf,AKDGH}$ | Maximum forward reaction rate | Tissue-specific |
| *K_A_* | AKG binding constant | $85.9\times10^{-6} M$ |
| *K_B_* | CoA binding constant | $1.63\times10^{-6} M$ |
| *K_C_* | NAD binding constant | $46.6\times10^{-6} M$ |
| *K_D_* | SCoA binding constant | $1.63\times10^{-6} M$ |
| *K_E_* | NADH binding constant | $7.14\times10^{-6} M$ |
| *K_F_* | CO_2_ binding constant | $10\times10^{-3} M$ |
| $\Delta_{r}{G^{'}}_{AKGDH}^{0}$ | Gibbs free energy of the reaction (pH=7) | -37.08 KJ/mol |

**Reaction 5: Glutamate oxaloacetate transaminase (GOT)**

ASP_m_ + AKG_m_ ⇌ GLU_m_ + OXA_m_

For this reaction, the reactants and products are denoted as $A=ASP_{m}$, $B=AKG_{m}$, $C=GLU_{m}$, $D=OXA_{m}$, and the overall reaction flux equation, $J_{GOT}$, is given by:

$$J_{GOT}=\frac{\frac{V_{maxf,GOT}}{K_{A}K_{B}}\left(C_{A}C_{B}-\frac{C_{C}C_{D}}{K'_{eq,GOT}}\right)}{\left(1+\frac{C_{A}}{K_{A}}\right)\left(1+\frac{C_{B}}{K_{B}}\right)\left(1+\frac{C_{C}}{K_{C}}\right)\left(1+\frac{C_{D}}{K_{D}}\right)}$$

The equilibrium constant for this reaction is:

$$K'_{eq,GOT}=\text{exp}\left(\frac{-\Delta_{r} G'^{0}_{GOT}}{RT}\right)$$

Table S2.5: GOT model kinetic parameters

| **Parameters** | **Definition** | **Value** |
| --- | --- | --- |
| $V_{maxf,GOT}$ | Maximum forward reaction rate | Tissue-specific |
| *K_A_* | ASP binding constant | $3.9\times10^{-3} M$ |
| *K_B_* | ΑKG binding constant | $430\times10^{-6} M$ |
| *K_C_* | GLU binding constant | $8.9\times10^{-3} M$ |
| *K_D_* | OXA binding constant | $88\times10^{-6} M$ |
| $\Delta_{r} G'^{0}_{GOT}$ | Gibbs free energy of the reaction (pH=7) | -1.31KJ/mol |

**Reaction 6: SCoA synthetase (SCAS)**

SCoA_m_ + GDP_m_ + Pi_m_ ⇌ SUC_m_ + GTP_m_ + CoA_m_ + H_m_^+^

For this reaction, the reactants and products are denoted as $A=SCoA_{m}$, $B=GDP_{m}$, $C=Pi_{m}$, $D=SUC_{m}$, $E=GTP_{m}$, and $F=CoA_{m}$, whereas the participating co-factor pairs are *SCoA_m_* & *CoA_m_, SCoA_m_* & *SUC_m_*, *GDP_m_* & *GTP_m_,* and G*TP_m_* & *Pi_m_.* Therefore, the overall reaction flux equation, $J_{SCAS}$, is given by:

$$J_{SCAS}=\frac{\frac{V_{maxf,SCAS}}{K_{A}K_{B}K_{C}}\left(C_{A}C_{B}C_{C}-\frac{C_{D}C_{E}C_{F}}{K'_{eq,SCAS}}\right)}{\left(1+\frac{C_{A}}{K_{A}}+\frac{C_{D}}{K_{D}}+\frac{C_{F}}{K_{F}}+\frac{C_{D}C_{F}}{K_{D}K_{F}}\right)\left(1+\frac{C_{B}}{K_{B}}+\frac{C_{C}}{K_{C}}+\frac{C_{E}}{K_{E}}+\frac{C_{B}C_{C}}{K_{B}K_{C}}\right)}$$

This reaction involves the generation of one proton. Thus, the equilibrium constant for this reaction is defined as:

$$K'_{eq,SCAS}=\text{exp}\left(\frac{-\Delta_{r} G'^{0}_{SCAS}}{RT}\right)\times10^{pH_{m} -7}$$

Table S2.6: SCAS model kinetic parameters

| **Parameter** | **Definition** | **Value** |
| --- | --- | --- |
| $V_{maxf,SCAS}$ | Maximum forward reaction rate | Tissue-specific |
| *K_A_* | SCoA binding constant | $12.5\times10^{-6} M$ |
| *K_B_* | GDP binding constant | $5.70\times10^{-6} M$ |
| *K_C_* | Pi binding constant | $2.10\times10^{-3} M$ |
| *K_D_* | SUC binding constant | $451\times10^{-6} M$ |
| *K_E_* | GTP binding constant | $23.1\times10^{-6} M$ |
| *K_F_* | CoA binding constant | $17.7\times10^{-6} M$ |
| $\Delta_{r} G'^{0}_{SCAS}$ | Gibbs free energy of the reaction (pH=7) | 1.26 KJ/mol |

**Reaction 7: Nucleoside diphosphokinase (NDK)**

*GTP_m_+ADP_m_ ⇌ GDP_m_+ATP_m_*

For this reaction, the reactants and products are denoted as $A=GTP_{m}$, $B=ADP_{m}$, $C=GDP_{m}$ and $D=ATP_{m}$, whereas the participating co-factor pairs are *GTP_m_* & *GDP_m_* and *ADP_m_* & *ATP_m_*. Therefore, the overall reaction flux equation, $J_{NDK}$, is given by:

$$J_{NDK}=\frac{\frac{V_{maxf,NDK}}{K_{A}K_{B}}\left(C_{A}C_{B}-\frac{C_{C}C_{D}}{K'_{eq,NDK}}\right)}{\left(1+\frac{C_{A}}{K_{A}}+\frac{C_{C}}{K_{C}}\right)\left(1+\frac{C_{B}}{K_{B}}+\frac{C_{D}}{K_{D}}\right)}$$

where

$$K'_{eq,NDK}=\text{exp}\left(\frac{-\Delta_{r} G'^{0}_{NDK}}{RT}\right)$$

Table S2.7: NDK model kinetic parameters

| **Parameter** | **Definition** | **Value** |
| --- | --- | --- |
| $V_{maxf,NDK}$ | Maximum forward reaction rate | Tissue-specific |
| *K_A_* | GTP binding constant | $111\times10^{-6}$ M |
| *K_B_* | ADP binding constant | $100\times10^{-6}$ M |
| *K_C_* | GDP binding constant | $260\times10^{-6}$ M |
| *K_D_* | ATP binding constant | $278\times10^{-6}$ M |
| $\Delta_{r} G'^{0}_{NDK}$ | Gibbs free energy of the reaction (pH=7) | -0.56 KJ/mol |

**Reaction 8: Succinate dehydrogenase | Complex II (SDH | CII)**

The electron transfer reactions catalyzed by SDH and CII are given by:

*SUC_m_ + FAD_m_ ⇌ FUM_m_ + FADH_2,m_*

*FADH_2,m_ + UQ_m_ ⇌ FAD_m_ + UQH_2,m_*

These two reactions can be lumped to generate the following lumped reaction:

*SUC_m_ + UQ_m_ ⇌ FUM_m_ + UQH_2,m_*

For this reaction, the reactants and products are denoted as $A=SUCm$, $B=UQ_{m}$, $C=FUM_{m}$, and $D=UQH_{2,m}$, whereas the participating co-factor pairs are *SUC_m_* & *FUM_m_* and *UQ_m_* & *UQH_2,m_*. SDH is known to be inhibited by OXA_m_ accumulation and stimulated by MAL_m_ accumulation in the mitochondrial matrix, which are modeled with the following modifications of the succinate binding constant:

$$K'_{A}=K_{A}\left(1+\frac{C_{OXAm}}{K_{OXA}}\right)$$

$$K''_{A}=K'_{A}/\left(1+\frac{C_{MALm}}{K_{MAL}}\right)$$

Thus, the overall reaction flux equation, $J_{SDH}$, is given by:

$$J_{SDH}=\frac{\frac{V_{maxf,SDH}}{K''_{A}K_{B}}\left(C_{A}C_{B}-\frac{C_{C}C_{D}}{K'_{eq,SDH}}\right)}{\left(1+\frac{C_{A}}{K''_{A}}+\frac{C_{C}}{K_{C}}\right)\left(1+\frac{C_{B}}{K_{B}}+\frac{C_{D}}{K_{D}}\right)}$$

where

$$K'_{eq,SDH}=\text{exp}\left(\frac{-\Delta_{r} G'^{0}_{SDH}}{RT}\right)$$

Table S2.8: SDH model kinetic parameters

| **Parameters** | **Definition** | **Value** |
| --- | --- | --- |
| $V_{maxf,SDH}$ | Maximum forward reaction rate | Tissue-specific |
| *K_A_* | SUC binding constant | $1.80\times10^{-3} M$ |
| *K_B_* | UQ binding constant | $140\times10^{-6} M$ |
| *K_C_* | FUM binding constant | $1.8\times10^{-3} M$ |
| *K_D_* | UQH_2_ binding constant | $2.45\times10^{-6} M$ |
| $K_{OXA}$ | OXA binding constant | $0.0315\times10^{-6} M$ |
| $K_{MAL}$ | MAL binding constant | $11.7\times10^{-6} M$ |
| $\Delta_{r} G'^{0}_{SDH}$ | Gibbs free energy of the reaction (pH=7) | -2.41 KJ/mol |

**Reaction 9: Fumarate Hydratase (FH)**

FUM_m_ ⇌ MAL_m_

In this reaction, the reactants and products are denoted as $A=FUM_{m}$ and $B=MAL_{m}$, and the flux equation, $J_{FH}$, is given by:

$$J_{FH}=\frac{\frac{V_{maxf,FH}}{K_{A}K_{B}}\left(C_{A}-\frac{C_{B}}{K'_{eq,FH}}\right)}{\left(1+\frac{C_{A}}{K_{A}}\right)\left(1+\frac{C_{B}}{K_{B}}\right)}$$

where

$$K'_{eq,FH}=\text{exp}\left(\frac{-\Delta_{r} G'^{0}_{FH}}{RT}\right)$$

Table S2.9: FH model kinetic parameters

| **Parameter** | **Definition** | **Value** |
| --- | --- | --- |
| $V_{maxf,FH}$ | Maximum forward reaction rate | Tissue-specific |
| *K_A_* | FUM binding constant | $1.8\times10^{-3} M$ |
| *K_B_* | MAL binding constant | $1.8\times10^{-3} M$ |
| $\Delta_{r} G'^{0}_{FH}$ | Gibbs free energy of the reaction (pH=7) | -3.6 KJ/mol |

**Reaction 10: Malate Dehydrogenase (MDH)**

MAL_m_ + NAD_m_ ⇌ OXA_m_ + NADH_m_ + H_m_^+^

For this reaction, the reactants and products are denoted as $A=MAL_{m}$, $B=NAD_{m}$, $C=OXA_{m}$ and $D=NADH_{m}$, and the participating co-factor pair is *NADH_m_* & *NAD_m_*. In addition, *MAL_m_* is known to be a competitive inhibitor of *OXA_m_*^7^. Thus, the overall reaction flux equation, $J_{MDH}$, is given by:

$$J_{MDH}=\frac{\frac{V_{maxf,MDH}}{K_{A}K_{B}}\left(AB-\frac{CD}{K'_{eq,MDH}}\right)}{\left(1+\frac{A}{K_{A}}+\frac{C}{K_{C}}\right)\left(1+\frac{B}{K_{B}}+\frac{D}{K_{D}}\right)}$$

The above reaction involves the generation of one proton. Thus, the equilibrium constant for this reaction is defined as:

$$K'_{eq,MDH}=\text{exp}\left(\frac{-\Delta_{r} G'^{0}_{MDH}}{RT}\right)\times10^{pH_{m} -7}$$

**Table S2.10: MDH model kinetic parameters**

| **Parameter** | **Definition** | **Value** |
| --- | --- | --- |
| $V_{maxf,MDH}$ | Maximum forward reaction rate | Tissue-specific |
| *K_A_* | MAL binding constant | $155\times10^{-6} M$ |
| *K_B_* | NAD binding constant | $1.10\times10^{-3} M$ |
| *K_C_* | OXA binding constant | $3.38\times10^{-6} M$ |
| *K_D_* | NADH binding constant | $36.1\times10^{-6} M$ |
| $\Delta_{r} G'^{0}_{MDH}$ | Gibbs free energy of the reaction (pH=7) | 28.83 KJ/mol |

**Reaction 11: Complex I (CI)**

NADH_m_ + UQ_m_ + H_m_ ⇌ NAD_m_ + UQH_2m_ + 4ΔH

The reaction catalyzed by complex I involves pumping of four protons from the mitochondrial matrix into the inter-membrane space. Thus, the reaction flux is dependent on the proton motive force, defined as $\Delta G_{H}=F\Delta\Psi_{m}+\left(RT\right)ln\left(\left[H_{i}^{+}\left]/\right[H_{m}^{+}\right]\right)$, where *F* is the Faraday’s constant and $\Delta\Psi_{m}$ is the mitochondrial membrane potential. To take this dependency into account, the kinetic parameters was modified to be dependent on membrane potential^8^. For this reaction, the reactants and products are denoted as $A=NADH_{m}$, $B=UQ_{m}$, $C=NAD_{m}$, and $D=UQH_{2m}$, and the participating co-factor pairs are *NAD_m_* & *NADH_m_* and *UQ_m_* & *UQH_2m_*. Thus, the overall reaction flux equation, $J_{CI}$, is given by:

$$J_{CI}=\frac{\frac{V_{maxf,CI}}{K_{A}K_{B}}\left\{\begin{matrix} \exp\left(\frac{-\beta_{CI}\left(\Delta_{r}G'^{0}_{CI}+4\Delta G_{H}-RT\ln(C_{H}/10^{-7})\right)}{RT}\right)C_{A}C_{B} \\ -\exp\left(\frac{-\left(\beta_{CI}-1\right)\left(\Delta_{r}G'^{0}_{CI}+4\Delta G_{H}-RT\ln(C_{H}/10^{-7})\right)}{RT}\right)C_{C}C_{D} \end{matrix}\right\}}{\left(1+\frac{C_{A}}{K_{A}}+\frac{C_{C}}{K_{C}}\right)\left(1+\frac{C_{B}}{K_{B}}+\frac{C_{D}}{K_{D}}\right)}$$

Table S2.11: CI model kinetic parameters

| **Parameter** | **Definition** | **Value** |
| --- | --- | --- |
| $V_{maxf,CI}$ | Maximum forward reaction rate | Tissue-specific |
| *K_A_* | NADH binding constant | $1.50\times10^{-6} M$ |
| *K_B_* | UQ binding constant | $58.1\times10^{-6} M$ |
| *K_C_* | NAD binding constant | $428\times10^{-6} M$ |
| *K_D_* | UQH_2_ binding constant | $520\times10^{-6} M$ |
| *β_CI_* | Complex I free energy barrier | 0.35 |
| $\Delta_{r}{G^{'0}}_{CI}$ | Gibbs free energy of the reaction (pH=7) | -69.37 KJ/mol |

**Reaction 12: Complex III (CIII)**

UQH_2,m_ + 2CytC_o_ ⇌ UQ_m_ + 2CytC_r_ + 2H_m_ + 4ΔH

For this reaction, the reactants and products are denoted as $A=UQH_{2,m}$, $B=CytCo$, $C=UQ_{m}$, and $D=CytCr$, and the participating co-factor pairs are *UQ_m_* & *UQH_2,m_* and *CytC_r_* & *CytC_o_*. The reaction catalyzed by complex III involves pumping four protons from mitochondria matrix into inter-membrane space. Thus, the overall reaction flux equation, $J_{CIII}$, is given by:

$$J_{CIII}=\frac{\frac{V_{maxf,CIII}}{K_{A}K_{B}^{2}}\left(\begin{matrix} \exp\left\{-\beta_{CIII}\left(\frac{\Delta_{r}G'^{0}_{CIII}+4\Delta G_{H}+2RT\ln(C_{H}/10^{-7})-2F\Delta\Psi}{RT}\right)\right\}C_{A}C_{B}^{2} \\ -\exp\left\{-\left(\beta_{CIII}-1\right)\left(\frac{\Delta_{r}G'^{0}_{CIII}+4\Delta G_{H}+2RT\ln(C_{H}/10^{-7})-2F\Delta\Psi}{RT}\right)\right\}C_{C}C_{D}^{2} \end{matrix}\right)}{\left(1+\frac{C_{A}}{K_{A}}+\frac{C_{C}}{K_{C}}\right)\left(1+\frac{C_{B}^{2}}{K_{B}^{2}}+\frac{C_{D}^{2}}{K_{D}^{2}}\right)}$$

Table S2.12: CIII model kinetic parameters

| **Parameter** | **Definition** | **Value** |
| --- | --- | --- |
| $V_{maxf,CIII}$ | Maximum forward reaction rate | Tissue-specific |
| *K_A_* | UQH_2_ binding constant | $4.66\times10^{-6} M$ |
| *K_B_* | CytCo binding constant | $3.76\times10^{-6} M$ |
| *K_C_* | UQ binding constant | $4.08\times10^{-6} M$ |
| *K_D_* | CytCr binding constant | $4.91\times10^{-6} M$ |
| *β_CIII_* | Complex III free energy barrier | 0.35 |
| $\Delta_{r}{G^{'0}}_{CIII}$ | Gibbs free energy of the reaction (pH=7) | -32.53KJ/mol |

### Reaction 13: Complex IV (CIV)

2CytCr + 0.5O_2_ + 2H_m_ ⇌ 2CytCo + H_2_O + 2ΔH

For this reaction, the reactants and products are denoted as $A=CytCr$, $B=O_{2}$, and $C=CytCo$, and the participating co-factor pair is *CytCr* & *CytCo*. In addition, two protons are pumped from the matrix side to the inter-membrane space. Another two protons are consumed in the matrix side. The overall reaction flux equation, $J_{CIV}$, is given by:

$$J_{CIV}= \frac{\frac{V_{maxf,CIV}}{K_{A}^{2}K_{B}^{0.5}}\left(\begin{matrix} \exp\left\{-\beta_{IV}\left(\frac{\Delta_{r}G'^{0}_{CIV}+2\Delta G_{H}+2F\Delta\Psi-RT\ln(C_{H}/10^{-7})}{RT}\right)\right\}C_{A}^{2}C_{B}^{0.5} \\ -\exp\left\{-\left(\beta_{IV}-1\right)\left(\frac{\Delta_{r}G'^{0}_{CIV}+2\Delta G_{H}+2F\Delta\Psi-RT\ln(C_{H}/10^{-7})}{RT}\right)\right\}C_{C}^{2} \end{matrix}\right)}{\left(1+\frac{C_{A}^{2}}{K_{A}^{2}}+\frac{C_{C}^{2}}{K_{C}^{2}}\right)\left(1+\frac{C_{B}^{0.5}}{K_{B}^{0.5}}\right)}$$

**Table S2.13: CIV model kinetic parameters**

| **Parameters** | **Definition** | **Value** |
| --- | --- | --- |
| $V_{maxf,CIV}$ | Maximum forward reaction rate | Tissue-specific |
| *K_A_* | CytCr binding constant | $680\times10^{-6} M$ |
| *K_B_* | Oxygen binding constant | $5.4\times10^{-6} M$ |
| *K_C_* | CytCo binding constant | $79.2\times10^{-6} M$ |
| *β_CIV_* | Complex IV free energy barrier | 0.35 |
| $\Delta_{r}{G^{'0}}_{CIV}$ | Gibbs free energy of the reaction (pH=7) | -122.94KJ/mol |

**Reaction 14: Complex V (CV)**

ADP_m_ + Pi_m_+ H_m_^+^ + nHΔH ⇌ ATP_m_

For this reaction, the reactants and products are denoted as $A=ADP_{m}$, $B=Pi_{m}$, and $C=ATP_{m}$, whereas the participating co-factor pair is *ADP_m_* & *ATP_m_*. nH is H^+^ stoichiometry coefficient for F_1_F_0_-ATPase. Thus, the overall reaction flux equation, $J_{CV}$, is given by:

$$J_{CV}=\frac{\frac{V_{maxf,CV}}{K_{A}K_{B}}\left\{\begin{matrix} \exp\left(\frac{-\beta_{V}\left(\Delta_{r}G'^{0}_{CV}-nH\Delta G_{H}-RT\ln(C_{H}/10^{-7})\right)}{RT}\right)C_{A}C_{B} \\ -\exp\left(\frac{-\left(\beta_{V}-1\right)\left(\Delta_{r}G'^{0}_{CV}-nH\Delta G_{H}-RT\ln(C_{H}/10^{-7})\right)}{RT}\right)C_{C} \end{matrix}\right\}}{\left(1+\frac{C_{A}}{K_{A}}+\frac{C_{C}}{K_{C}}\right)\left(1+\frac{C_{B}}{K_{B}}\right)}$$

Table S2.14: CV model kinetic parameters

| **Parameter** | **Definition** | **Value** |
| --- | --- | --- |
| $V_{maxf,CV}$ | Maximum forward reaction rate | Tissue-specific |
| *K_A_* | *ADP* binding constant | $50\times10^{-6} M$ |
| *K_B_* | *Pi* binding constant | $3\times10^{-3} M$ |
| *K_C_* | *ATP* binding constant | $50\times10^{-6} M$ |
| $\beta_{CV}$ | Complex V free energy barrier | 0.35 |
| $\Delta_{r}{G^{'0}}_{CV}$ | Gibbs free energy of the reaction (pH=7) | 36.03 KJ/mol |

## Part S3: Metabolic Transport Fluxes

### Transport 1: Pyruvate-Hydrogen Co-transporter (PYRH)

PYR_e_ + H_e_^+^ ⇌ PYR_m_ + H_m_^+^

For this reaction, the reactants and products are denoted as $A=PYR_{e},B=H_{e}^{+},C=PYR_{m}$ and $D=H_{m}^{+}$. The corresponding flux equation, $J_{PYRH}$, is given by:

$$J_{PYRH}=\frac{T_{\max f,PYRH}\left(\frac{C_{A}C_{B}}{K_{A}K_{B}}-\frac{C_{C}C_{D}}{K_{C}K_{D}}\right)}{1+\frac{C_{A}}{K_{A}}+\frac{C_{B}}{K_{B}}+\frac{C_{C}}{K_{C}}+\frac{C_{D}}{K_{D}}+\frac{C_{A}C_{B}}{K_{A}K_{B}}+\frac{C_{C}C_{D}}{K_{C}K_{D}}}$$

Table S3.1: PYRH model kinetic parameters

| **Parameters** | **Definition** | **Value** |
| --- | --- | --- |
| $T_{maxf, PYRH}$ | Maximum forward transport rate | Tissue-specific |
| *K_A_, K_C_* | *PYR* binding constant | $0.24\times10^{-3} M$ |
| *K_B_, K_D_* | *H^+^* binding constant | $0.1\times10^{-6} M$ |

### Transport 2: Glutamate-Hydrogen Cotransporter (GLUH)

GLU_e_ + H_e_^+^ ⇌ GLU_m_ + H_m_^+^

For this reaction, the reactants and products are denoted as $A=GLU_{e},B=H_{e}^{+},C=GLU_{m}$ and $D=H_{m}^{+}$. The corresponding flux equation, $J_{GLUH}$, is given by:

$$J_{GLUH}=\frac{T_{\max f,GLUH}\left(\frac{C_{A}C_{B}}{K_{A}K_{B}}-\frac{C_{C}C_{D}}{K_{C}K_{D}}\right)}{1+\frac{C_{A}}{K_{A}}+\frac{C_{B}}{K_{B}}+\frac{C_{C}}{K_{C}}+\frac{C_{D}}{K_{D}}+\frac{C_{A}C_{B}}{K_{A}K_{B}}+\frac{C_{C}C_{D}}{K_{C}K_{D}}}$$

Table S3.2: GLUH model kinetic parameters

| **Parameter** | **Definition** | **Value** |
| --- | --- | --- |
| $T_{maxf, GLUH}$ | Maximum forward transport rate | Tissue-specific |
| *K_A_, K_C_* | *GLU* binding constant | $1.4\times10^{-3} M$ |
| *K_B_, K_D_* | Proton binding constant | $0.1\times10^{-6} M$ |

### Transport 3: Dicarboxylate Carrier (DCCS)

DCC (SUC): SUC_e_ + Pi_m_  ⇌  SUC_m_ +Pi_e_

For this reaction, the reactants and products are denoted as $A=SUC_{e}, B=Pi_{m}, C=SUC_{m},\mathrm{and}D=Pi_{e}$. MAL accumulation in the mitochondrial matrix inhibits exchange of SUC with Pi, which is modeled by the following modification of the Succinate binding constant:

$$K'_{A,C}=K_{A,C}\left(1+\frac{C_{MALm}}{K_{MAL}}\right)$$

Furthermore, MAL binding constant (affinity) is assumed to be different in the three tissues. The corresponding flux equation, $J_{DCCS}$ , is given by:

$$J_{DCCS}=\frac{T_{\max f,DCCS}\left(\frac{C_{A}C_{B}}{K'_{A}K_{B}}-\frac{C_{C}C_{D}}{K'_{C}K_{D}}\right)}{1+\frac{C_{A}}{K'_{A}}+\frac{C_{B}}{K_{B}}+\frac{C_{C}}{K'_{C}}+\frac{C_{D}}{K_{D}}+\frac{C_{A}C_{B}}{K'_{A}K_{B}}+\frac{C_{C}C_{D}}{K'_{C}K_{D}}}$$

Table S3.3: DCCS model kinetic parameters

| **Parameter** | **Definition** | **Value in heart model** | **Value in kidney cortex model** | **Value in kidney OM model** |
| --- | --- | --- | --- | --- |
| $T_{maxf, DCCS}$ | Maximum forward transport rate | Tissue-specific | Tissue-specific | Tissue-specific |
| *K_A_, K_C_* | *SUC* binding constant | $1\times10^{-3} M$ | $1\times10^{-3} M$ | $1\times10^{-3} M$ |
| *K_B_, K_D_* | *Pi* binding constant | $0.93\times10^{-4} M$ | $0.93\times10^{-4} M$ | $0.93\times10^{-4} M$ |
| *K_MAL_* | *MAL* binding constant | $4.17\times10^{-3} M$ | $1.66\times10^{-3} M$ | $0.417\times10^{-3} M$ |

### Transport 4: Dicarboxylate Carrier (DCCM)

*DCC (MAL): MAL_e_ + Pi_m_ ⇌ MAL_m_* +*Pi_e_*

For this reaction, the reactants and products are denoted as $A=MAL_{e}, B=Pi_{m}, C=MAL_{m}, \mathrm{and}D=Pi_{e}.$ SUC accumulation in the mitochondrial matrix inhibits exchange of MAL and Pi, which is modeled by the following modification of the MAL binding constant:

$$K'_{A,C}=K_{A,C}\left(1+\frac{C_{SUCm}}{K_{SUC}}\right)$$

In addition, SUC binding constant (affinity) is assumed to be different in the three tissues. The corresponding flux equation, $J_{DCCM}$ is given by:

$$J_{DCCM}=\frac{T_{\max f,DCCM}\left(\frac{C_{A}C_{B}}{K'_{A}K_{B}}-\frac{C_{C}C_{D}}{K'_{C}K_{D}}\right)}{1+\frac{C_{A}}{K'_{A}}+\frac{C_{B}}{K_{B}}+\frac{C_{C}}{K'_{C}}+\frac{C_{D}}{K_{D}}+\frac{C_{A}C_{B}}{K'_{A}K_{B}}+\frac{C_{C}C_{D}}{K'_{C}K_{D}}}$$

Table S3.4: DCCM model kinetic parameters

| **Parameter** | **Definition** | **Value in heart model** | **Value in kidney cortex model** | **Value in kidney OM model** |
| --- | --- | --- | --- | --- |
| $T_{\max f,DCCM}$ | Maximum forward transport rate | Tissue-specific | Tissue-specific | Tissue-specific |
| *K_A_, K_C_* | *MAL* binding constant | $1.17\times10^{-4} M$ | $1.17\times10^{-3} M$ | $1.17\times10^{-3} M$ |
| *K_B_, K_D_* | *Pi* binding constant | $0.93\times10^{-3} M$ | $0.93\times10^{-3} M$ | $0.93\times10^{-3} M$ |
| *K_SUC_* | *SUC* binding constant | $0.057\times10^{-3} M$ | $5.7\times10^{-3} M$ | $0.57\times10^{-3} M$ |

### Transport 5: Tricarboxylate Carrier (TCC)

TCC is non-electrogenic and only $HCIT^{2-}$ and $MAL^{2-}$ are accepted as transport species of TCC. One proton must bind to CIT for transport process to occur.

*HCIT_m_ + MAL_e_ ⇌ HCIT_e_ + MAL_m_*

Here, $A=HCIT_{m},B=MAL_{e},C=HCIT_{e}$ and $D=MAL_{m}$. The corresponding flux equation, $J_{TCC}$, for this antiporter is:

$$J_{TCC}=\frac{T_{\max f,TCC}\left(\frac{C_{A}C_{B}}{K_{A}K_{B}}-\frac{C_{C}C_{D}}{K_{C}K_{D}}\right)}{1+\frac{C_{A}}{K_{A}}+\frac{C_{B}}{K_{B}}+\frac{C_{C}}{K_{C}}+\frac{C_{D}}{K_{D}}+\frac{C_{A}C_{B}}{K_{A}K_{B}}+\frac{C_{C}C_{D}}{K_{C}K_{D}}}$$

**Table S3.5: TCC model kinetic parameters**

| **Parameter** | **Definition** | **Value** |
| --- | --- | --- |
| $T_{\max f,TCC}$ | Maximum forward transport rate | Tissue-specific |
| *K_A_, K_C_* | *CIT* binding constant | $1\times10^{-3}M$ |
| *K_B_, K_D_* | *MAL* binding constant | $0.25\times10^{-3} M$ |
| *K_H_* | Proton binding constant | $0.1\times10^{-6} M$ |

### Transport 6: AKG-MAL Exchanger (OME)

*AKG_m_ + MAL_e_ ⇌ AKG_e_ + MAL_m_*

For this reaction, the reactants and products are denoted as $A=AKG_{m},B=MAL_{e},C=AKG_{e}$, and $D=MAL_{m}$. The corresponding flux equation, $J_{OME}$, is given by:

$$J_{OME}=\frac{T_{\max f,OME}\left(\frac{C_{A}C_{B}}{K_{A}K_{B}}-\frac{C_{C}C_{D}}{K_{C}K_{D}}\right)}{1+\frac{C_{A}}{K_{A}}+\frac{C_{B}}{K_{B}}+\frac{C_{C}}{K_{C}}+\frac{C_{D}}{K_{D}}+\frac{C_{A}C_{B}}{K_{A}K_{B}}+\frac{C_{C}C_{D}}{K_{C}K_{D}}}$$

Table S3.6: OME model kinetic parameters

| **Parameter** | **Definition** | **Value** |
| --- | --- | --- |
| $T_{\max f,OME}$ | Maximum forward transport rate | Tissue-specific |
| *K_A_, K_C_* | *AKG* binding constant | $0.24\times10^{-3} M$ |
| *K_B_, K_D_* | *MAL* binding constant | $1\times10^{-3} M$ |
| *K_H_* | Proton binding constant | $0.1\times10^{-6} M$ |

### Transport 7: ASP-HGLU Exchanger (GAE)

*ASP_m_ + HGLU_e_ ⇌ ASP_e_ + HGLU_m._*

GAE is an electrogenic transporter for which reactants and products are denoted as $A=ASP_{m},$ $B=HGLU_{e},$ $C=ASP_{e},$ and $D=HGLU_{m}$. The corresponding flux equation, $J_{GAE}$, is given by:

$$J_{GAE}=\frac{T_{\max f,GAE}\left(\exp\left(\frac{0.5F\Delta\psi}{RT}\right)\frac{C_{A}C_{B}}{K_{A}K_{B}}-\exp\left(-\frac{0.5F\Delta\psi}{RT}\right)\frac{C_{C}C_{D}}{K_{C}K_{D}}\right)}{1+\frac{C_{A}}{K_{A}}+\frac{C_{B}}{K_{B}}+\frac{C_{C}}{K_{C}}+\frac{C_{D}}{K_{D}}+\frac{C_{A}C_{B}}{K_{A}K_{B}}+\frac{C_{C}C_{D}}{K_{C}K_{D}}}$$

Table S3.7: GAE model kinetic parameters

| **Parameter** | **Definition** | **Value** |
| --- | --- | --- |
| $T_{\max f,GAE}$ | Maximum forward transport rate | Tissue-specific |
| *K_A_, K_C_* | *ASP* binding constant | $0.12\times10^{-3} M$ |
| *K_B_, K_D_* | *GLU* binding constant | $0.25\times10^{-3} \times K_{H}M$ |
| *K_H_* | Proton binding constant | $0.1\times10^{-6} M$ |

### Transport 8: ATP-ADP Exchange (Adenine Nucleotide Translocase, ANT)

*ADP_e_ + ATP_m_ ⇌ ADP_m_ + ATP_e_*

For this reaction, the reactants and products are denoted as $A=ADP_{e}, B=ATP_{m}, C=ADP_{m}$, and $D=ATP_{e}$. The corresponding flux equation, $J_{ANT}$, is given by:

$$J_{ANT}=\frac{T_{\max f,ANT}\left(\exp\left(\frac{\beta_{ANT}F\Delta\psi}{RT}\right)\frac{C_{A}C_{B}}{K_{A}K_{B}}-\exp\left(\frac{\left(\beta_{ANT}-1\right)F\Delta\psi}{RT}\right)\frac{C_{C}C_{D}}{K_{C}K_{D}}\right)}{\left(1+\frac{C_{C}}{K_{C}}+\frac{C_{B}}{K_{B}}\exp\left(\frac{\beta_{ANT}F\Delta\psi}{RT}\right)\right)\left(1+\frac{C_{A}}{K_{A}}+\frac{C_{D}}{K_{D}}\exp\left(\frac{\left(\beta_{ANT}-1\right)F\Delta\psi}{RT}\right)\right)}$$

Table S3.8: ANT model kinetic parameters

| **Parameters** | **Definition** | **Value** |
| --- | --- | --- |
| $T_{\max f,ANT}$ | Maximum forward transport rate | Tissue-specific |
| *K_A_, K_C_* | *ADP* binding constant | $5.1\times10^{-6} M$ |
| *K_B_, K_D_* | *ATP* binding constant | $5.7\times10^{-6} M$ |
| *β_ANT_* | *ANT* free energy barrier fraction | 0.6 |

### Transport 9: Inorganic Phosphate Carrier (PIC)

*Pi_e_ + H_e_^+^ ⇌ Pi_m_ + H_m_^+^*

For this reaction, the reactants and products are denoted as $A=Pi_{e}, B=H_{e}^{+}, C=Pi_{m}$, and $D=H_{m}^{+}$. The corresponding flux equation, $J_{PIC}$, is given by:

$$J_{PIC}=\frac{T_{\max f,PIC}\left(\frac{C_{A}C_{B}}{K_{A}K_{B}}-\frac{C_{C}C_{D}}{K_{C}K_{D}}\right)}{1+\frac{C_{A}}{K_{A}}+\frac{C_{B}}{K_{B}}+\frac{C_{C}}{K_{C}}+\frac{C_{D}}{K_{D}}+\frac{C_{A}C_{B}}{K_{A}K_{B}}+\frac{C_{C}C_{D}}{K_{C}K_{D}}}$$

Table S3.9: PIC model kinetic parameters

| **Parameters** | **Definition** | **Value** |
| --- | --- | --- |
| $T_{\max f,PIC}$ | Maximum forward transport rate | Tissue-specific |
| *K_A_, K_C_* | *Pi* binding constant | $9.4\times10^{-3} M$ |
| *K_B_, K_D_* | *H^+^* binding constant | $0.1\times10^{-6} M$ |

### Transport 10: Passive Proton Leak

$$H_{e}^{+}\rightleftharpoons H_{m}^{+}$$

For this reaction, the reactants and products are denoted as *A =* $H_{e}^{+}$ and *B =* $H_{m}^{+}$. The proton leak flux equation, $J_{HLeak}$, is given by:

$$J_{HLeak}=\frac{T_{\max f,HLeak}}{K_{H}}\left(C_{A}\exp\left(\frac{\beta_{HLeak}F\Delta\Psi}{RT}\right)-C_{B}\exp\left(-\frac{(\beta_{HLeak}-1)F\Delta\Psi}{RT}\right)\right)$$

Table S3.10: Proton leak model kinetic parameters

| **Parameters** | **Definition** | **Value** |
| --- | --- | --- |
| $T_{\max f,HLeak}$ | Maximum forward transport rate | Tissue-specific |
| *K_H_* | *H^+^* binding constant | $0.1\times10^{-6} M$ |
| *β_HLeak_* | Proton leak free energy barrier | 1.0 |

## Part S4: Governing mass balance equations for the integrated mitochondrial bioenergetics model

***Extra-mitochondrial (buffer) region:***

| $V_{e}\frac{dC_{Pi_{e}}}{dt}=-J_{PIC}+J_{DCCM}+J_{DCCS}$ |  |
| --- | --- |
| $V_{e}\frac{dC_{ADP_{e}}}{dt}=-J_{ANT}$ |  |
| $V_{e}\frac{dC_{ATP_{e}}}{dt}=+J_{ANT}$ |  |
| $V_{e}\frac{dC_{PYR_{e}}}{dt}=-J_{PYRH}$ |  |
| $V_{e}\frac{dC_{MAL_{e}}}{dt}=-J_{OME}-J_{DCCM}-J_{TCC}$ |  |
| $V_{e}\frac{dC_{CIT_{e}}}{dt}=+J_{TCC}$ |  |
| $V_{e}\frac{dC_{AKG_{e}}}{dt}=+J_{OME}$ |  |
| $V_{e}\frac{dC_{SUC_{e}}}{dt}=-J_{DCCS}$ |  |
| $V_{e}\frac{dC_{GLU_{e}}}{dt}=-J_{GLUH}-J_{GAE}$ |  |
| $V_{e}\frac{dC_{ASP_{e}}}{dt}=+J_{GAE}$ |  |

***Inter-membrane space (IMS) region:***

| $V_{i}\frac{dC_{CytC_{o}}}{dt}=-2J_{CIII}+2J_{CIV}$ |  |
| --- | --- |
| $\frac{dC_{CytC_{r}}}{dt}=-\frac{dC_{CytC_{o}}}{dt}$ |  |

***Mitochondrial matrix region:***

| $V_{m}\frac{dC_{Pi_{m}}}{dt}=J_{PIC}-J_{SCAS}-J_{CV}-J_{DCCM}-J_{DCCS}$ |  |
| --- | --- |
| $V_{m}\frac{dC_{ATP_{m}}}{dt}=-J_{ANT}+J_{NDK}+J_{CV}$ |  |
| $V_{m}\frac{dC_{ADP_{m}}}{dt}=-V_{m}\frac{dC_{ATP_{m}}}{dt}$ |  |
| $V_{m}\frac{dC_{GTP_{m}}}{dt}=J_{SCAS}-J_{NDK}$ |  |
| $V_{m}\frac{dC_{GDP_{m}}}{dt}=-V_{m}\frac{dC_{GTP_{m}}}{dt}$ |  |
| $V_{m}\frac{dC_{NADH_{m}}}{dt}=+J_{P\text{DH}}+J_{ICDH}+J_{AKGDH}+J_{MDH}-J_{CI}$ |  |
| $V_{m}\frac{dC_{NAD_{m}^{+}}}{dt}=-V_{m}\frac{dC_{NADH_{m}}}{dt}$ |  |
| $V_{m}\frac{dC_{UQ_{m}}}{dt}=-J_{CI}-J_{CII}+J_{CIII}$ |  |
| $V_{m}\frac{dC_{UQH_{2m}}}{dt}=-V_{m}\frac{dC_{UQ_{m}}}{dt}$ |  |
| $V_{m}\frac{dC_{CytCox}}{dt}=-2J_{CIII}+2J_{CV}$ |  |
| $V_{m}\frac{dC_{Cytred}}{dt}=-V_{m}\frac{dC_{Cytox}}{dt}$ |  |
| $V_{m}\frac{dC_{GLU_{m}}}{dt}=J_{GOT}+J_{GAE}+J_{GLUH}$ |  |
| $V_{m}\frac{dC_{ASP_{m}}}{dt}=-J_{GOT}-J_{GAE}$ |  |
| $V_{m}\frac{dC_{PYR_{m}}}{dt}=J_{PYRH}-J_{PDH}$ |  |
| $V_{m}\frac{dC_{OXA_{m}}}{dt}=J_{MDH}-J_{CITS}+J_{GOT}$ |  |
| $V_{m}\frac{dC_{CIT_{m}}}{dt}=+J_{CITS}-J_{ICDH}-J_{TCC}$ |  |
| $V_{m}\frac{dC_{AKG_{m}}}{dt}=J_{ICDH}-J_{AKGDH}-J_{GOT}-J_{OME}$ |  |
| $V_{m}\frac{dC_{SCA_{m}}}{dt}=J_{AKGDH}-J_{SCAS}$ |  |
| $V_{m}\frac{dC_{SUC_{m}}}{dt}=J_{SCAS}-J_{S\text{DH}}+J_{DCCS}$ |  |
| $V_{m}\frac{dC_{FUM_{m}}}{dt}=J_{S\text{DH}}-J_{FH}$ |  |
| $V_{m}\frac{dC_{MAL_{m}}}{dt}=J_{FH}-J_{MDH}+J_{DCCM}+J_{TCC}+J_{OME}$ |  |
| $V_{m}\frac{dC_{ACoA_{m}}}{dt}=J_{PDH}-J_{CITS}$ |  |
| $V_{m}\frac{dC_{CoA_{m}}}{dt}=-J_{P\text{DH}}+J_{CITS}+J_{SCAS}-J_{AKG\text{DH}}$ |  |

**Mitochondria membrane potential:**

| $\frac{d\Delta\Psi}{dt}=\frac{1}{C_{imm}}\left(4J_{CI}+2J_{CIII}+4J_{CIV}-J_{ANT}-J_{Hleak}-J_{GAE}-nHJ_{CV}\right)$ |  |
| --- | --- |

where $C_{\text{imm}}$ is the capacitance of the inner mitochondrial membrane (IMM).

$C_{\text{imm}}=6.75\times10^{-6}(V_{m}+V_{i})$ mol/mV

**Oxygen and carbon dioxide concentrations:**

For membrane potential experiments using isolated mitochondria in the PTI spectrofluorometer, the PTI chamber is open to the atmosphere, and hence oxygen and carbon dioxide concentrations in the chamber are assumed to be constant (equilibrated with the atmospheric oxygen and carbon dioxide). Therefore,

| $\frac{dC_{O_{2}}}{dt}=0 \text{and} \frac{dC_{CO_{2}}}{dt}=0$ |  |
| --- | --- |

For respiration experiments using isolated mitochondria in the Oroboros oxygraph-2k (O2k) instruments, the Oroboros chamber is a closed system, and hence oxygen decay in the chamber is due to oxygen consumption by the mitochondria at complex IV. Correspondingly, total carbon dioxide (i.e., CO_2_ dissolved (free) in the water space of different regions and CO_2_ in bicarbonate (HCO_3_^-^) form in different regions) would rise due to its production through the PDH, ICDH, and AKGDH reactions in the mitochondrial matrix. Therefore,

| $\left(V_{m}+V_{e}+V_{i}\right)\frac{dC_{O_{2}}}{dt}=-0.5 J_{CIV}$ $\left(\left(V_{m}+V_{e}+V_{i}\right)+\frac{K_{CO2H}K_{H2CO3}}{C_{H_{m}^{+}}}\left(V_{m}+V_{e}+V_{i}\right)\right)\frac{dC_{CO_{2}}}{dt}=J_{PDH}+J_{ICDH}+J_{AKGDH}$ |  |
| --- | --- |

where *K_CO2H_* = 2.7E-3 (unitless) is the equilibrium constant for the CO_2_ hydration reaction (CO_2_ + H_2_O *⇌* H_2_CO_3_) and *K_H2CO3_* = 2.4E-4 Molar is the equilibrium constant for the H_2_CO_3_ dissociation reaction (H_2_CO_3_ *⇌* HCO_3_^-^ + H^+^). H_2_CO_3_ and CO_3_^2-^ are usually negligible under physiological conditions and are ignored.

In isolated mitochondrial respirometry experiments using the Oroboros O2k system, the initial total CO_2_ concentration is negligible (since *P*_CO2_ is 0.3 mmHg). During experiments, total CO_2_ production will be equal to total O_2_ consumption, which will be a maximum of initial total O_2_ concentration (~200 μM).

**Table S4.1: Initial conditions for state variables in mitochondrial matrix and buffer regions**

| **Biochemical Species** | **Initial Conditions (M)** |
| --- | --- |
| ADP_m_ | 2.5×10^-4^ |
| ADP_e_ | 0 |
| ATP_m_ | 9.75×10^-3^ |
| ATP_e_ | 0 |
| GDP_m_ | 2.5×10^-5^ |
| GTP_m_ | 9.75×10^-4^ |
| Pi_m_ | 10×10^-3^ |
| Pi_e_ | 5×10^-3^ |
| NAD^+^ | 7.5×10^-4^ |
| NADH | 2.25×10^-3^ |
| UQ | 3.75×10^-4^ |
| UQH_2_ | 1.12×10^-3^ |
| CytCo | 1.5×10^-3^ |
| CytCr | 1.5×10^-3^ |
| PYR_m_ | 10×10^-3^ |
| PYR_e_ | 0 |
| ACoA_m_ | 10×10^-3^ |
| CoA_m_ | 10×10^-3^ |
| CIT_m_ | 10×10^-3^ |
| CIT_e_ | 0 |
| GLU_m_ | 10×10^-3^ |
| GLU_e_ | 0 |
| ASP_m_ | 10×10^-3^ |
| ASP_e_ | 0 |
| OXA_m_ | 10×10^-3^ |
| αKG_m_ | 10×10^-3^ |
| αKG_e_ | 0 |
| SCoA_m_ | 10×10^-3^ |
| SUC_m_ | 10×10^-3^ |
| SUC_e_ | 0 |
| FUM_m_ | 10×10^-3^ |
| MAL_m_ | 10×10^-3^ |
| MAL_e_ | 0 |
| O_2,m_ | 210×10^-6^ |

## Part S5. Model parameter analysis and corroborations figures

**Figure S1.** **Timeline experimental protocols for isolated mitochondrial respiration and $\boldsymbol{\Delta}\boldsymbol{\Psi}_{\boldsymbol{m}}$.** (A) Experimental protocol for OCR measurements in the mitochondria isolated from Sprague-Dawley rat heart, kidney cortex, and kidney OM. In this protocol, at time (t) = 0 min, 0.05 mg/mL of the heart mitochondria or 0.2 mg/mL of the kidney cortex or OM mitochondria were added to the respiration buffer. At t=2 min, one of the following substrate combinations PM (5:2.5mM), GM (5:2.5mM), AM (5:2.5mM), and SUC±ROT (10mM±0.5$\mu$M) were added, followed by sequential addition of increasing ADP concentrations including 25, 50, 75, 100, 150, and 250 $\mu$M. This protocol was used for model parameterization. (B) Experimental protocol for measuring time-courses of mitochondrial respiration and $\Delta\Psi_{m}$ in the mitochondria isolated from SD rat heart, kidney cortex, and kidney OM. In this protocol, at time (t) = 0 min, 0.1 mg/mL of the heart mitochondria or 0.2 mg/mL of the kidney cortex or OM mitochondria were added to the respiration buffer. At t=2 min, one of the following substrate combinations PM (5:2.5mM), GM (5:2.5mM), AM (5:2.5mM), and SUC±ROT (10mM±0.5$\mu$M) were added, followed by single addition of 200 $\mu$M ADP to the heart mitochondria and 100 $\mu$M ADP to the kidney cortex and OM mitochondria. This protocol was used for model validation.

**Figure S2. Estimated tissue-specific extrinsic model parameters.** The estimated optimized values for the maximum reaction velocities (*V*_max_) of 9 TCA enzymes, 4 ETC complexes, and ATP synthase, and the maximum transport velocities (*T*_max_) of 10 metabolite transporters in the heart (A), kidney cortex (B), and kidney OM (C).

**Figure S3. Normalized sensitivity coefficients of the objective function with respect to the optimal model parameters.** The normalized sensitivity coefficients for the models’ extrinsic parameters in the heart (A), kidney cortex (B), and kidney OM (C) mitochondria. The models’ normalized sensitivity coefficients were calculated with ±1% change in the estimated parameter values from their optimized values. A parameter contribution to the model solution is proportional to its normalized sensitivity coefficient.

**Figure S4. Sensitivity of the objective function in response to variations in the optimal model parameters.** The changes in the models’ normalized sum of squared errors objective function (E/E_0_) in response to ±50% change in the estimated parameter values from their optimized values (P/P_0_) in the heart (A-C), kidney cortex (D-F), and kidney OM (G-I) mitochondria.

**Figure S5. Parameter correlation coefficient matrix.** The calculated correlation coefficient matrix for the 24 estimated optimal parameters in the heart (A), kidney cortex (B), and kidney OM (C). The correlation coefficients are in the range of -1 to +1 indicated with red color showing positive correlation in the range of 0 to +1, the blue color showing negative correlation in the range of 0 to -1, and the darker colors show higher correlation close to ±1. The diagonal of the matrix shows the correlation of each parameter with itself which is 1. Also, since the matrix is symmetric, the upper triangle is put values equal to 0 for simplicity.

**Figure S6. Model predictions of metabolic fluxes.** Models’ predictions of the time courses of fluxes of metabolite transporters, TCA enzyme reactions, ETC complexes, and ATP synthase in the heart (A and B), kidney cortex (C and D), and kidney OM (E and F) mitochondria in response to additions of increasing ADP concentrations of 25, 50, 75, 100, 150, and 250 $\mu$M. The mitochondrial fluxes are predicted in the presence of five different substrate combinations. Figures A, C and E show time course of mitochondrial fluxes predicted in the presence of NADH-linked substrates PM (5:2.5mM), AM (5:2.5mM), and GM (5:2.5mM) for the heart (A), kidney cortex (C), and kidney OM (E) mitochondria. Figures B, D and F show time course of mitochondrial fluxes predicted in the presence of FADH_2_-linked substrate SUC(±ROT) (10mM(±.5$\mu$M)) for the heart (B), kidney cortex (D), and kidney OM (F) mitochondria. The mitochondrial fluxes are predicted in the unit of nmol/min/mg mitochondria in the heart and kidney cortex and OM.

**Figure S7. Model predictions of metabolite concentrations.** Models’ predictions of the time courses of mitochondrial metabolite concentrations in the heart (A and B), kidney cortex (C and D), and kidney OM (E and F) in response to additions of increasing ADP concentrations of 25, 50, 75, 100, 150, and 250 $\mu$M. The mitochondrial metabolite concentrations are predicted in the presence of five different substrate combinations. Figures A, C and E show time course of mitochondrial metabolite concentrations predicted in the presence of NADH-linked substrates including PM (5:2.5mM), AM (5:2.5mM), and GM (5:2.5mM) for the heart (A), kidney cortex (C), and kidney OM (E). Figures B, D and F show time course of mitochondrial metabolite concentrations predicted in the presence of FADH_2_-linked substrate SUC(±ROT) (10mM(±.5$\mu$M)) in the heart (B), kidney cortex (D), and kidney OM (F).

## REFERENCES

1. Zhang X, Dash RK, Jacobs ER, Camara AKS, Clough AV, Audi SH. Integrated computational model of the bioenergetics of isolated lung mitochondria. *PLoS One.* 2018;13 (6):e0197921. doi: 10.1371/journal.pone.0197921.

2. Zhang X, Dash RK, Clough AV, Xie D, Jacobs ER, Audi SH. Integrated Computational Model of Lung Tissue Bioenergetics. *Frontiers in physiology.* 2019;10:191. doi: 10.3389/fphys.2019.00191.

3. Li X, Dash RK, Pradhan RK, et al. A database of thermodynamic quantities for the reactions of glycolysis and the tricarboxylic acid cycle. *J Phys Chem B.* 2010;114 (49):16068-16082. doi: 10.1021/jp911381p.

4. Li X, Wu F, Qi F, Beard DA. A database of thermodynamic properties of the reactions of glycolysis, the tricarboxylic acid cycle, and the pentose phosphate pathway. *Database (Oxford).* 2011;2011:bar005. doi: 10.1093/database/bar005.

5. Alberty RA. *Thermodynamics of Biochemical Reactions.* Hoboken, N.J.: John Wiley & Sons; 2003.

6. Bazil JN, Beard DA, Vinnakota KC. Catalytic Coupling of Oxidative Phosphorylation, ATP Demand, and Reactive Oxygen Species Generation. *Biophys J.* 2016;110 (4):962-971. doi: 10.1016/j.bpj.2015.09.036.

7. Heyde E, Ainsworth S. Kinetic studies on the mechanism of the malate dehydrogenase reaction. *The Journal of biological chemistry.* 1968;243 (9):2413-2423. Published 1968/05/10.

8. Dash RK, Beard DA. Analysis of cardiac mitochondrial Na(+)–Ca(2+) exchanger kinetics with a biophysical model of mitochondrial Ca(2+) handing suggests a 3: 1 stoichiometry. *The Journal of physiology.* 2008;586 (Pt 13):3267-3285. doi: 10.1113/jphysiol.2008.151977.
